# MST3 Kinase Phosphorylates TAO1/2 to Enable Myosin Va Function in Promoting Spine Synapse Development

**DOI:** 10.1016/j.neuron.2014.10.025

**Published:** 2014-12-03

**Authors:** Sila K. Ultanir, Smita Yadav, Nicholas T. Hertz, Juan A. Oses-Prieto, Suzanne Claxton, Alma L. Burlingame, Kevan M. Shokat, Lily Y. Jan, Yuh-Nung Jan

**Affiliations:** 1 Departments of Physiology, Biochemistry, and Biophysics, University of California, San Francisco, San Francisco, CA 94158, USA; 2 Department of Cellular and Molecular Pharmacology, University of California, San Francisco, San Francisco, CA 94158, USA; 3 Department of Pharmaceutical Chemistry, University of California, San Francisco, San Francisco, CA 94158, USA; 4 Howard Hughes Medical Institute, University of California, San Francisco, San Francisco, CA 94158, USA; 5 Medical Research Council, National Institute for Medical Research, The Ridgeway, Mill Hill, London NW7 1AA, UK

## Abstract

Mammalian Sterile 20 (Ste20)-like kinase 3 (MST3) is a ubiquitously expressed kinase capable of enhancing axon outgrowth. Whether and how MST3 kinase signaling might regulate development of dendritic filopodia and spine synapses is unknown. Through shRNA-mediated depletion of MST3 and kinase-dead MST3 expression in developing hippocampal cultures, we found that MST3 is necessary for proper filopodia, dendritic spine, and excitatory synapse development. Knockdown of MST3 in layer 2/3 pyramidal neurons via in utero electroporation also reduced spine density in vivo. Using chemical genetics, we discovered thirteen candidate MST3 substrates and identified the phosphorylation sites. Among the identified MST3 substrates, TAO kinases regulate dendritic filopodia and spine development, similar to MST3. Furthermore, using stable isotope labeling by amino acids in culture (SILAC), we show that phosphorylated TAO1/2 associates with Myosin Va and is necessary for its dendritic localization, thus revealing a mechanism for excitatory synapse development in the mammalian CNS.

## Highlights

• MST3 loss of function increases filopodia and reduces spine synapses in neurons
• 13 MST3 substrates including TAO1/2 are identified using chemical genetics
• Phosphorylation of TAO kinases is critical for filopodia/spine formation
• TAO1/2 binds Myosin Va at T440/475 and regulates its recruitment in dendrites

## Teaser

Ultanir et al. show that MST3 kinase is required for normal excitatory synapse development. Using chemical genetics they identify TAO1/2 kinase as a direct substrate of MST3 that upon phosphorylation associates with Myosin Va and regulates synapse formation.

## Introduction

Dendrite arborization and synapse formation are critical for wiring the neural circuitry (Jan and Jan, 2010; Parrish et al., 2006). Dendrites of pyramidal neurons, the predominant excitatory neurons in the mammalian brain, contain dendritic spines, postsynaptic structures harboring more than 90% of excitatory synapses in the brain (Harris and Kater, 1994; Nimchinsky et al., 2002). Dendritic spine formation is preceded by actin-rich filopodia that typically contain immature synapses and are thought to be involved in dendrite arborization and synaptogenesis (Fiala et al., 1998; Yuste and Bonhoeffer, 2004). Unraveling the molecular mechanisms underlying spine formation is an important research area. Alterations in many proteins implicated in neural development have been linked to neurological disorders, such as autism (Huguet et al., 2013). A better understanding of the molecular mechanisms involved in brain development and synapse formation could enable future therapeutic interventions.

Protein kinases regulate a wide range of cellular processes by phosphorylating and altering the function of their target molecules. There are more than 500 kinases in the human genome. How various kinases regulate neuronal development remains poorly understood. Previous studies by us and others have revealed that the kinase cascade of the Hippo, Wts, and Trc (mammalian nuclear Dbf2-related [NDR] 1/2) kinases play important and evolutionarily conserved roles in dendrite morphogenesis (Emoto et al., 2004, 2006; Gallegos and Bargmann, 2004; Ultanir et al., 2012; Zallen et al., 2000).

Mammalian Sterile 20 (Ste20)-like kinase 3 (MST3) belongs to the highly conserved family of Ste20-like kinases that includes Hippo as the most well studied member. MST3, also known as Serine/threonine kinase 24, is in the subfamily of the germinal centre kinase III kinases that contain an ∼275 amino acids long N-terminal kinase domain and a C-terminal regulatory domain. Originally identified as a kinase with requirement of manganese as a preferred cofactor (Schinkmann and Blenis, 1997), MST3 is predominantly localized to the cytoplasm (Preisinger et al., 2004; Schinkmann and Blenis, 1997). MST3a is the shorter 431 amino acid isoform, which differs from the 443 amino acid MST3b in its 16 N-terminal amino acids. MST3 activation can result from autophosphorylation of a threonine in the N-terminal kinase domain (Schinkmann and Blenis, 1997), dephosphorylation of a threonine in the C-terminal regulatory domain to enable binding of the scaffolding protein MO25 (Fuller et al., 2012), or caspase mediated cleavage between these two domains, leading to nuclear localization of the kinase domain (Huang et al., 2002).

MST3 is expressed ubiquitously in various tissues including the brain (Irwin et al., 2006; Schinkmann and Blenis, 1997). MST3 signaling is involved in hypoxia-induced apoptosis in trophoblasts, where MST3 can activate Caspase 3 (Wu et al., 2008, 2011). MST3b was isolated as a purine-sensitive kinase, which facilitates axon outgrowth in response to inosine (Irwin et al., 2006). MST3b also facilitates axon regeneration in cultures and in vivo (Lorber et al., 2009), and MST3 is required for radial neuronal migration in the developing cortex (Tang et al., 2014). It is unknown whether MST3 plays a role in dendrites, dendritic filopodia, and spine morphogenesis. MST3′s mechanism of action is also an open question.

The homology of MST3 to the Hippo kinase in *Drosophila* prompted us to test whether MST3 signaling has a role in mammalian dendrite development. By inhibiting MST3 activity via expression of a kinase-dead form of MST3 or small hairpin (sh)RNA mediated knockdown of MST3 in dissociated hippocampal neuronal cultures, we show that MST3 is required for the formation and maintenance of dendritic architecture. We found that MST3 limits dendritic filopodia and facilitates the formation of spines harboring mature synapses. In utero electroporation of MST3 shRNA also confirmed that MST3 is required for dendritic spine morphogenesis in vivo. In order to investigate the molecular mechanisms by which MST3 regulates dendritic architecture, we used chemical genetics and mass spectrometry and identified 13 potential substrates of MST3. Next, by expressing phospho mutant and phospho-mimetic forms of a subset of these novel substrates, we found that expression of phospho mutant TAO1 or TAO2, or shRNA mediated knockdown of TAO1/2, is sufficient to cause increased dendritic filopodia and loss of spines, reminiscent of MST3 loss of function phenotype. We further show that downstream of TAO1/2, a Myosin Va complex specifically binds to the critical phosphorylation site found on both TAO1 and TAO2. Thus, MST3 kinase functions in the formation of dendritic spines by regulating several downstream effectors. In particular, MST3′s phosphorylation of TAO1/2, an autism spectrum disorder susceptibility gene, is critical for its regulation of Myosin Va dependent trafficking in dendrites.

## Results

### MST3 Limits Filopodia on Developing Dendrites

**Figure 1:**
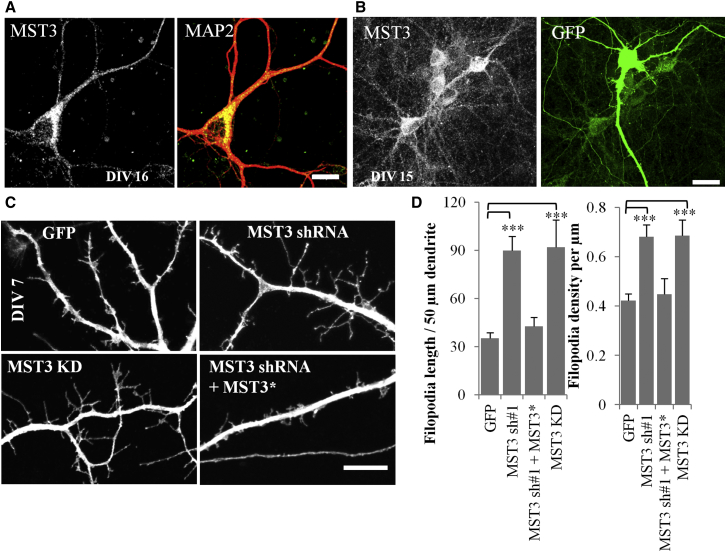
MST3 Is Required for Limiting Dendritic Filopodia in Hippocampal Cultures (A) Image shows MAP2 and MST3 immunostaining of a DIV16 hippocampal neuron; MST3 is found in the cell body and dendrites. Scale bar represents 10 μm. (B) Hippocampal cultures are transfected with GFP and immunostained with a monoclonal MST3 antibody generated in mouse. Scale bar represents 25 μm. (C) Representative images of GFP expressing hippocampal neuron dendrites at DIV7. Confocal stacks were obtained and projected at maximum intensity. Scale bar represents 7.5 μm. (D) MST3 shRNA and MST3 KD expression leads to increased filopodia length per 50 μm of dendrite and filopodia density, and shRNA effects can be rescued with shRNA resistant MST3 (MST3^∗^) (N = 14, 15, 11, and 6, respectively in columns in graphs). In all figures ^∗^ = p < 0.05, ^∗∗^ = p < 0.01, and ^∗∗∗^ = p < 0.001, in Student’s t test. All error bars reflect SEM. See also Figure S1.

Localization of endogenous MST3 in punctate structures in the neuronal soma and dendrites was evident from immunofluorescence staining of dissociated hippocampal neurons double labeled with the dendrite marker microtubule-associated protein 2 (MAP2) (Figure 1A), as well as immunofluorescence staining of GFP transfected neurons in high density cultures with monoclonal antibody against MST3 (Figure 1B). The immunofluorescence staining was dramatically reduced by shRNA knockdown of MST3, indicating the staining is specific to MST3 kinase (Figures S1A and S1B available online). Similar cytoplasmic distribution of Hemagglutinin (HA)-tagged MST3 proteins expressed in hippocampal neurons could be detected by anti-HA immunocytochemistry (Figure S1G).

To find out if MST3 is required for postnatal dendritic development, we transfected neurons at 3 days in vitro (DIV3) with GFP plasmid alone as control, or a plasmid with GFP and MST3 shRNA expressed via separate promoters, or mutant MST3 and GFP expressing plasmids. GFP expressing control neurons at DIV7 displayed dendritic filopodia (Figure 1C). Inhibiting MST3 function by expressing either MST3 shRNA or MST3 kinase dead (K53R) mutant (MST3-KD) together with GFP resulted in a dramatic increase in the total dendritic filopodia length per 50 μm dendrite and filopodia density (Figures 1C and 1D), as well as average filopodia length (Figure S1C). As expected for effects due to loss of MST3 function, these phenotypes could be rescued by coexpressing MST3 shRNA with shRNA-resistant MST3 (MST3^∗^) (Figures 1C, 1D, and S1C), and they could be produced by two different MST3 shRNAs (Figure S1H) that effectively knocked down endogenous MST3 (Figures S1F and S4F), but not by expression of MST3^∗^ or a control shRNA (Figures S1D and S1E). It is conceivable that MST3-KD might be acting as a dominant-negative form for MST3 function via binding and titrating coactivators. These data indicate that MST3 is required in developing dendrites to limit dendritic filopodia formation during early postnatal development.

### MST3 Is Required for Spine Formation and Maintenance

**Figure 2:**
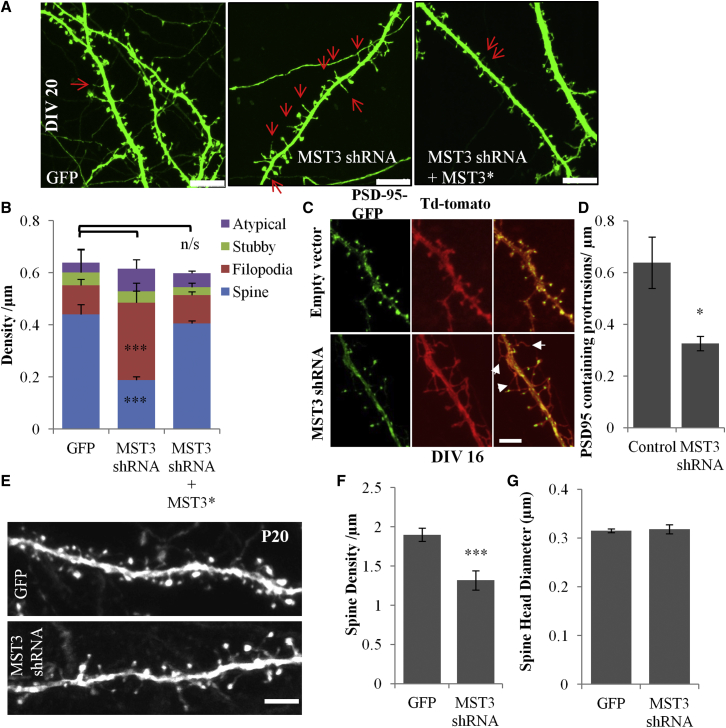
MST3 Is Required for Spine Development in Hippocampal Cultures and In Vivo (A) Hippocampal neurons were transfected at DIV16 and imaged at DIV20. Scale bar represents 7.5 μm. (B) Filopodia density is increased and spine density is reduced in MST3 shRNA expressing neurons. The effect was rescued with shRNA resistant MST3^∗^ expression (n of cells = 11, 12, and 6, respectively). (C and D) Hippocampal neurons were transfected with PSD-95-GFP and Td-tomato alone or Td-tomato together with MST3 shRNA at DIV13 and fixed and imaged at DIV16. Density of protrusions containing PSD-95-GFP puncta is reduced in MST3 shRNA expressing neurons (n of cells = 5 for each condition). Arrows point to filopodia that do not contain PSD-95. Scale bar represents 5 μm. E. MST3 shRNA or GFP alone is expressed in layer 2/3 neurons via in utero electroporations at E14.5. Brain sections are analyzed at P20. Representative basal dendrite images of layer 2/3 neurons expressing GFP. Scale bar represents 3 μm. (F and G) Spine density is reduced and spine head diameter is not changed in MST3 shRNA expressing neurons’ basal dendrites (n of cells = 2 and 7, respectively). All error bars reflect SEM. See also Figure S2.

To test if MST3 is also required later in development for the formation or maintenance of dendritic spines, we transfected neurons with shRNA constructs at DIV16 when spine formation has largely taken place, and observed spines at DIV20. MST3 shRNA expression resulted in a robust reduction of dendritic spines and an increase in dendritic filopodia, which could be rescued by expressing MST3^∗^ (Figures 2A and 2B). Moreover, coexpression of MST3 shRNA together with the postsynaptic marker PSD-95-GFP and Td-tomato led to a reduction of the density of PSD-95-GFP containing dendritic protrusions (Figures 2C and 2D), while total PSD-95-GFP intensity in dendrites and spines remained constant (data not shown). The density of dendritic protrusions that colocalized with endogenous synapsin or PSD-95 was reduced in MST3 shRNA expressing neurons as well (Figures S2A and S2B). These data indicate that MST3 is required for maintaining the structure of dendritic spines and excitatory synapses in cultures.

To test whether MST3 alters spine morphogenesis in vivo, we expressed MST3 shRNA in developing layer 2/3 cortical neurons in mice via in utero electroporation at embryonic day (E)14.5 in mice (Figure 2E). We found no change in spine head diameter (Figure 2G), but a significant reduction in spine density in MST3 shRNA expressing neurons compared to control neurons expressing GFP alone (Figures 2E and 2F). Taken together, these findings indicate MST3 kinase signaling is important for the formation and maintenance of spines and excitatory synapses.

### Identification of MST3 Phosphorylation Substrates Using Chemical Genetics

**Figure 3:**
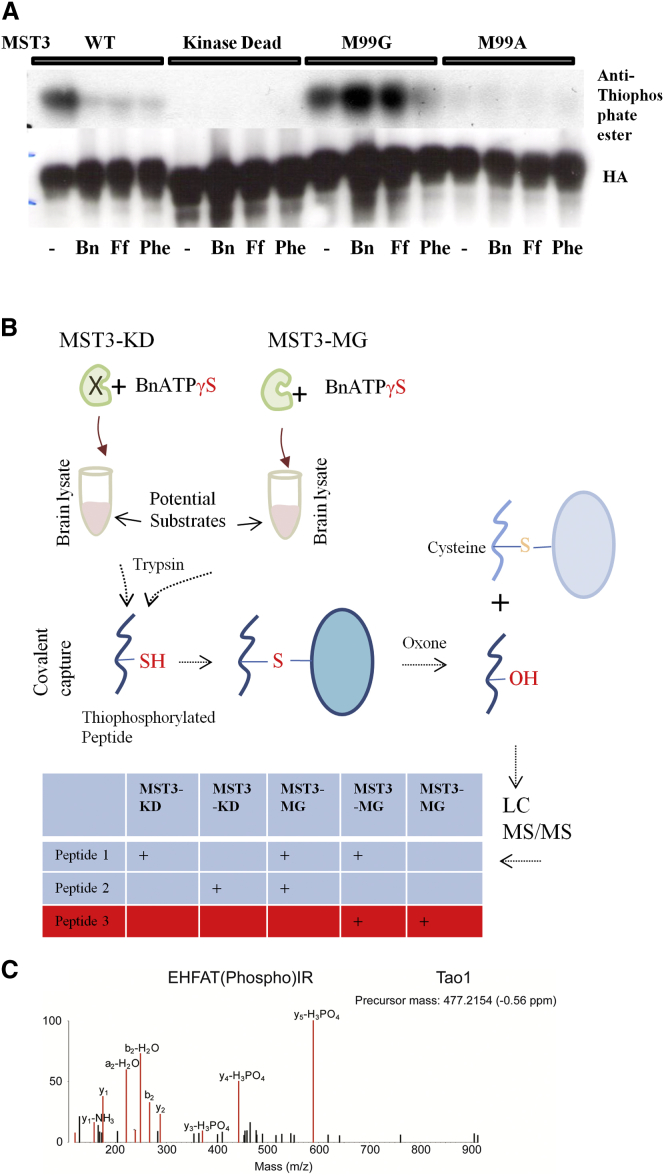
MST3 Substrate Identification Using Chemical Genetics (A) MST3-MG can use Benzyl-ATP-γ−S to autophosphorylate. HA tagged MST3 wt, KD, and analog sensitive mutants (MG and MA) purified from HEK293 cells and subjected to kinase assay using ATP-γ-S analogs. Autophosphorylation of HA-MST3 is used as a readout of MST3 activity in kinase assays conducted with various ATP-γ-S analogs: Benzyl-ATP-γ-S (Bn), Furfuryl-ATP-γ-S (Ff), and Phenyl-ethyl-ATP-γ-S (Phe). Total MST3 levels are shown by anti-HA blot. MST3 M99G can use Bn efficiently. Kinase assay is detected using PNBM alkylation and anti-thiophosphate ester blot. (B) Simplified scheme of kinase substrate labeling and identification (Hertz et al., 2010). (C) MS spectra shows the detected phosphopeptide that belongs to the substrate TAO1. See also Figure S3.

To identify downstream effectors of MST3, we used a chemical genetic method for kinase substrate identification (Blethrow et al., 2004; Hertz et al., 2010) used previously to identify the substrates of NDR 1/2 (Ultanir et al., 2012). We generated two ATP-analog sensitive MST3 mutants, MST3 M99G and MST3 M99A, by mutating the “gatekeeper” residue at the ATP binding pocket to allow for utilization of bulkier ATP analogs (Blethrow et al., 2004). In order to determine if the MST3 analog-sensitive mutants can utilize a bulky ATP analog with high efficiency, we used the autophosphorylation activity of MST3 as a readout in kinase assays using ATP-γ-S, Benzyl-ATP-γ-S, Furfuryl-ATP-γ-S, or Phenyl-Ethyl-ATP-γ-S (Figure 3A). Thiophosphorylated substrate, in this case MST3 itself, was alkylated by p-nitrobenzylmesylate (PNBM) for detection via an anti-thiophosphate ester antibody (Allen et al., 2007). Because the MST3 M99G analog sensitive kinase was active with a preference toward Benzyl-ATP-γ-S, we used this combination to phosphorylate and then detect MST3 substrates present in the brain.

To undertake covalent capture for kinase substrate identification (Blethrow et al., 2008; Hengeveld et al., 2012; Ultanir et al., 2012) (Figure 3B), we purified HA tagged MST3 M99G and MST3-KD that were expressed in COS-7 cells via immunoprecipitation using HA antibody bound-beads (Figure S3A) and labeled brain lysates from postnatal day (P) 8–9 mice with Benzyl-ATP-γ-S, with or without MST3 M99G or MST3-KD, for 1–2 hr at 30°C. For purification of thiophosphorylated peptide fragments of potential MST3 targets using the three-step covalent capture protocol, (1) proteins in the brain lysate were digested into tryptic fragments, (2) Thiol containing peptides (including those containing cysteine amino acids) were captured onto iodoacetyl agarose beads, and (3) thiophosphate ester linked peptides were released from the beads by an oxone induced hydrolysis reaction as phospho-peptides, while cysteine linked peptides remained on beads. Eluted peptides and phosphorylation sites were identified in liquid chromatography/tandem mass spectrometry analysis. Phospho-peptides detected more than once in MST3 M99G experimental samples and not in any of the negative controls are included in a list of candidates (Figure 3B). Examples of mass spectra for identified MST3 substrates TAO1 (Figure 3C), EPS8, and TAO2 (Figures S3B and S3C) are shown.

### Several MST3 Phosphorylation Substrates Are Cytoskeleton Regulators

Of 13 potential MST3 substrates identified along with their phosphorylation sites (Table 1), tubulin polymerization-promoting protein (TPPP) and Ermin are specific to oligodendrocytes (Brockschnieder et al., 2006; Song et al., 2007), and the other 11 proteins are potential neuronal targets, including EPS8, an actin capping protein; TAO1 and TAO2, microtubule and actin interactor binding proteins; FMNL2, an actin binding protein, Arhgap8, a Rho-GTPase activator; and GIPC and Tsg101 that function in vesicle trafficking. Another candidate MST3 substrate is Caspase 3 involved in the elimination of synapses in neurons (D’Amelio et al., 2012).

This study further uncovered a phosphorylation consensus sequence for MST3: threonine (as opposed to serine) is the phosphorylated amino acid followed by Isoleucine, Leucine, or Valine. In about half of the substrates that we identified, an Arginine was found at +2, resulting in a consensus site of T^∗^− I/L/V− R for phosphorylation by MST3.

### Thousand and One Amino Acid1/2 Phosphorylation by MST3 Is Important for Limiting Filopodia and Maintaining Spines

**Figure 4:**
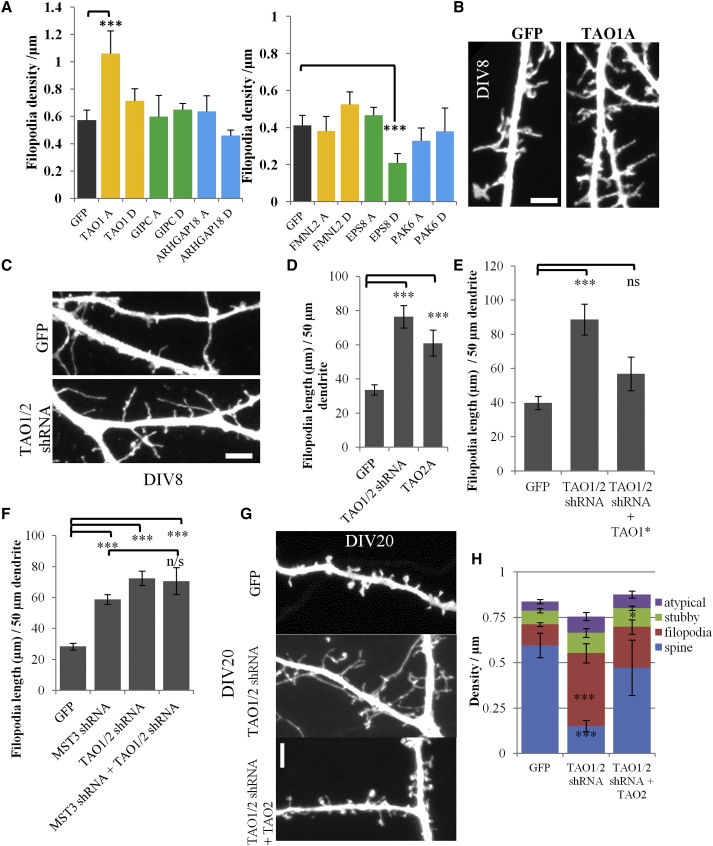
Effects of Phosphomutant and Phosphomimetic Substrate Expression on Dendritic Filopodia in Hippocampal Neurons (A) There were six putative MST3 substrates TAO1, GIPC, ArhGAP18, FMNL2, EPS8, and PAK6 that were mutated at their MST3 phosphorylation site to produce phosphomutant (T > A, e.g., TAO1A) or phosphomimetic (T > D, e.g., TAO1D) mutants. Filopodia density is measured in neurons transfected at DIV3 and imaged at DIV7 (For left graph n = 6, 6, 6, 6, 5, 4, and 5 cells, for right graph n = 6, 8, 6, 8, 9, 5, and 5 cells, respectively). (B) Representative examples of dendrites of neurons expressing GFP alone and GFP and TAO1A mutant. Scale bar represents 2.5 μm. (C) Representative images of dendrite expressing GFP alone and GFP and TAO1/2 shRNA. Scale bar represents 2.5 μm. (D) TAO1/2 shRNA and TAO2A significantly increases filopodia length per 50 μm of dendrite (n = 14, 14, and 6 cells, respectively). (E) Increase in filopodia by TAO1/2 shRNA can be rescued by TAO1^∗^ resistant to TAO1/2 shRNA (n = 7, 9, and 9 cells, respectively). (F) Total filopodia length per 50 μm dendrite is increased in MST3 shRNA and TAO1/2 shRNA expressing neurons. MST3 and TAO1/2 shRNA knockdown together does not further increase filopodia (n = 5, 7, 8, and 8 cells, respectively). (G) Images of dendritic spines of neurons transfected with GFP, TAO1/2 shRNA, and TAO1/2 shRNA cotransfected with human TAO2 construct, which is insensitive to this shRNA, are shown. Scale bar represents 3 μm. (H) Dendritic filopodia density is increased and spine density is reduced significantly upon TAO1/2 knockdown. The effect was mitigated by expression of human TAO2 together with TAO1/2 shRNA (n = 10, 10, and 9 cells, respectively). All error bars reflect SEM. See also Figure S4.

To find out whether some of the putative MST3 substrates modulate filopodia and spine morphogenesis, we tested phospho mutants and phospho-mimetic mutants of TAO1, GIPC, ARHGAP18, FMNL2, EPS8, and PAK6 by expressing HA tagged mutant substrates in hippocampal neurons at DIV3, confirming their expression by HA immunostaining (Figure S4A) and assessing filopodia phenotypes at DIV7–8. Of the six substrates tested, the EPS8 phospho-mimetic form (T317D) caused reduction of filopodia density, while the TAO1 phospho mutant (T440A or TAO1A) increased filopodia density per dendrite length and total filopodia length per 50 μm dendrite, similar to the loss of function of MST3 phenotype (Figures 4A, 4B, and S4B). The TAO1 T440D mutant (TAO1D) or wild-type TAO1 expression did not alter filopodia structure or density (Figures 4A, S4B, and S4C). While this experiment does not rule out roles of other identified downstream effectors, it shows that TAO1 and TAO2 kinase phosphorylation by MST3 is critical for limiting dendritic filopodia.

Next, we tested knockdown of TAO1 or EPS8 by generating three shRNAs in each case. Expressing shRNAs targeting TAO1 alone (not TAO2) or EPS8 between DIV3 and DIV7–8 did not alter filopodia of hippocampal neurons (Figures S4D and S4E, and data not shown), notwithstanding the ability of EPS8 to limit filopodia (Menna et al., 2013; Stamatakou et al., 2013) and the effect of the EPS8 phospho-mimetic form in reducing filopodia density (Figure 4A). It is conceivable that the EPS8 function could be compensated for by other isoforms such as EPS8L2 and EPS8L3 that also contain the MST3 phosphorylation site.

A TAO2 targeting shRNA with a one base pair mismatch with TAO1 knocked down TAO2 together with TAO1 so is referred to as TAO1/2 shRNA (Figure S4F). Transfection at DIV3 with TAO1/2 shRNA or TAO2 phospho mutant (T475A) caused a robust increase at DIV7–8 in dendritic filopodia, similar to the MST3 loss of function phenotype (Figures 4C, 4D, and S4G), and this phenotype can be rescued with shRNA-resistant mouse TAO1^∗^ (Figure 4E). There were two additional shRNAs targeting TAO2 only that did not yield this phenotype (data not shown). Expression of both MST3 shRNA and TAO1/2 shRNA increased the total filopodial length per 50 μm dendrite to a similar extent as MST3 knockdown (Figure 4F), indicating that MST3 and TAO1/2 are acting on the same pathway.

To test for TAO1/2 function in the spine maintenance, we transfected cultured neurons with TAO1/2 shRNA at DIV16 and imaged these neurons at DIV20. TAO1/2 shRNA caused a reduction in dendritic spines and an increase in filopodia, which was rescued by shRNA-resistant human TAO2 (Figures 4G and 4H). Expression of PSD-95-GFP with Td-tomato and TAO1/2 shRNA between DIV13 and DIV16 revealed a reduction in the density of protrusions containing PSD-95-GFP (Figures S4H and S4I), implicating TAO1/2 in spine synapse maintenance as well as filopodia development.

### MST3 Is Necessary and Sufficient for Thousand and One Amino Acid1/2 T440/T475 Phosphorylation

**Figure 5:**
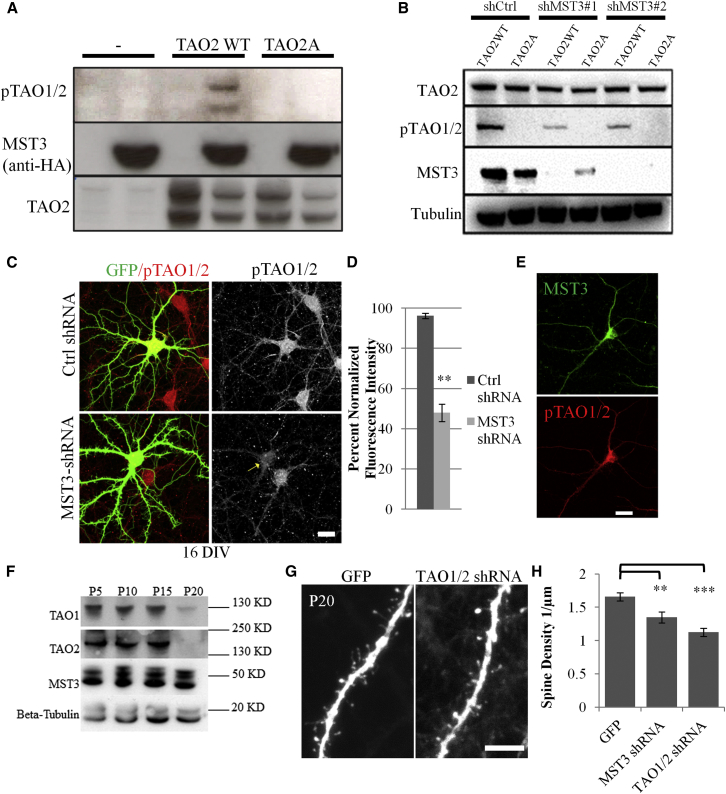
MST3 Is Necessary and Sufficient to Phosphorylate Thousand and One Amino Acid1/2 at T440/475 (A) pTAO1/2 signal is increased when MST3-HA is coexpressed with wild-type TAO2, but not with TAO2-A (T475A). Expressed TAO2 migrates at two separate bands at ∼140 kDa and ∼200 kDa in this experiment, although the expected size is 140 kDa. (B) TAO2 WT or TAO2A is transfected in HEK293T cells together with MST3 shRNAs targeting human MST3 or a control shRNA. TAO2 phosphorylation at T475 is reduced when MST3 is knocked down in lanes three and five when compared to control in lane one, suggesting that MST3 is necessary for T475 phosphorylation. As expected, pTAO1/2 antibody does not detect any signal when TAO2A is expressed in lanes two, four, and six. (C) MST3 shRNA causes reduction of phosphorylated TAO1/2 440/475. Hippocampal neurons expressing control shRNA or MST3shRNA with GFP are stained with phosphoTAO1/2 antibody (red). Scale bar represents 20 μm. (D) Quantification of phosphorylated TAO1/2 in cell body is significantly reduced in MST3 shRNA (n = 3 experiments each). (E) MST3 and phosphoTAO1/2 440/475 costaining is shown. Scale bar represents 20 μm. (F) Expression of TAO1, TAO2, and MST3 in brain lysates during development. (G) TAO1/2 shRNA expressing layer 2/3 neuron basal dendrites in vivo (via in utero electroporation) at P20 is shown. Scale bar represents 5 μm. (H) Quantification of spine density in neurons expressing GFP, MST3 shRNA, and TAO1/2 shRNA (n = 4, 3, and 3 animals and 19, 14, and 13 neurons, respectively). All error bars reflect SEM. See also Figure S5.

We raised a phospho-specific antibody (pTAO1/2) against the phosphorylation site T440/T475 on TAO1/TAO2, which is almost identical in TAO1 and TAO2, cotransfected MST3-HA with wild-type TAO2 or T475A mutant TAO2 (TAO2A), and found MST3-dependent phosphorylation of the wild-type, but not mutant TAO2 (Figure 5A). To test if MST3 is necessary for TAO2 phosphorylation, we transfected human embryonic kidney 293 (HEK293) cells with shRNAs targeting human MST3 along with wild-type TAO2 or TAO2 T475A mutant. MST3 knockdown reduced the levels of baseline phosphoTAO1/2, but not total TAO2 protein (Figure 5B). Similar results were obtained for TAO1 (data not shown), indicating MST3 is necessary and sufficient for TAO1/2 phosphorylation.

Next, we transfected hippocampal neurons at DIV13 with control shRNA or MST3 shRNA and stained these neurons at DIV16 with phosphoTAO1/2 antibody, after verifying its specificity by showing reduced immunostaining upon TAO1/2 shRNA expression (Figure S5A). MST3 knockdown reduced phosphoTAO1/2 levels in the soma (Figures 5C and 5D). Immunostaining further indicated that MST3 phosphorylation of TAO1/2 could take place in the cell body and/or dendrites (Figure 5E).

Thousand and one amino acid1/2 (TAO1/2) kinases are Ste20-like kinases implicated in microtubule and actin regulation (see Discussion). TAO1 and TAO2 are expressed from postnatal day (P)5 to P15 and then downregulated at P20, although TAO1 expression was still detectable (Figure 5F). We next investigated their role in spine development in vivo by performing in utero electroporations at embryonic day (E)14.5 either with GFP plasmid alone as control or together with MST3 shRNA or TAO1/2 shRNA, and imaging dendritic spines of perfusion fixed brains at P20. Similar to MST3 shRNA, TAO1/2 shRNA caused reduction in dendritic spine density in vivo (Figures 5G and 5H).

### A Myosin-Va Containing Complex Binds Thousand and One Amino Acid1/2 Phosphorylation Site in a Phosphorylation Dependent Manner

To test if the phosphorylated TAO1/2 site is involved in phosphorylation–dependent protein interaction, we used a peptide pull-down method to identify binding proteins in neuronal lysates labeled by stable isotope labeling by amino acids in culture (SILAC) (Spellman et al., 2008; Stephanowitz et al., 2012; Zhang et al., 2011).

**Figure 6:**
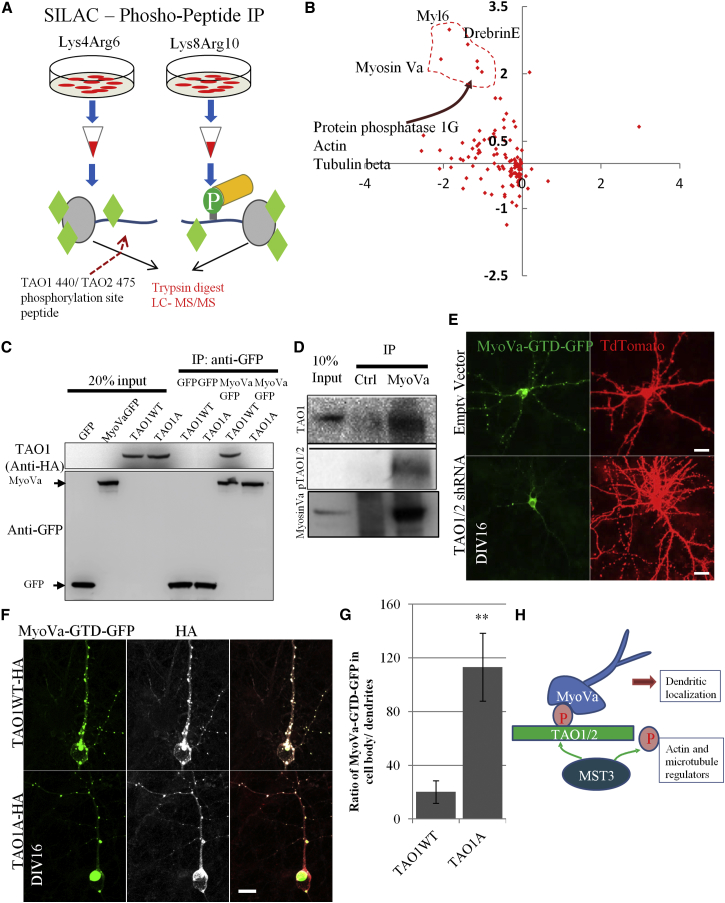
Myosin Va Interacts with Thousand and One Amino Acid1/2 upon Its Phosphorylation by MST3 (A) Schematic representation of SILAC experiment with peptide pull-down. Hippocampal neurons were cultured in SILAC media containing either medium heavy (Lys4 Arg6) or heavy (Lys8 Arg10) amino acids from plating to DIV9 when they were harvested. Protein lysates were incubated with beads attached with 17 amino acids long peptides containing TAO1/2′s MST3 phosphorylation site, either in phosphorylated and nonphosphorylated forms. Proteins bound to the peptides were digested and identified in MS. (B) There were two separate experiments where SILAC labels that were switched are plotted with respect to each other. Higher values in positive y axes and negative x axes represent enriched binding of proteins to phospho-peptide. The proteins enriched at values >1 in both experiments are circled by a dotted line. Myosin Va was enriched >2 in both experiments (see Table S1 for all values). (C) GFP immunoprecipitation of Myosin Va-GTD-GFP, labeled as Myosin Va-GFP, after incubation with lysates expressing TAO1-HA wild-type and TAO1-HA T440A mutants, reveal that only TAO1WT and not TAO1A can bind (coimmunoprecipitate) Myosin Va-GTD-GFP. (D) Endogeneous Myosin Va is immunoprecipitated using an antibody from dissociated hippocampal neurons protein lysates (at DIV6 or DIV9). Immunoblots show that endogeneous TAO1 and phosphoTAO1/2 are immunoprecipitated by endogeneous Myosin Va. (E) MyosinVa-GTD-GFP distribution in dendrites is altered upon TAO1/2 shRNA expression in cortical neurons. Myosin Va-GTD-GFP is imaged by confocal microscopy without amplification via immunostaining. Z projections are shown. Td-tomato is expressed pLentiLox3.7 (empty vector) in which GFP is replaced by Td-tomato. TAO1/2 shRNA is also expressed on the same plasmid at a different promoter. Images are captured with same settings. N = 8 out of 8 neurons expressing empty vector showed the showed punctate Myosin Va-GTD-GFP in dendritic arbor. Neurons expressing TAO1/2 shRNA lacked dendritic distribution as depicted in lower panel n = 9 out of 10. Scale bars represent 25 μm. (F) Myosin Va-GTD-GFP is coexpressed with either wild-type or T440A mutant TAO1-HA in cortical neurons. TAO1 (HA staining) is colocalized with Myosin Va-GTD-GFP (arrows). Scale bar represents 10 μm. (G) Myosin Va-GTD-GFP accumulation in cell body when compared to dendritic Myosin Va was lower in TAO1-WT expressing neurons than in TAO1-A expressing neurons. Total cell body Myosin Va-GTD-GFP intensity is measured in z projected images and normalized per area after background subtraction. Dendritic puncta were outlined individually, total intensity was measured and normalized to total puncta area and dendritic length (∼30 μm dendrite was included). Ratio between cell body/dendritic Myosin Va intensity is shown for TAO1-wt and TAO1-A (n = 7 and 8 neurons, respectively) (p < 0.01). (H) Signaling pathway identified. MST3 phosphorylates several substrates particularly actin and microtubule binding/regulating proteins. MST3 phosphorylates TAO1/2, upon phosphorylation Myosin Va containing complex can bind TAO1/2. TAO1/2 function is critical for Myosin Va localization to dendrites. All error bars reflect SEM. See also Figure S6.

We incubated iodoacetyl agarose beads conjugated with peptides corresponding to the TAO2-T475 phosphorylation site in either nonphosphorylated or phosphorylated forms (Figure 6A) with neuronal lysates labeled with medium heavy (Lys4 Arg6) and heavy (Lys8 Arg10) amino acids, and subjected the proteins bound to the peptide-coated beads to trypsin digest and mass spectrometry. The relative abundance of each protein was calculated based on the median of the medium heavy/heavy ratios for all peptides identified for that protein. A majority of proteins associated with both peptide- and phospho-peptide conjugated beads (diamonds in Figure 6A), whereas a number of proteins were enriched in the phospho-peptide conjugated beads. We repeated this experiment by switching the medium heavy and heavy labels on peptide/phospho-peptide pull-downs and plotted results from the first and second experiments on the x and y axis. The numbers represent the base 2 logarithms of the ratio of medium heavy/heavy forms of each detected protein (Figure 6B), so that the top left quadrant contains proteins that are enriched in phospho-peptide pull-down in both experiments (Table S1). We found that Myosin Va, Myosin light chain 6, and DrebrinE bound to the phospho-peptide with higher efficiency than nonphosphorylated peptide (Figure 6B).

To verify the phospho-specific binding between the TAO1/2 T440/T475 site and Myosin Va, we incubated lysates from HEK293 cells expressing GFP alone or GFP tagged Myosin Va-globular tail domain (GTD) with lysates from cells expressing either TAO1-HA or TAO1-T440A-HA. Immunoprecipitation using an anti-GFP antibody followed with western blot of the bound proteins with HA antibody revealed that TAO1-HA, but not TAO1-T440A-HA could bind Myosin Va (Figure 6C). Similar coimmunoprecipitation experiments using Drebrin-GFP did not confirm binding between TAO1-HA and Drebrin-GFP (data not shown). To test if endogenous TAO1 or TAO2 can bind endogenous Myosin Va, we immunoprecipitated Myosin Va from dissociated hippocampal neurons at DIV6 and found that Myosin Va can bind TAO1 and phosphoTAO1/2 specifically (Figure 6D), while we were not able to detect a signal with TAO2 antibody (data not shown). Thus, MST3 phosphorylates TAO1 and TAO2, enabling their association with Myosin Va.

### Myosin Va Recruitment to Dendrites Is Enhanced by Thousand and One Amino Acid1/2

Myosin V has been implicated in spine morphology and α-Amino-3-hydroxy-5-methyl-4-isoxazolepropionic acid (AMPA) type glutamate receptor trafficking (Correia et al., 2008; Lisé et al., 2006; Wang et al., 2008). To test for TAO1/2 involvement in Myosin Va function, we transfected cortical neurons at DIV13 with GFP tagged Myosin Va-GTD, a second plasmid (pLentiLox 3.7) coexpressing membrane tagged Td-tomato to visualize neuronal membrane, and shRNA targeting TAO1/2, and imaged at DIV16 after fixation. Myosin Va-GTD-GFP accumulated in the dendrites in punctate pattern (Figure 6E, top row, n = 8 neurons). Upon TAO1/2 shRNA expression, the dendritic Myosin Va-GTD-GFP distribution was disrupted in nine out of ten neurons (Figure 6E, bottom row) (p < 0.001 chi-square). Myosin Va-GTD-GFP can still be localized to axons in TAO1/2 shRNA neurons (Figure S6F), indicating that dendritic localization is specifically altered in the absence of TAO1/2.

To test if TAO1/2 knockdown affects specifically Myosin Va, we transfected cortical neurons with HA tagged Myosin Va-GTD or HA tagged Myosin Vb-GTD together with vector expressing GFP alone or GFP with TAO1/2 shRNA (Figure S6), and immunostained these neurons with an anti-HA antibody (red). Myosin Va-HA was found in dendrites in eight out of ten neurons coexpressing GFP, whereas TAO1/2 knockdown in all 11 neurons reduced Myosin Va in dendrites (Figures S6A and S6C, p < 0.001 chi-square). Unlike Myosin Va, Myosin Vb-GTD puncta accumulated in the perinuclear region of control neurons (Figure S6B, arrow, n = 12 neurons). Interestingly, TAO1/2 knockdown caused Myosin Vb-HA puncta to appear in dendrites in 12 out of 13 neurons (Figures S6B and S6D, p < 0.001 chi-square). Thus, TAO1/2 facilitates dendritic localization of Myosin Va, and knockdown of TAO1/2 results in an increase of Myosin Vb in dendrites, possibly via a compensatory upregulation. Moreover, Myosin Va-GTD-GFP puncta colocalized with endogenous Rab11 (Figure S6E), indicating that Myosin Va could be recruited to recycling endosomes, where its function could be required for dendritic spine formation.

To find out if the T440 residue of TAO1 is important for the dendritic localization of Myosin Va-GTD-GFP, we expressed TAO1wild-type (WT) or TAO1 T440A mutant together with Myosin Va-GTD-GFP in dissociated cortical neurons at DIV13 and imaged at DIV16. TAO1WT and TAO1A colocalized with Myosin Va-GTD-GFP (Figure 6F), in contrast to the diffuse cytoplasmic staining in GFP expressing control neurons (Figure S4A). By taking the ratio of the normalized Myosin Va-GTD-GFP signal in the cell body and that in a primary dendrite, we found that Myosin Va-GTD-GFP is enriched in the cell body in TAO1A expressing neurons when compared to TAO1WT expressing neurons (Figure 6G, n = 7 and 8 neurons in TAO1WT and TAO1A, respectively). Thus, while there might be other proteins that enable Myosin Va and TAO1 to be found in the same complex, T440 phosphorylation is critical in enabling dendritic localization of a larger percentage of Myosin Va. In summary, not only does Myosin Va associate with TAO1/2 kinase, but its localization is also altered by TAO1/2 function and phosphorylation state, which in turn is dependent on MST3 kinase, as depicted in Figure 6H.

## Discussion

In this study, we examine MST3 kinase signaling by identifying multiple potential substrates, mostly cytoskeletal regulators. In particular, the TAO1/2 kinase upon phosphorylation by MST3 on site T440/T475 associates with Myosin Va in a phosphorylation dependent manner, and this phosphorylation promotes Myosin Va recruitment in dendrites. Thus by using chemical genetic techniques, we have uncovered a kinase signaling cascade that functions in neuronal dendritic spine and synapse formation.

### Role of MST3 in Dendrite, Dendritic Filopodia, and Spine Development In Vitro and In Vivo

By expressing MST3 shRNA or KD MST3 in cultured hippocampal neurons, we show MST3 is required for limiting dendritic filopodia and maintaining dendritic spine and excitatory synapse structure. Whereas the MST3 shRNAs used in our study target regions common to MST3a and MST3b, expression of the shorter MST3a isoform is sufficient for rescuing the shRNA effects. MST3b has distinct functions in axonal growth, which could be due to possible specific subcellular localization or interactions. Using pan-MST3 antibody raised against a common epitope of MST3a and MST3b, we observed a mostly dendritic and perinuclear staining for MST3 (Figure 1), which overlaps with phosphoTAO1/2 staining (Figure 5E) and supports their function for dendritic filopodia and spine development. MST3 is also implicated in radial neuronal migration (Tang et al., 2014). Our in vivo analyses using in utero electroporations at E14.5 showed a majority of electroporated neurons in upper layers at P20. Hence, we focused on spine development on layer 2/3 neuron basal dendrites.

#### MST3′s Phosphorylation Substrates

Notwithstanding its broad expression pattern and its functions in apoptosis and axonal growth, little is known about the phosphorylation targets of MST3 except for NDR1 kinase (Stegert et al., 2005). By using the covalent capture method as an unbiased screen that can use complex protein mixtures as the source of substrates and can also identify the phosphorylation site (Hertz et al., 2010; Ultanir et al., 2012), we identified 13 phosphorylation targets of MST3, as well as a consensus motif for MST3 phosphorylation.

Several of the candidate MST3 substrates identified in this study implicate MST3 in regulating the cytoskeleton (Table 1). Among these candidates, we chose TAO1/2 for further study (see next section), and the rest are briefly discussed here. EPS8 is an actin capping protein (Menna et al., 2009) originally identified as a phosphorylation target of the epidermal growth factor receptor. Interestingly, EPS8 positively regulates spine formation and maturation and negatively regulates filopodia (Menna et al., 2013; Stamatakou et al., 2013), in agreement with our finding that the EPS8 phospho-mimetic form reduces filopodia formation. The lack of effect of EPS8 shRNA in hippocampal neuronal cultures could be due to the potential presence of its homologs EPS8L, EPS8L2, and EPS8L3. Several potential MST3 substrates could contribute to the regulation of dendritic actin cytoskeleton: Arhgap18 is a Rho-GTPase activating protein (GAP) (Maeda et al., 2011), PAK6 is an effector of the Rho family GTPases, and Formin-like protein 2 (FMNL2) is an actin binding protein that can bind and bundle actin filaments (Schönichen and Geyer, 2010). In addition, we identified two oligodendrocyte-specific cytoskeleton regulators, namely TPPP/p25alpha (Song et al., 2007) and Ermin (an actin binding protein) (Brockschnieder et al., 2006), as putative MST3 substrates, further implicating MST3 in cytoskeletal regulation in oligodendrocytes.

There were two of the putative substrates, GIPC and Tsg101, that suggest a role for MST3 in the regulation of membrane trafficking. GAIP interacting protein, C terminus (GIPC) is a PDZ domain containing protein, which regulates trafficking of membrane proteins including *N*-methyl-D-aspartate (NMDA) receptors in neurons (Yi et al., 2007). Tumor susceptibility gene 101 (Tsg101) is a component of the endosomal sorting complex, required for ubiquitin-dependent sorting of proteins into endosomes.

Identification of Caspase 3 as a putative MST3 substrate is intriguing, as Caspase 3 is activated by MST3 in human trophoblasts (Wu et al., 2008, 2011). Whereas the MST3 phosphorylation site is not conserved in human Caspase 3, a potential phosphorylation site (T16) is found in a similar location in human Caspase 6-beta. An interesting hypothesis is that this activation might arise from direct phosphorylation of Caspase 3 or Caspase 6 by MST3 in mice and humans, respectively.

Cystine glutamate transporter/Slc7a11, a transporter associated with glutamate toxicity in neurons, a putative substrate of MST3, is active in both neurons and glia (Bridges et al., 2012; Dixon et al., 2012; Jackman et al., 2012). Finally, the identification of eukaryotic elongation factor 1a as a putative MST3 target has implications in translational regulation.

Spine morphogenesis is regulated by NDR1/2 (Ultanir et al., 2012). Notably, although both NDR1/2 and MST3 kinases alter spine morphogenesis, they do so via distinct sets of putative substrates identified by our chemical genetic screen. NDR1/2′s substrates implicate membrane trafficking regulation whereas MST3′s substrates implicate cytoskeleton regulation.

#### Roles of the Phosphorylation Targets of Thousand and One Amino Acid 1 and Thousand and One Amino Acid 2 Kinases in Dendritic Filopodia and Spine Development

Expression of phospho mutant and phospho-mimetic mutant substrates could act as dominant-negative and constitutively active forms of the effectors, thereby resulting in phenotypes similar to the loss or gain of function phenotypes of MST3. Among the tested substrates, we found that TAO1 and TAO2 phospho mutants resulted in phenotypes strikingly similar to that of MST3 loss of function. We do not rule out the involvement of any of the other putative substrates, as not all phospho mutants may act as dominant negatives. Our screen, however, was useful in identifying TAO1/2 kinases as likely downstream effectors for more in depth investigation. Using shRNA expression, we demonstrated that TAO1/2, and in particular the TAO2 activity, is required for dendritic filopodia development and maintenance of spines. We further confirmed that MST3 is necessary and sufficient to phosphorylate TAO1/2.

TAO1 and its homolog TAO2 are serine/threonine kinases in the Ste20 kinase family. TAO1 is also called microtubule affinity regulating kinase kinase, and it regulates microtubule affinity regulating kinase (MARK/Par1) via phosphorylation (Timm et al., 2003). MARK, in turn, phosphorylates MAPs to cause microtubule breakdown (Drewes et al., 1997). Implicated in behaviors induced by ethanol, nicotine, and cocaine, *Drosophila* TAO genetically interacts with the microtubule regulator Par1 and is required for the development of the mushroom body in the *Drosophila* brain (King et al., 2011). TAO1 colocalizes with microtubules in S2 cells and limits microtubule growth (Liu et al., 2010). TAO1 may also form a link between microtubules and actin cytoskeleton by interacting with actin and microtubule binding proteins (Johne et al., 2008; Mitsopoulos et al., 2003). It remains to be determined how TAO1 functions in cytoskeleton regulation and whether its functions depend on MST3 phosphorylation.

TAO2 is highly similar to TAO1 in its kinase domain. TAO1 and TAO2 are expressed in the brain (Hutchison et al., 1998) and are involved in p38 MAP kinase regulation (Chen et al., 1999; Hutchison et al., 1998). Interestingly, TAO2 has also been implicated as a candidate gene for the autism spectrum disorder (Weiss et al., 2008), a neurodevelopmental disorder. TAO2 is localized to actin cytoskeleton in dendritic growth cones in the first postnatal week and is involved in basal dendrite development in cortical neurons via its interaction with the semaphorin 3A receptor Neuropilin and subsequent c-Jun N-terminal kinase activation (de Anda et al., 2012). Arcadlin, an activity-regulated cell adhesion molecule, can bind N-cadherin and a shorter splice isoform of TAO2, TAO2b, to activate p38 MAP kinase, resulting in N-cadherin internalization (Yasuda et al., 2007). Actin and microtubule often interact in cytoskeleton. Of particular interest is the regulation of Myosin Va’s association in dendritic cytoskeleton as described below.

#### Thousand and One Amino Acid 1/2 Binding and Regulation of Myosin V Motors

Phosphorylation is a posttranslational modification that may alter protein function by affecting its interaction with binding partners. The observation that the TAO1A and TAO2A phospho mutant expression increases filopodia density underscores the significance of MST3 phosphorylation. We hypothesize that these mutant kinases might be binding and titrating components of TAO1/2 signaling, thus acting as a dominant negative.

Using a SILAC based immunopreciptation method, we identified a protein complex including Myosin Va, Myosin light chain 6, and DrebrinE that bind peptides bearing the TAO1/2 phosphorylation site in a phosphorylation dependent manner. Unlike TAO1WT, TAO1A is unable to bind Myosin Va. We further confirmed the phosphospecific binding of Myosin Va to TAO1/2 via coimmunoprecipitation. Moreover, endogenous Myosin Va can bind endogenous TAO1 and phosphorylated TAO1/2 in neurons.

Myosin Va is an abundant protein in the brain and is present in synaptic fractions (Walikonis et al., 2000). An unconventional actin motor protein, Myosin Va in vertebrates can tether cargo to actin cytoskeleton at the cell periphery (Wu et al., 1998); reduce microtubule based transport when recruited on the same cargo with the microtubule motor kinesin (Kapitein et al., 2013); and perform short-range transport of cargo on actin filaments (Hammer and Sellers, 2012; Kapitein et al., 2013). In hippocampal neurons, both Myosin Va (Correia et al., 2008) and Myosin Vb (Lisé et al., 2006; Wang et al., 2008) are implicated in trafficking of AMPA type glutamate receptors into dendritic spines, although previous studies showed no defects in basal or plasticity induced AMPA receptor recruitment to synapses in Myosin Va mutant mice Dilute-Lethal (Petralia et al., 2001; Schnell and Nicoll, 2001). Myosin Va is also implicated in transporting endoplasmic reticulum in Purkinje neuron dendritic spines (Wagner et al., 2011). Myosin Va knockdown in neuronal cultures drastically reduces spine density (Lisé et al., 2009). A recent examination of the Myosin Va neurological mutant mice flailer, which express a dominant-negative Myosin Va fusion protein lacking the actin binding domain, revealed a reduction of mature dendritic spines and an increase in dendritic filopodia (Yoshii et al., 2013). In these mutant mice, the mature excitatory synaptic markers PSD-95, stargazin, the endocytic zone marker Dynamin 3, and AMPA receptors are drastically mislocalized to dendritic shaft (Yoshii et al., 2013). These data support the notion that Myosin Va is critical for mature spine synapse formation. Interestingly, despite the loss of mature spine synapses in flailer mice, the AMPA receptor currents are increased (Yoshii et al., 2013). Our observation of increased Myosin Vb trafficking to dendrites in the absence of TAO1/2 and dendritic Myosin Va is indicative of a compensatory Myosin Vb function in the absence of Myosin Va.

We find that Myosin Va-GTD is localized in a punctate pattern along the dendrites and colocalizes with the recycling endosome marker Rab11, and we show that this recruitment is dependent on TAO1/2 function. Membrane transport from recycling endosomes is required for maintenance of dendritic spine structure as well as for delivery of AMPA receptors during synaptic plasticity (Park et al., 2006). TAO1 colocalizes with Myosin Va-GTD independent of its T440 phosphorylation, implicating a mechanism for recruitment of TAO1 into Myosin Va complexes that is distinct from their interaction via the T440 region. Interestingly, TAO1A shifts Myosin Va distribution from dendrites to neuronal cell body, suggesting that the T440 site on TAO1 is important in regulating TAO1 function and dendritic localization of Myosin Va in controlling spine development.

The opposite effects of TAO1/2 shRNA knockdown on the expression of Myosin Va and Myosin Vb could reflect a homeostatic response to the reduction of Myosin Va. How TAO1/2 regulates Myosin Va remains an open question. A possibility is that TAO1/2 regulates Myosin V motor function by binding and phosphorylating components of the Myosin Va complex, and enhancing its dendritic localization.

#### Chemical Genetics for Kinase Substrate Identification

A chemical genetic screen for kinase substrate identification is an unbiased method to identify substrates in relatively intact protein lysates without the need for peptide arrays or gel electrophoresis. The combination of simple chemistry to highly enrich for peptides of substrates phosphorylated by a kinase of interest, and the ability to identify the substrates as well as the phosphorylation sites by using powerful liquid chromatography/tandem mass spectrometry renders this technique uniquely advantageous for studying downstream effectors of kinases.

Phosphorylation often alters protein function by changing binding properties of substrates. We have now added a further technical advance to the kinase substrate identification screen through which we can determine the phosphorylation state dependent protein interactors that bind the substrates on the phosphorylation sites. Using a quantitative, unbiased peptide pull-down screen approach, which can be achieved by SILAC labeling of proteins in neuronal cultures, we were able to identify protein interactors of TAO1/2 that critically depend on their MST3 phosphorylation site. We believe our methodology of chemical genetic kinase substrate identification, followed by determining the functionality of the phosphorylation is a useful paradigm in delineating kinase signaling.

## Experimental Procedures

### Neuronal Cultures, DNA Constructs, Small Hairpin RNA, and Lentiviruses

Hippocampal neurons were cultured from E19 Long-Evans rats (Charles River Lab) in accordance with local guidelines at the University of California San Francisco (UCSF) and National Institute for Medical Research as described before (Ultanir et al., 2012). Mouse MST3 (BC004650) cDNA purchased from ATCC was cloned in pRK5 mammalian expression vector with N-terminal HA tag. Human full-length TAO2 (1,235 amino acids) in pCMV Sport 6.0 expression vector was a gift from Dr. Froylan Calderon De Anda and Dr. Li-Huei Tsai from the Massachusetts Institute of Technology (de Anda et al., 2012). Human FMNL2, human ArhGAP18, mouse GIPC, mouse EPS8, human TAO1, and human PAK6 were purchased from Thermo Scientific and cloned into pRK5 vector with N-terminal HA tag. Mutations were generated by site directed mutagenesis. Myosin Va-GTD-GFP, Myosin Va-GTD-HA, and Myosin Vb-GTD-HA vectors were gifts from Dr. Don Arnold from the University of South California (Lewis et al., 2009).

All shRNA sequences were 19 base pairs long and were selected via http://katahdin.cshl.org/html/scripts/main.pl. Hairpins targeting these sequences were cloned in pLentiLox 3.7, which expresses EGFP via a separate promoter in addition to expressing shRNA via a U6 promoter. Lentiviruses were generated at UCSF core facilities. All clones were verified by sequencing.

### In Utero Electroporation

In utero electroporation surgery was performed in accordance with local guidelines as described (Ultanir et al., 2012). Embryos were injected with 1 μg/μl pCAG-GFP (control), or 0.4 μg/μl pCAG-GFP + 1 μg/μl MST3 shRNA #1, or 0.4 μg/μl pCAG-GFP + 1 μg/μl TAO1/2 shRNA. Mice were perfused at P18–P20 using 4% paraformaldehyde and 4% sucrose.

### Antibodies and Confocal Microscopy

For a detailed list of antibodies and dilutions, please see Supplemental Experimental Procedures. Dendrites were imaged using an inverted Leica SP5 confocal microscope using a 63× (NA 1.4) objective at 6× zoom. Z sections were obtained across the dendrite depth at 0.5 μm z intervals. Spine density and categorization was done manually using Leica image analysis. Spine head diameter measurements were done using a custom plugin in ImageJ (Ultanir et al., 2007).

### Mammalian Sterile 20-like Kinase 3 Kinase Assays

HA tagged MST3 was expressed in COS7 or HEK293T cells for 48 hr before purified via HA epitope tag using Anti-HA Affinity matrix (Roche, clone 3F10). Kinase assays were conducted in 20 mM Tris-HCl pH 7.5, 10 mM MgCl2, 1 mM dithiothreitol (DTT), 100 μM ATP, 1× Phosphatase inhibitor cocktail, and 0.5 mM of an ATP analog for 30 min at 30°C. Reaction was followed by alkylation for 1 hr at room temperature by addition of 2 μl 100 mM PNBM per 30 μl of kinase reaction.

### Mammalian Sterile 20-like Kinase 3 Substrate Labeling and Identification

Kinase substrate labeling was done in P3 and P13 mouse brain lysates obtained as described (Ultanir et al., 2012). Purified MST3-MG and MST3-KD were used for labeling. Covalent capture method for kinase substrate identification and mass spectrometry was done as previously described (Ultanir et al., 2012).

### Multiplex Stable Isotope Labeling by Amino Acids in Culture Labeling of Neuronal Cultures and Phospho-Peptide Pull-Down

Cortical neuronal cultures were labeled using Multiplex SILAC (Zhang et al., 2011), and peptide pull-downs using SILAC labeled lysates was conducted as described (Stephanowitz et al., 2012). TAO2 T475 peptide (C RNRDHFAT^∗^IRTASLVSR) was synthesized with or without a phosphorylation at the indicated threonine and conjugated to iodoacetyl agarose. Precipitated proteins were subjected to in-bead typsin digest followed by reversed-phase liquid chromatography-electrospray tandem mass spectrometry (LC-MS/MS) analysis.

### Immunoprecipitations

HEK293T cells transfected with Myosin Va-GTD-GFP, control GFP construct, HA-TAO1, and HA-TAO1A, separately. Lysate from Myosin Va-GFP or control GFP transfected cells was incubated with Protein G beads prebound with monoclonal GFP antibody, and then lysates from either HA-TAO1 or HA-TAO1A transfected cells were added and incubated with the beads containing Myosin V-GFP or GFP alone. For immunoprecipitation from neurons precleared lysates from DIV6 or DIV9 rat hippocampal neurons were incubated with Myosin Va antibody bound Protein A beads (Sigma) overnight at 4°C.

## Author Contributions

S.K.U. designed and performed spine and filopodia phenotype analysis in cultures and in vivo, MST3 substrate labeling, SILAC phosphopeptide pull-down, and wrote the manuscript with help from other authors. S.Y. designed and performed the immunoprecipitations, phosphoTAO1/2 staining, and contributed to immunostainings and biochemistry experiments on shRNA knockdown in neurons and HEK293 cells. N.T.H. designed and performed the covalent capture and MS for kinase substrate identification. J.A.O.-P. codesigned and performed MS for SILAC samples. S.C. contributed to neuronal cultures and cloned several constructs. A.L.B., K.M.S., L.Y.J., and Y.N.J. oversaw the project development.

## Figures and Tables

**Table 1:** Putative MST3 Substrates Are Listed

	Candidate	# /4 Exps	Phosphorylation Site		Function
1	EPS8	3	T317	PGE^a^GVLT^∗^L^a^R^a^	actin capping
2	TAO1	2	T440	NRE^a^HFAT^∗^I^a^R^a^	microtubule stability
3	TAO2	2	T475	NRE^a^HFAT^∗^I^a^R^a^	dendrite and spine development
4	FMNL2	2	T202	SALRYNT^∗^L^a^P	actin bundle formation
5	Arhgap18	3	T156	VETVSQT^∗^L^a^R^a^	actin regulation via Rho GTPase
6	Pak6	3	T99	SVISSNT^∗^I^a^R^a^	rhoGTPase effector
7	GIPC	2	T242	LGTGRGT^∗^L^a^R^a^	NMDA receptor trafficking
8	EF1A1	3	T432	VRD^a^MRQT^∗^V^a^A	translation elongation
9	Caspase-3	2	T20	NNFEVKT^∗^I^a^H	synaptic plasticity, dendrite pruning
10	Tsg101	3	T221	GPSRDGT^∗^I^a^S	membrane transport
11	Cystine glutamate transporter	3	T9	SAVSSPT^∗^V^a^	transporter
12	Ermin	2	T257	SYSRYNT^∗^I^a^S	actin regulation
13	TPPP	2	T161	RKPVVAT^∗^I^a^S	microtubule regulation

The number of times a protein is identified out of four MS experiments is shown in the third column. Phosphorylated amino acid number and the phosphorylation sites are shown in the fourth and fifth columns. Brief descriptions of reported functions of putative substrates are listed in the last column.

a Indicates the phosphorylation consensus motif that emerged from our screen.

## Supplemental Information

Document S1. Supplemental Experimental Procedures, Figures S1–S6, and Table S1

Document S2. Article plus Supplemental Information
