# Supplementary material for: MST3 Kinase Phosphorylates TAO1/2 to Enable Myosin Va Function in Promoting Spine Synapse Development
Source: Neuron. 2014 Dec 3;84(5):968–82. doi: 10.1016/j.neuron.2014.10.025 (PMC4407996; doi:10.1016/j.neuron.2014.10.025)
Supplement: Document S1. Supplemental Experimental Procedures, Figures S1–S6, and Table S1 [file mmc1.pdf]

Neuron, Volume *84*

Supplemental Information

MST3 Kinase Phosphorylates TAO1/2 to Enable Myosin Va Function in Promoting Spine Synapse Development

Sila K. Ultanir, Smita Yadav, Nicholas T. Hertz, Juan A. Oses-Prieto, Suzanne Claxton, Alma L. Burlingame, Kevan M. Shokat, Lily Y. Jan, and Yuh-Nung Jan

Inventory of Supplemental information

1. Supplemental Figure 1
2. Supplemental Figure 2
3. Supplemental Figure 3
4. Supplemental Figure 4
5. Supplemental Figure 5
6. Supplemental Figure 6
7. Supplemental Figure Legends
8. Supplemental Table 1 for Figure 6
9. Supplemental Experimental Procedures
10. Supplemental References

Figure S1

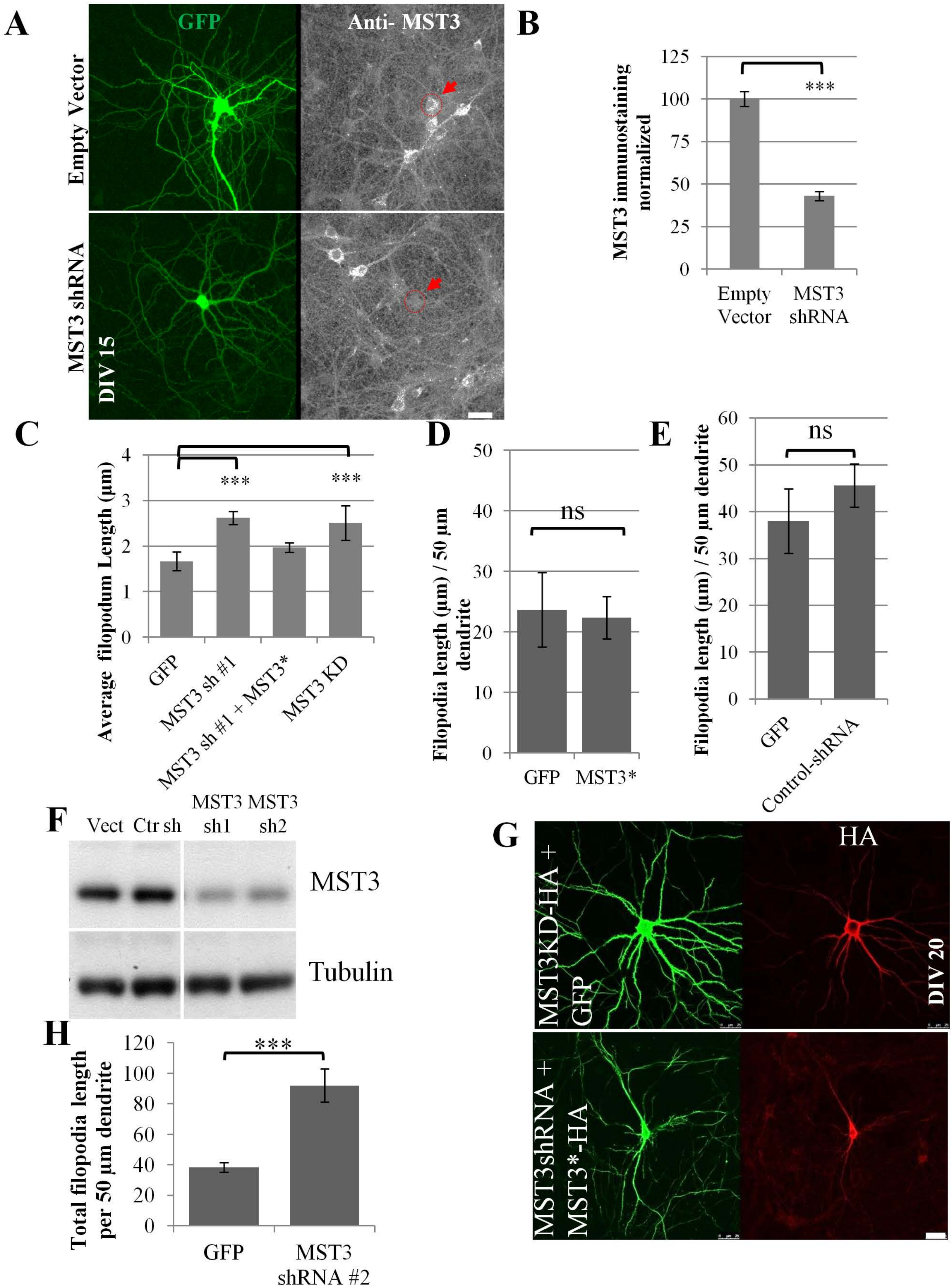
A
GFP
Anti- MST3
Empty Vector
MST3 shRNA
DIV 15
B
MST3 immunostaining normalized
125
100
75
50
25
0
***
Empty Vector
MST3 shRNA
C
Average filopodum Length (µm)
4
3
2
1
0
***
***
GFP
MST3 sh #1
MST3 sh #1 + MST3*
MST3 KD
D
Filopodia length (µm) / 50 µm dendrite
50
40
30
20
10
0
ns
GFP
MST3*
E
Filopodia length (µm) / 50 µm dendrite
60
50
40
30
20
10
0
ns
GFP
Control-shRNA
F
Vect
Ctr sh
MST3 sh1
MST3 sh2
MST3
Tubulin
G
HA
MST3KD-HA + GFP
DIV 20
MST3shRNA + MST3*-HA
H
Total filopodia length per 50 µm dendrite
120
100
80
60
40
20
0
***
GFP
MST3 shRNA #2

Figure S2

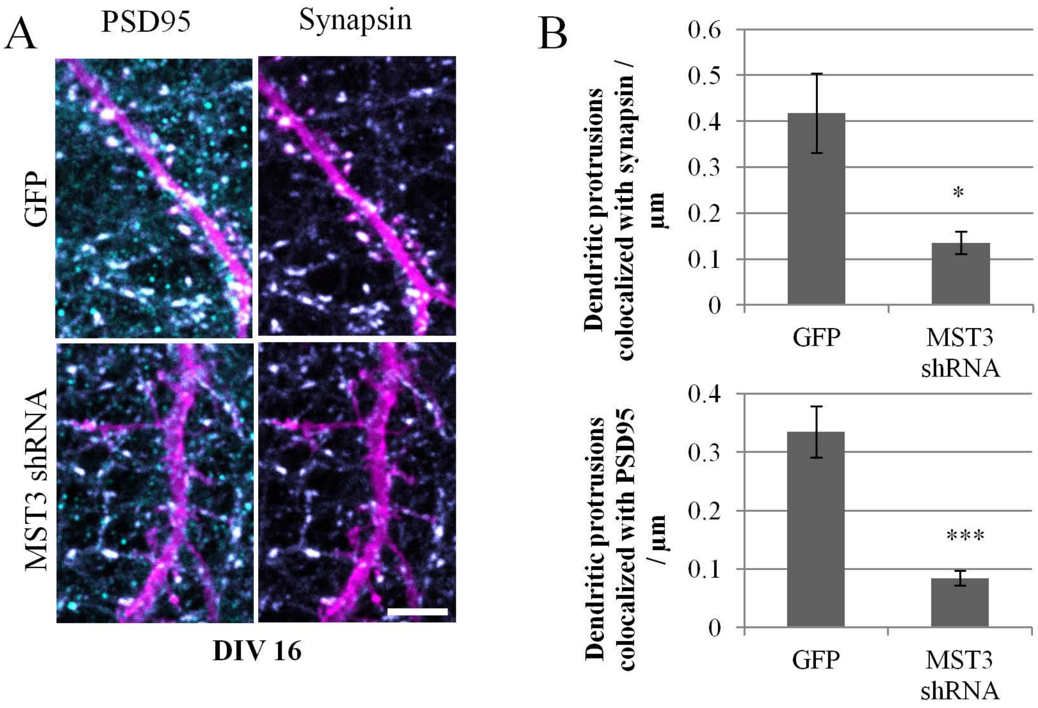

A
PSD95
Synapsin
GFP
MST3 shRNA
DIV 16
B
Dendritic protrusions colocalized with synapsin / µm
0.6
0.5
0.4
0.3
0.2
0.1
0
*
GFP
MST3 shRNA
Dendritic protrusions colocalized with PSD95 / µm
0.4
0.3
0.2
0.1
0
***
GFP
MST3 shRNA

Figure S3

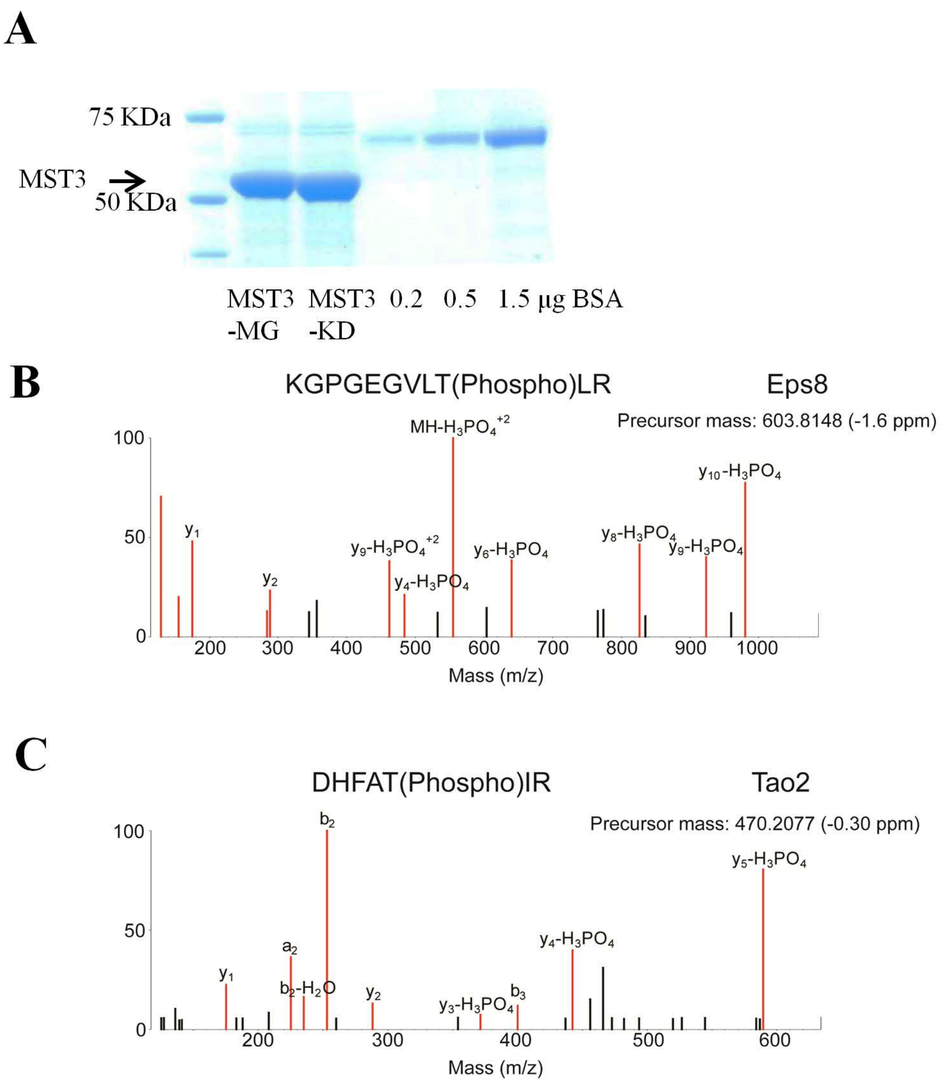
A
75 KDa
MST3
50 KDa
MST3 -MG
MST3 -KD
0.2
0.5
1.5 μg BSA
B
KGPGEGVLT(Phospho)LR
Eps8
Precursor mass: 603.8148 (-1.6 ppm)
MH-H3PO4+2
y10-H3PO4
y1
y2
y9-H3PO4+2
y4-H3PO4
y6-H3PO4
y8-H3PO4
y9-H3PO4
Mass (m/z)
C
DHFAT(Phospho)IR
Tao2
Precursor mass: 470.2077 (-0.30 ppm)
b2
y5-H3PO4
a2
y1
b2-H2O
y2
y3-H3PO4
b3
y4-H3PO4
Mass (m/z)

Figure S4

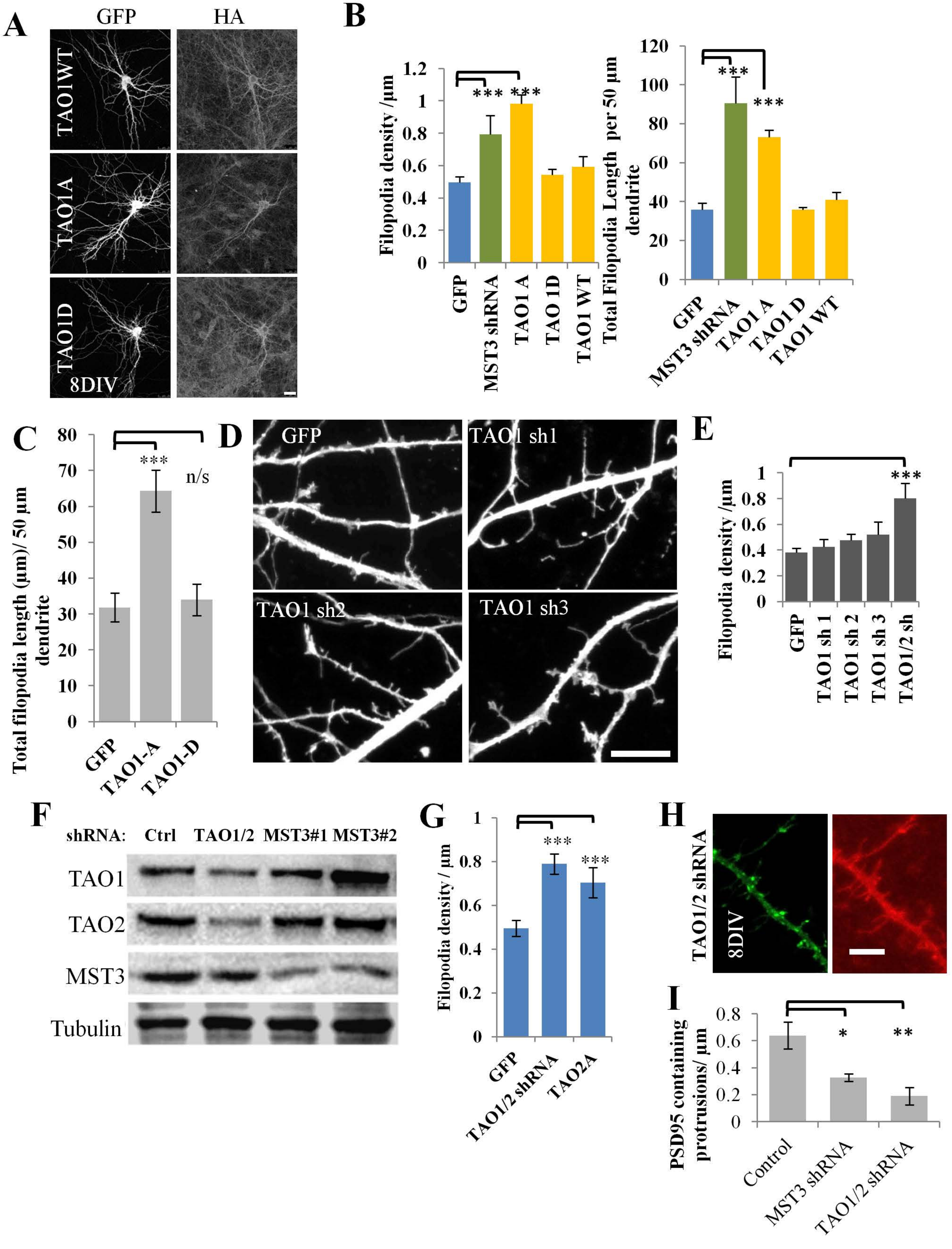
A
GFP
HA
TAO1WT
TAO1A
TAO1D
8DIV
B
Filopodia density /μm
***
GFP
MST3 shRNA
TAO1 A
TAO 1D
TAO1 WT
Total Filopodia Length per 50 μm dendrite
***
GFP
MST3 shRNA
TAO1 A
TAO1 D
TAO1 WT
C
Total filopodia length (μm)/ 50 μm dendrite
***
n/s
GFP
TAO1-A
TAO1-D
D
GFP
TAO1 sh1
TAO1 sh2
TAO1 sh3
E
Filopodia density /μm
***
GFP
TAO1 sh 1
TAO1 sh 2
TAO1 sh 3
TAO1/2 sh
F
shRNA: Ctrl TAO1/2 MST3#1 MST3#2
TAO1
TAO2
MST3
Tubulin
G
Filopodia density / μm
***
GFP
TAO1/2 shRNA
TAO2A
H
TAO1/2 shRNA
8DIV
I
PSD95 containing protrusions/ μm
*
**
Control
MST3 shRNA
TAO1/2 shRNA

Figure S5

A

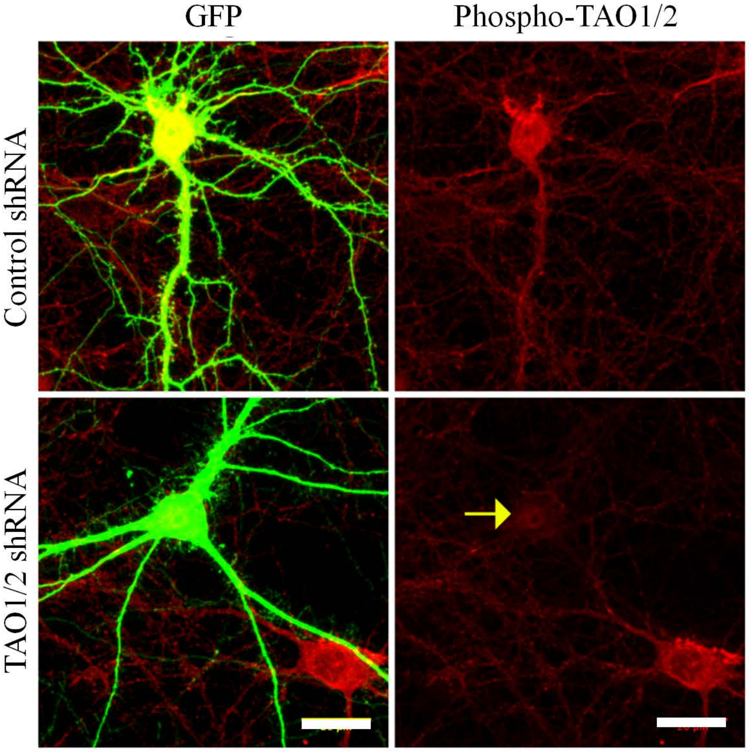

Figure S6

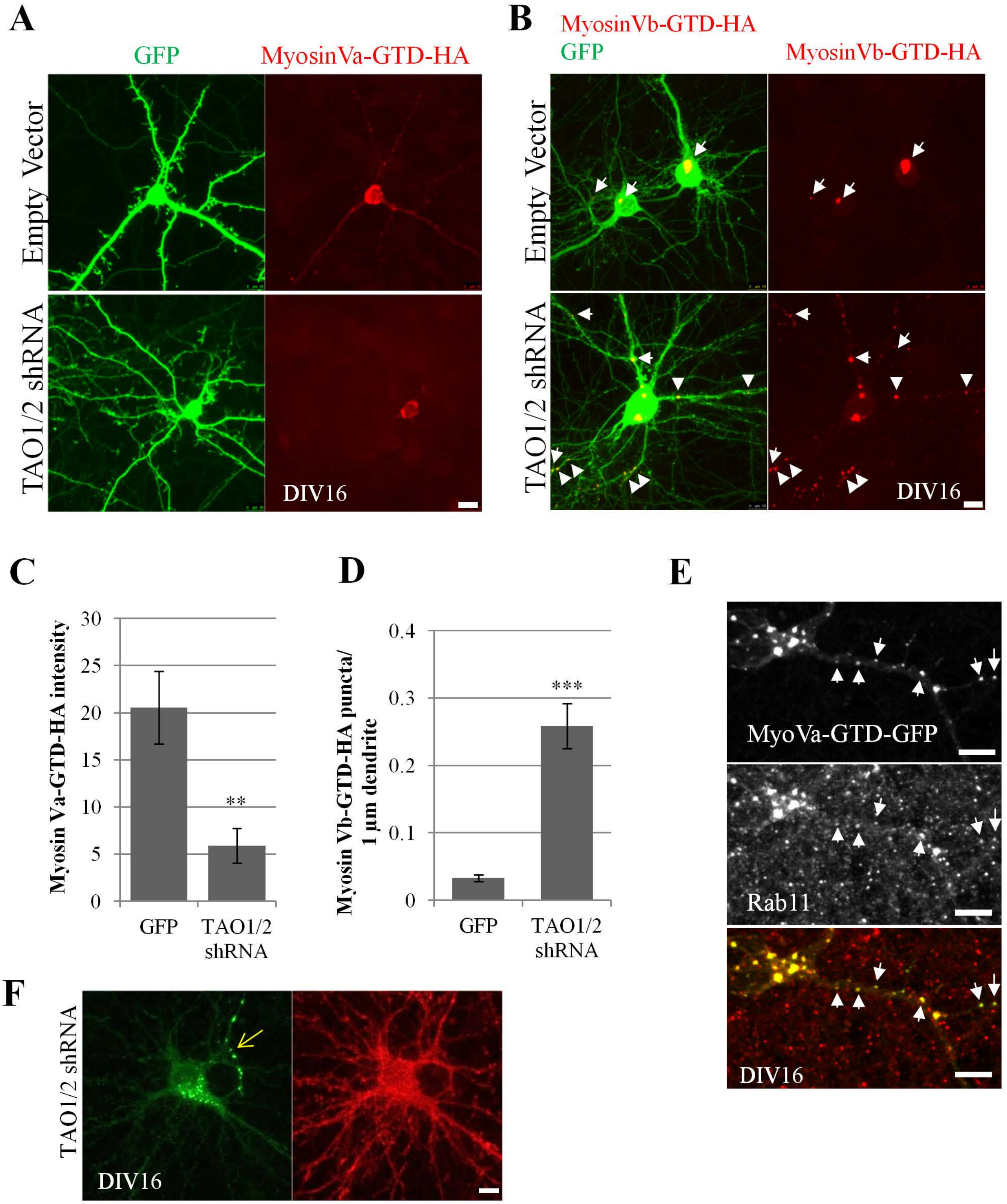
A
GFP
MyosinVa-GTD-HA
Empty Vector
TAO1/2 shRNA
DIV16
B
MyosinVb-GTD-HA
GFP
MyosinVb-GTD-HA
Empty Vector
TAO1/2 shRNA
DIV16
C
Myosin Va-GTD-HA intensity
30
25
20
15
10
5
0
**
GFP
TAO1/2 shRNA
D
Myosin Vb-GTD-HA puncta/ 1 µm dendrite
0.4
0.3
0.2
0.1
0
***
GFP
TAO1/2 shRNA
E
MyoVa-GTD-GFP
Rab11
DIV16
F
TAO1/2 shRNA
DIV16

SUPPLEMENTAL FIGURE LEGENDS

Figure S1: A& B. MST3 staining in cell bodies of neurons in hippocampal cultures are reduced. Empty vector or MST3 shRNA expressing vector transfected neurons are stained with a mouse monoclonal MST3 antibody. The staining in the cell body is significantly reduced (arrows and dotted line around the soma) ($p<0.001$) (n = 5 neurons each). Scale bar = 25 µm. C. Average filopodium length is also increased upon loss of function of MST3. D. Total filopodia length per 50 µm is not altered in MST3* (wild type MST3 that is shRNA resistant) expressing neurons (n = 6 for GFP and 5 for MST3*). E. Control shRNA does not alter length filopodia (n = 6 for GFP and 7 for control shRNA). F. Lentivirus was generated for pLentiLox 3.7 vector alone or vector expressing control shRNA (Ctrl sh), MST3 sh1 and MST3 sh2. Neurons were infected at DIV6 and protein lysates were obtained at 16 DIV. Endogeneous MST3 expression levels are reduced confirming knock-down (using rabbit polyclonal MST3 antibody). Tubulin antibody is used as a control for total protein levels. MST3 migrates at 60 kDa. Also see Figure S4F. G. Expression of HA tagged MST3-KD and HA-MST3* is detected by anti-HA antibodies for all data acquisition. Scale bar = 25 µm. H. MST3 shRNA #2 significantly increases dendrite total filopodia length per dendrite length, comparable to the first MST3 shRNA used (n = 7 neurons each). All error bars reflect standard errors.

Figure S2: A. GFP and MST3 shRNA expressing hippocampal neurons (magenta) immunostained with PSD-95 and Synapsin antibodies (teal) are shown. Colocalizations are shown in white. B. In MST3 shRNA expressing neurons protrusions co-localizing with synapsin is reduced $p < 0.05$. Similarly, in MST3 shRNA expressing neurons protrusions co-localizing with PSD-95 is reduced $p < 0.001$ (n = 5 cells each). All error bars reflect standard errors.

Figure S3: A. MST3-MG and MST3-KD expression and purification in HEK293 cells shown in coommassie staining. Half of the protein purified from a single 10 cm dish is loaded on the gel. About 8 µg of MST3-KD or MST3-MG is purified from one 10 cm plate. We used 3 or 4 plates for each construct in each experiment, therefore 25 – 30 µg of kinase is used for substrate labelling. B. Mass spectra of EPS8 and C. TAO2 phosphorylation site containing peptides are shown.

Figure S4: A. Hippocampal neurons transfected at DIV4 and imaged at DIV8 after GFP and HA immunostaining. TAO1wt, TAO1A and TAO1D are expressed in cytoplasm in cell body and dendrites. Scale bar = 25 µm. B. Filopodia density and total filopodia length per 50 µm of dendrite are shown for an experiment where neurons were transfected at DIV3 and imaged at DIV7. MST3 shRNA and TAO1A increases these measurements significantly (n = 21, 9, 14, 12 and 12 for GFP, MST3 shRNA, TAO1A, TAO1D and TAO1WT, respectively). C. TAO1A expression increases dendritic filopodia length when compared to GFP alone and TAO1D in hippocampal neurons transfected at DIV4 and imaged at DIV8 (n = 7, 7 and 10 for GFP, TAO1A, TAO1D, respectively). D. Representative images of dendrites expressing three separate TAO1 shRNAs (which do not target

TAO2) are shown. Scale bar is 7.5 µm. E. None of the three TAO1 shRNAs has any significant effect on filopodia density when expressed from 3DIV to 7DIV (n = 5, 6, 6, 5 and 6 neurons for GFP, TAO1sh1, TAO1sh2, TAO1sh3 and TAO1/2shRNA, respectively). TAO1/2 significantly increases filopodia density as shown earlier. F. Lentivirus expressing shRNA as labelled are used to infect cultured neurons at DIV6 and neuronal lysates were collected at DIV11. MST3 shRNAs are highly effective as shown earlier, TAO1/2 shRNA can effectively knock down TAO2 but also reduces TAO1 levels. Proteins migrate at, TAO1 120-130 kDa, TAO2 140-150 kDa, MST3 60 kDa. G. Filopodia density is also increased in TAO1/2 shRNA and TAO2A expressing neurons (n = 14, 14 and 6 neurons for GFP, TAO1/2 shRNA and TAO2A). H. PSD95-GFP and Td-tomato was expressed with TAO1/2 shRNA in hippocampal neurons. Scale bar = 5 µm. I. PSD95-GFP containing dendritic protrusion density is reduced in TAO1/2 shRNA expressing neurons (analysis also shown in Figure 2C & D). All error bars reflect standard errors.

Figure S5: PhosphoTAO1/2 immunostaining of hippocampal neurons transfected with GFP and control shRNA or GFP and TAO1/2 shRNA expressing plasmids is shown. Neurons were transfected at DIV11 and imaged at DIV16. TAO1/2 shRNA expression reduces phosphoTAO1/2 staining (arrow).

Figure S6: A. MyosinVa-GTD tagged with HA is co-expressed with a vector expressing GFP alone or GFP with TAO1/2 shRNA in dissociated cortical neurons. GFP is shown in green, HA antibody staining is shown in red. Images are maximum z projections of image stacks obtained by confocal imaging. Myosin Va-GTD-HA is localized to dendrites in control neurons but is restricted to the cell body in TAO1/2 shRNA expressing neurons. Cortical neurons were transfected at DIV 13-14 and fixed in 3 to 4 days. B. Myosin Vb- GTD-HA (red) is expressed in neurons with GFP or GFP together with TAO1/2 shRNA. The Myosin Vb- GTD puncta shown in red (right) is seen as yellow due to colocalization with GFP inside the neurons (left). Scale bars are 10 µm. C. Quantification of Myosin Va-HA intensity per pixel along one primary dendrite in neurons expressing GFP alone or TAO1/2 shRNA and GFP. In TAO1/2 shRNA expressing neurons Myosin Va-GTD-HA is reduced ($p < 0.01$). D. TAO1/2 shRNA causes increase in Myosin Vb-GTD-HA puncta in dendrites. Number of Myosin Vb-GTD-HA puncta is increased in TAO1/2 shRNA expressing neurons ($p < 0.001$). E. MyosinVa-GTD-GFP expressed in dissociated cortical neurons and stained by anti-Rab11. MyosinVa-GTD-GFP (green) is colocalized with Rab11 (red). Scale bar = 10 µm. F. In a subset of TAO1/2 shRNA expressing neurons, it can be clearly observed that axon is the only process that contains Myosin Va-GTD-GFP (yellow arrow). Scale bar = 10 µm. All error bars reflect standard errors.

Table S1:

The enrichment of proteins in Experiment 1 is shown as – values and in Experiment 2 as positive values. The columns are ordered with ascending median log2 value for Experiment 2. The number of unique peptides identified from each protein is shown in #peptides columns. Proteins that are enriched in phosphopeptide pulldown are shown in bold at the bottom of the list.

TABLE S1

Gene name	Uniprot Accession #	Protein Name	Experiment 1 median log2 (H/L)	# peptides	Experiment 2 median log2 (H/L)	# peptides
Hspa8	P63018	Heat shock cognate 71 kDa protein	-0.399124982	2	-1.365110729	2
Rps20	P60868	40S ribosomal protein S20	-0.147020233	2	-0.937942616	1
Rpl12	P23358	60S ribosomal protein L12	-0.261511777	7	-0.864809085	4
Srp9	D4A511	Signal recognition particle 9 kDa protein	-0.443591724	2	-0.733479106	1
Hist1h1e	P15865	Histone H1.4	-0.06698165	3	-0.731815826	2
Hspa5	P06761	78 kDa glucose-regulated protein	-0.443625045	24	-0.69294758	28
Hspa9	F1M953	Stress-70 protein, mitochondrial	-0.782047029	2	-0.688937034	29
H2afj	A9UMV8	Histone H2A.J	-0.953828454	1	-0.657363542	2
Grn	G3V8V1	Granulin, isoform CRA_c	0.137863359	6	-0.560106019	6
Spna2	C9EH87	Alpha II spectrin	-1.504200858	3	-0.556748581	1
Hspa8	P63018	Heat shock cognate 71 kDa protein	-0.499018389	34	-0.515123643	8
Rps13	P62278	40S ribosomal protein S13	-0.240927449	6	-0.50418133	2
Rps18	P62271	40S ribosomal protein S18	-0.272004482	8	-0.482541652	2
Rpl21	Q6PDW2	Protein LOC100361103	-0.098665616	4	-0.479671233	4
Rps18	P62271	40S ribosomal protein S18	-0.115569915	18	-0.417530945	10
Srp14	B2RYW7	Protein Srp14	0.651605447	2	-0.395293714	1
Rpl19	P84100	60S ribosomal protein L19	-0.094733424	3	-0.380379305	1
Rpl13a	P35427	60S ribosomal protein L13a	-0.055335579	9	-0.344522964	5
Hsp90ab1	P34058	Heat shock protein HSP 90-beta	-1.048915281	2	-0.342736594	4
Cltc	P11442	Clathrin heavy chain 1	-1.418683334	28	-0.32279553	3
Rpl18	P12001	60S ribosomal protein L18	-0.04171673	8	-0.313292994	3
Rpl7a	P62425	60S ribosomal protein L7a	-0.152221951	8	-0.295471287	7
Rpl24	P83732	60S ribosomal protein L24	-0.288948651	5	-0.282371389	4
Rpl8	P62919	60S ribosomal protein L8	-0.104484463	8	-0.28186538	4
Rpl23a	P62752	60S ribosomal protein L23a	0.088265913	5	-0.278614251	1
Rps19	P17074	40S ribosomal protein S19	-0.298973579	7	-0.266913982	4
Rpl6	F1LQS3	60S ribosomal protein L6	-0.097194996	12	-0.257134943	4
LOC100363800	Q6PDV8	RCG31311	-0.032982984	3	-0.241216334	2
Rpl10	Q6PDV7	60S ribosomal protein L10	-0.145797284	6	-0.23514841	3
Rps6	P62755	40S ribosomal protein S6	-0.262738516	5	-0.233490003	3
Rps8	B2RYR8	40S ribosomal protein S8	-0.274414909	8	-0.224696283	4
Rpl3	P21531	60S ribosomal protein L3	-0.076637333	15	-0.218307598	2
Rps14	P13471	40S ribosomal protein S14	-0.210862718	4	-0.21216553	1
Rpl30	P62890	60S ribosomal protein L30	-0.1714008	7	-0.20820505	3
Rps16	B0K038	Rps16 protein (Fragment)	-0.307722791	7	-0.174471461	3
Rplp2	P02401	60S acidic ribosomal protein P2	-0.274890907	7	-0.156321318	4
Rpl17	P24049	60S ribosomal protein L17	-0.07974843	8	-0.135015907	5
Thoc4	D3ZXH7	Protein Thoc4	-0.07806751	3	-0.13136143	1
Tpi1	P48500	Triosephosphate isomerase	-0.87872201	3	-0.131101117	1
Rps27l	P24051	40S ribosomal protein S27-like	-0.574733351	2	-0.120547393	1
Rpl15	P61314	60S ribosomal protein L15	-0.031099906	9	-0.119268311	5
Rps24	P62850	40S ribosomal protein S24	-0.040546735	5	-0.11919208	2
Rpl14	Q63507	60S ribosomal protein L14	0.234782096	4	-0.118774354	3
Map1b	F1LRL9	Microtubule-associated protein 1B	-0.468395268	60	-0.114418933	30
Ywhag	P61983	14-3-3 protein gamma	-2.09769028	4	-0.113774966	4
Rps3	P62909	40S ribosomal protein S3	-0.273943724	13	-0.113394676	6
Rps25	P62853	40S ribosomal protein S25	-0.192274417	4	-0.095271837	2
Rpl10a	P62907	60S ribosomal protein L10a	-0.087682664	8	-0.083715703	6
Tubal3	F1LUM5	Protein Tubal3	-1.809313376	1	-0.0833177	1
Rpl18a	P62718	60S ribosomal protein L18a	-0.053583056	6	-0.07772285	4
Rpl9	P17077	60S ribosomal protein L9	-0.065834006	10	-0.07319407	3
Hspa4	F1LRV4	Heat shock 70 kDa protein 4	-0.612575296	13	-0.073036819	7
Rpl23	P62832	60S ribosomal protein L23	-0.134731598	4	-0.064737894	3
Eif2s2	Q6P685	Eukaryotic translation initiation factor 2, subunit 2	-0.503936359	1	-0.059827369	1
Dpysl2	P47942	Dihydropyrimidinase-related protein 2	-1.253256947	10	-0.05144462	1
Fau	P62864	40S ribosomal protein S30	-0.06504867	1	-0.042689416	1
Srp19	B2RZ66	Protein Srp19	-0.17917254	3	-0.04114428	2
Rps7	P62083	40S ribosomal protein S7	-0.143824671	5	-0.032098054	4
Rpl27a	P18445	60S ribosomal protein L27a	-0.150563345	5	-0.012631752	2
Rps11	P62282	40S ribosomal protein S11	-0.119886604	7	-0.007417429	1
Rplp0	P19945	60S acidic ribosomal protein P0	-0.017583379	9	0.003599478	2
Spock1	F1M6V6	Protein Spock1 (Fragment)	-0.711211518	3	0.006528259	3
Rpl36al	B2RYQ8	Large subunit ribosomal protein L36a, isoform CR	-0.185730203	1	0.015658366	1
Gpc2	P51653	Glypican-2	-0.037629539	15	0.031070441	7
Rps15	P62845	40S ribosomal protein S15	-0.033111539	4	0.046244442	2
Rps3a	P49242	40S ribosomal protein S3a	-0.318526482	12	0.052180488	1
LOC100361060	D3ZZ95	60S ribosomal protein L36	-0.176532923	5	0.060384571	3
Atp5o	Q06647	ATP synthase subunit O, mitochondrial	-1.500896589	1	0.073727114	1
Ncl	P13383	Nucleolin	-0.272889451	16	0.075806424	8
Rpl32	P62912	60S ribosomal protein L32	-0.054293606	6	0.090573252	2
Rplp1	P19944	60S acidic ribosomal protein P1	-0.198654787	5	0.095049451	3
Cfl1	P45592	Cofilin-1	-1.572351763	2	0.102591096	4
Ncam1	F1LNY3	Neural cell adhesion molecule 1 (Fragment)	-1.800193522	6	0.11494048	1
Mapt	A0JN25	Microtubule-associated protein	-1.184820572	4	0.127122607	2
	Q00715	Histone H2B type 1	-0.41356892	4	0.132987233	2
Rps4x	P62703	40S ribosomal protein S4, X isoform	-0.119472201	15	0.14115858	4
Gapdh	P04797	Glyceraldehyde-3-phosphate dehydrogenase	-1.367866921	6	0.145520803	4
Calm1	P62161	Calmodulin	-0.718590137	5	0.196528507	5
Hnrnpu	Q63555	SP120	-0.435388651	7	0.196644436	3
Set	B0BMV1	Set protein	-0.645332691	4	0.212513847	3
Slc25a4	Q05962	ADP/ATP translocase 1	-1.117753287	8	0.232408924	6
Tuba1a	P68370	Tubulin alpha-1A chain	-1.078601992	1	0.256041071	1
Ywhae	P62260	14-3-3 protein epsilon	-2.559589067	3	0.286264436	4
Tubb6	Q4QQV0	Protein Tubb6	-2.091860172	2	0.294778133	1

Dcx	G3V997	Neuronal migration protein doublecortin	-1.101307055	6	0.296488546	4
Spock2	D3ZGJ7	Protein Spock2	-0.012452992	7	0.301046308	3
Pdia6	Q63081	Protein disulfide-isomerase A6	-0.665437468	2	0.33025525	3
Rps17	P04644	40S ribosomal protein S17	-0.130694285	5	0.334985206	2
Map1lc3a	Q6XVN8	Microtubule-associated proteins 1A/1B light chair	-1.265554368	1	0.338755877	1
Tubb2a	P85108	Tubulin beta-2A chain	-1.149510052	1	0.392077352	1
Tubb4a	B4F7C2	Protein Tubb4a	-1.091121976	4	0.45044024	3
Atp5b	G3V6D3	ATP synthase subunit beta	-1.524593296	14	0.460329855	3
Gpc1	P35053	Glypican-1	-0.457648402	2	0.514882773	1
Eef1a1	P62630	Elongation factor 1-alpha 1	-1.226260648	7	0.522591038	6
Rps5	B0BN81	Ribosomal protein S5, isoform CRA_b	-0.310358241	7	0.525872328	1
Tubb3	Q4QRB4	Tubulin beta-3 chain	-0.966886877	9	0.529305758	9
Tubb5	P69897	Tubulin beta-5 chain	-0.836486106	4	0.54397738	4
Txndc12	B0BN97	Txndc12 protein (Fragment)	-0.82770812	5	0.56331322	4
Map2	P15146	Microtubule-associated protein 2	-0.697944675	2	0.587798797	1
Ywhaz	P63102	14-3-3 protein zeta/delta	-2.515144732	5	0.628137753	5
Hist1h4b	P62804	Histone H4	-0.727142319	2	0.668213658	2
Atp5j2	D3ZAF6	ATP synthase subunit f, mitochondrial	-1.083544429	1	0.732193194	2
Ptges3	B2GV92	Ptges3 protein	-0.692101651	1	0.739069989	3
Ptma	P06302	Prothymosin alpha	-0.459784655	3	0.79872543	3
Naca	B2RYX0	Naca protein	2.965399988	1	0.823722255	1
Rpl39	P62893	60S ribosomal protein L39	-0.17300442	2	0.825780943	1
Ptms	B3DM95	Parathymosin	-0.214443584	2	0.943979344	1
Syt1	P21707	Synaptotagmin-1	-0.474652716	5	0.982138522	2
Npm1	P13084	Nucleophosmin	-0.405452998	1	1.115839424	2
Nap1l4	Q5U2Z3	Nucleosome assembly protein 1-like 4	-0.560560592	1	1.45371248	1
Tubb4b	Q6P9T8	Tubulin beta-4B chain	-0.686514448	1	1.458275542	1
Gpc4	Q642B0	Glypican 4	0.184623513	10	2.039738658	2
Tubb2b	Q3KRE8	Tubulin beta-2B chain	-1.02436493	1	2.042634012	1
Ppm1g	Q8K3W9	Protein phosphatase 1G (Formerly 2C), magnesiur	-1.13921528	1	2.133729301	1
Actb	P60711	Actin, cytoplasmic 1	-1.145454531	1	2.281321553	1
**Myo5a**	**Q9QYF3**	**Unconventional myosin-Va**	**-2.069855733**	**2**	**2.328145267**	**50**
**Dbn1**	**C6L8E0**	**Drebrin E**	**-1.380815233**	**1**	**2.660874407**	**11**
**Myl6**	**B2GV99**	**Myl6 protein**	**-1.856566114**	**2**	**2.988525252**	**9**

SUPPLEMENTAL EXPERIMENTAL PROCEDURES

Neuronal cultures

Hippocampal neurons were cultured from E19 Long-Evans rats (Charles River Lab) and plated at a density of 150,000 neurons per 18 mm glass coverslips (Fisher) coated with 0.06 mg/ml poly-D-lysine (Sigma) and 0.0025 mg/ml laminin (Sigma). Neurons were plated with plating media containing 10% Fetal bovine serum (Hyclone), 0.45% dextrose, 0.11 mg/ml sodium pyruvate, 2 mM glutamine in Modified Eagle Medium. Cultures were transferred to maintenance media after 4 hours containing 1X B27 (Invitrogen), 100 units/ml penicillin and 100 mcg/ml streptomycin, 0.5 mM glutamine, 12.5 µM glutamate in Neurobasal Media (Invitrogen). Half of the media was replaced with fresh media every 4 days. Neurons were transfected with ~ 0.5 µg plasmid DNA using Lipofectamine-2000 (Invitrogen) following manufacturer's guidelines.

DNA constructs, shRNA and lentiviruses

Mouse MST3 (BC004650) cDNA purchased from ATCC was cloned in pRK5 mammalian expression vector with N-terminal HA tag. MST3 kinase dead K53R and MST3 analog sensitive mutations M99A and M99G were generated by site directed mutagenesis. A MST3 shRNA#1 resistant construct (MST3*) was created by introducing silent mutations changing 6 base pairs resulting in 5'GGGCCTAGACTATCTTCAC3' by site directed mutagenesis. TAO1 resistant to TAO1/2 shRNA was generated by site directed mutagenesis introducing silent mutations changing 6 base pairs resulting in 5'GGGCAGTACGACGGAAAG3'. An shRNA with no substantial match to mouse or rat mRNA sequences targeting 5'AGACCCAAGGATTAGAAGG 3' was used as a control (shCtrl). Myristoylation tagged membrane targeted Td-tomato was cloned in pcDNA3.0 expression vector. Human FMNL2 (BC167159), human ArhGAP18 (BC111940), mouse GIPC (BC003490), mouse EPS8 (BC016890), human TAO1 (shorter isoform 853 amino acids, BC144067), human PAK6 (BC035596) were purchased from Open Biosystems/ Thermo Scientific and cloned into Prk5-HA vector via PCR cloning with NotI and SalI sites in frame with N-terminal HA tag. Human full length TAO2 (1235 amino acids) in pCMV Sport 6.0 expression vector was a gift from Dr. Froylan Calderon De Anda and Dr. Li-Huei Tsai from MIT (de Anda et al., 2012). Phosphorylation site mutations to Alanine or Aspartate were generated with site-directed mutagenesis. Myosin Va globular tail domain-GFP, Myosin Va- GTD-HA and Myosin Vb -GTD-HA vectors were generous gifts from Dr. Don Arnold from University of South California (Lewis et al., 2009). Drebrin pEGFP-C1 was a gift from Dr Wiebke Ludwig-Peitsch. PSD-95-GFP construct was a gift from Roger Nicoll's lab at UCSF.

All shRNA sequences were 19 base pairs long and were selected via http://katahdin.cshl.org/html/scripts/main.pl. shRNA target sequences on MST3 were sh #1 5’AGGACTTGATTATCTACAC3’ and sh #2 5’ GAAAGGACTTGATTATCTA3’. shRNA targeting sequences for rat TAO1 are: 5’CTAAGAGTTTGAAGTCTAA3’, 5’CTTAGAACATGCAATGTTA 3’and 5’ TGGAGAAACTTATTAAGAA 3’; rat EPS8 are 5’CCAGAGTGTTCAGTCAAA3’, 5’TACTTGATGCCAAGGGTAA 3’and 5’CCTGGCTCTCAAGTCAACC 3’ and for human MST3 used in HEK293T cells are 5’CAGTCCATATGTAACCAAA 3’and 5’ CAGTGTTTATCTACAATTA 3’. Rat TAO2 and to a lesser extend TAO1 was knocked down by TAO1/2 shRNA with sequence: 5’ GGGACAATATGATGGCAAA 3’, other two rat TAO2 shRNAs are 5’ GAGAGGACTTGAATAAGAA3 and 5’CACCCACAGTCATCATGGA 3’. Hairpins targeting these sequences were cloned in pLentiLox 3.7 which expresses EGFP via a separate promoter in addition to expressing shRNA via a U6 promoter. Empty pLentiLox 3.7 vector was used as a cell fill to visualize neurons and referred to as GFP. Lentivirus expressing empty pLentiLox 3.7 and expressing control shRNA, MST3 sh#1, MST3 sh#2, TAO1/2 shRNA #1 were generated at UCSF core facilities. All clones were verified by sequencing.

In Utero Electroporation

Timed pregnant C57Bl6 mice were used for *in utero* electroporation for the first set of experiments with MST3 shRNA. Surgery was performed at E14.5-15.5, when mice were anesthetized with sodium pentobarbital at 50 mg/kg body weight. A midline incision was made and the uterus was exposed. DNA solution including the plasmid and 0.04% trypan blue was injected into the lateral ventricle with a glass micropipette. After injection, embryos were subjected to electrical pulses (32Vfor E14.5, 36V for E15.5) with 50 ms duration that were delivered five times at 950 ms interval using a square-pulse electroporator BTX830. Embryos were injected with either 1 μg/μl pCAG-GFP (control) or 0.4 μg/μl pCAG-GFP + 1 μg/μl MST3 shRNA #1 in pLenti-Lox 3.7. For a second set of experiments timed pregnant Parkes mice were used. In these experiments animals were anaesthetized with isofluorane during the surgery. Embryos were injected with either 1 μg/μl pCAG-GFP (control) or 0.4 μg/μl pCAG-GFP + 1 μg/μl MST3 shRNA #1 or 0.4 μg/μl pCAG-GFP + 1 μg/μl TAO1/2 shRNA in pLenti-Lox 3.7. After the procedure the abdominal wall and skin were sutured to allow the embryos to develop full term. Litters were inspected for GFP expression in the cortex by fluorescent goggles in first week (Nightsea). Mice were perfused at P18-P20 using 4% paraformaldehyde and 4% sucrose. Brains were post-fixed in the same fixative solution at 4°C. 100 μm thick coronal sections were obtained using cryostat sectioning. Sections were dried on glass slides, blocked by 10% Normal Donkey Serum and 0.5% Triton-X and immunostained by mouse GFP antibody (1:1000, Roche) in blocking buffer, followed by goat anti-mouse conjugated with Alexa-488 (1:500, Invitrogen).

Immunocytochemisty

Following antibodies were used for immunostainings in cultured neurons: chicken anti-GFP (1:2000) Aves Labs, mouse anti-MST3 (1:1000) BD Transduction Labs, rabbit anti-MAP2 (1:1000) Chemicon, rat anti-HA 1:500 (Roche), mouse anti-PSD95 (1:200) Thermo-Fisher (6G6-1C9), mouse anti-Rab11 (1:100) BD Transduction Labs, rabbit anti-Synapsin I (1:1000) Millipore, rabbit anti-phospho TAO1/2 1:500. Rabbit polyclonal phospho TAO1/2 antibody was raised against peptide CNRDHFAT*IRTASL of moue TAO2 with phospho T 475 by Yenzym (San Francisco, CA).

Western Blots

We have used rat anti-HA 1:2000 (Roche), mouse anti-MST3 (1:1000) BD Transduction Labs and rabbit anti-MST3 (1:1000) Epitomics, mouse anti-TAO1 (1:1000) BD Biosciences, mouse anti-beta tubulin (1:1000) Covance, goat-anti TAO2 (1:1000) Santa Cruz and rabbit anti-thiophosphate ester (1:5000- 10,000) Epitomics. HRP conjugated secondary antibodies (Jackson Immunoresearch) were used.

Confocal Microscopy

Dendrites were imaged using an inverted Leica SP5 confocal microscope using a 63X (NA 1.4) objective at 6X zoom. Z sections were obtained across the dendrite depth at 0.5 μm z- intervals. Spine density and categorization was done manually using Leica image analysis. Spine head diameter measurements were done using a custom plugin in ImageJ (Ultanir et al., 2007).

Quantification of PhosphoTAO1/2 Staining in Cultures

Cultured rat hippocampal neurons were transfected with either control scrambled shRNA or shRNA against MST3 at DIV13 using Lipofectamine2000, and then fixed using 4%PFA+4% sucrose at DIV16 for 10 min at room temperature. Neurons were then incubated with blocking solution (10% normal donkey serum+ 0.2M glycine+0.2% triton x-100 in PBS) for an hour followed by incubation with primary antibody against p-TAO1/2 (rabbit polyclonal) and GFP (monoclonal from Roche) at 4 degrees overnight. After 3 washes with PBS, neurons were incubated for 2 hours at room temperature with Alexa 488 and 568 secondary antibodies. Coverslips were mounted using fluoromont after six washes with PBS. Imaging was performed on a scanning disk confocal SP5 Leica system using 40X, 1.25N objective at 1.5 times zoom. Image stacks were acquired at z spacing of 0.4 microns. Quantification of pTAO staining was performed using ImageJ Measure function. Average projection of Image stacks were acquired using ZProject. The fluorescence in the neuronal soma which was chosen as ROI was measured using Measure function in ImageJ. The fluorescence intensity of untransfected neighboring neuronal soma which was absent in GFP staining was used as internal control in each image. The normalized fluorescence intensity of neuronal soma from ten neurons per

experiment was calculated and the average of three different experiments was then plotted. Two tailed-unequal variance Student t-test was used to calculate p-values.

MST3 Kinase assays

HA-tagged MST3 was expressed in COS-7 or HEK293T cells for 48 hours which are maintained in a medium containing 10% FBS, 1 X penicillin/ streptomycin in Dulbecco's Modified Eagle Medium. MST3 kinase was purified via HA epitope tag using Anti-HA Affinity matrix (Roche, clone 3F10). Prior to lysis cells expressing MST3 (except kinase dead MST3) were incubated with 0.5 µM O.A. for 1 hour. Lysis buffer contained 1% Nonidet P-40, 10% glycerol, 1 mM Na3VO4, 20 mM β-glycerol phosphate, 50 mM NaF, 1 X complete protease inhibitor cocktail (Roche), 1 X phosphatase inhibitor cocktail I or III (Sigma) in 20 mM Tris-HCL pH 8.0 and 150 mM NaCl. Lysis was achieved by incubation on ice for 30 min. The cells were pipetted up and down several times before centrifugation at 20,000 g for 15 min at 4° C. Supernatant containing the solubilized proteins was precleared using IgG- Sepharose (GE Healthcare) for 30 min at 4° C. HA-MST3 was purified with HA-affinity matrix for 2 hours at 4° C. Beads were washed with lysis buffer twice, incubated with 1 M NaCl for 10 min followed by a 10 min wash with lysis buffer at 4° C. Beads were washed twice with a kinase reaction buffer not including ATP. Kinase assay was conducted in 20 mM Tris-HCl pH 7.5, 10 mM MgCl2, 1 mM dithiothreitol (DTT), 100 µM ATP, 1x Phosphatase inhibitor cocktail and 0.5 mM of one of the following four ATP analogs: ATP-γ-S, 6-Bn-ATP-γ-S (Bn), 6-PhEt-ATP-γ-S (Phe) or 6-Furfuryl-ATP-γ-S (Ff) (BioLog Life Science Institute) for 30 min at 30 ° C. Reaction was followed by alkylation for 1 hour at room temperature by addition of 2 µl 100 mM p-nitro mesylate (PNBM) per 30 µl of kinase reaction. Proteins were solubilized by sample buffer and ran at 4-12 % bis –tris gels (Invitrogen). MST3 autophosphorylation was detected by anti-thiophosphate ester antibody (1:10,000 – 1:5,000, Epitomics) followed by HRP conjugated secondary antibody.

MST3 Substrate Labelling

Covalent capture method for kinase substrate identification was done as previously described (Ultanir et al., 2012). Briefly, MST3 kinase was purified from COS-7 or HEK293T cells on HA-beads as described above. Cells expressing MST3-MG were treated with 0.5 µM okadaic acid for 1 hour. Untransfected cells and cells expressing MST3-KD were used as negative controls. Cells were lysed with 20 mM Tris HCL, 150 mM NaCl, Protease inhibitor cocktail, phosphatase inhibitor I (Sigma), 10% glycerol and 1% NP40. Lysate was incubated on ice for 30 min, followed by centrifugation at 20,000g for 15 min. Supernatant was pre-cleared with IgG sepharose beads. 50 µL 1:1 slurry anti-HA resin was incubated with protein lysate for 1,5 – 3 hours. P3 and P13 mouse brain lysates were obtained in 0.25% NP-40, 10 mM MgCl2, 100 Mm NaCl, 20 mM Tris pH=7.5, 0.5 mM

DTT and 1X protease inhibitor cocktail. 100 µl brain lysate of 20 µg/µl protein concentration was used to be labeled by MST3-MG or MST3-KD containing beads, each. A lysate only sample was used in which no HA beads were added as a second negative control for each experiment. Total 4 experiments were conducted twice with P13 and twice with P3 brain lysates. Labelling reaction contained 1 µM cyclic AMP- dependent protein kinase inhibitor, 10 µM protein kinase C inhibitor (Bisindolylmaleimide I – Calbiochem), 3 mM GTP, 100 µM ATP, 1 µM O.A and 0.5 mM Benzyl-ATP-γ-S.

Multiplex SILAC labelling of neuronal cultures

Cortical neuronal cultures were labelled using Multiplex SILAC as described (Zhang et al., 2011). Briefly, 5- 8 million cortical neurons from E18.5 rat embryos were plated per 10 cm culture dishes coated as described above. Same culture media was used as the hippocampal neurons with the exception of Neurobasal media free of L-Arginine and L-Lysine (Invitrogen) was used. Media was supplied with either Lysine 8 (U-13C6; U-15N2) and Arginine 10 (U-13C6; U-15N4) or Lysine 4 (4, 4, 5, 5-D4) and Arginine 6 (U-13C6). All isotopes were obtained from Cambridge Isotope Laboratories, Inc. 200 mg/L L-Proline was also included in the culture medium. Half of the culture media was replaced with new media every 3 -4 days. 1 µM Ara-C was included in the medium when the medium was replaced in to inhibit glial growth. Protein was extracted from three 10 cm dishes at DIV9 in 20 mM Tris pH = 8.0, 150 mM NaCl, 1% NP-40, 1 mM Okadaic acid and 1X protease inhibitor. Total 2.4 mg protein was obtained in a volume of 300 µl for Lys4Arg6 and Lys8Arg10 each.

Phospho-Peptide pull-down

Peptide pull-down using SILAC labeled lysates was described before (Stephanowitz et al., 2012). TAO2 T475 peptide (C RNRDHFAT*IRTASLVSR) was synthesized with or without a phosphorylation at the indicated Threonine (Elim Biopharmaceuticals). Peptide was conjugated to iodoacetyl agarose beads (Sulfolink Resin, Thermo Scientific) as described in manufacturer's protocol. 30 µl resin conjugated to 22 nmol of peptide was used to for pull-down in 1.6 mg SILAC labeled protein lysate. Beads were incubated with lysate for 1.5 hours at room temperature. Beads were washed four times with lysis buffer and two additional times with lysis buffer without NP40 or protease inhibitors. Precipitated proteins were subjected to in-bead typsin digest.

Reversed-phase liquid chromatography-electrospray tandem mass spectrometry (LC-MS/MS) analysis

In-beads digestion. After incubation with the SILAC cell extracts, phospho and non phospho peptide conjugated beads were washed with the conjugation buffer twice, and then mixed. After a short spin

the supernatant was removed, and beads (approximately 60 µl) were resuspended in 100 µl 8M urea in the presence of 80 mM ammonium bicarbonate. Samples were reduced by incubating for 15 min at 60ºC in the presence of 2.5 mM DTT. After this, samples were alkylated by incubation with 3 mM iodoacetamide for 1h in the dark at room temperature. Remaining iodoacetamide was quenched by adding DTT to a final concentration of 3 mM, and incubating at 37 C for 15 min. Samples were then diluted to a final concentration of 2 M urea, and digested overnight at 37ºC using 4 µg sequencing grade modified trypsin (Promega, Madison, WI). Concentration of ammonium bicarbonate during digestion was 20 mM, and pH was confirmed to be around 8. After digestion, samples were added formic acid to a final concentration of 4%, and peptides were extracted using C18 ZipTips (Waters) according to the manufacturer's protocol. Eluates of the ZipTips were vacuum-evaporated, and peptides resuspended in 7 µl 0.1% formic acid in water.

Reverse-phase LC-MS/MS Analysis. The digests were separated by nano-flow liquid chromatography using a 75-µm x 150-mm reverse phase 1.7 µm BEH 130 C18 column (Waters) at a flow rate of 600 nL/min in a NanoAcquityTM Ultra performance UPLC system (Waters). Mobile phase A was 0.1% formic acid in water and mobile phase B was 0.1% formic acid in acetonitrile. Following equilibration of the column in 2% solvent B, an aliquot of each digest (5 µl) was injected, then the organic content of the mobile phase was increased linearly to 40% over 90 min, and then to 50% in 1 min. The liquid chromatography elute was coupled to a hybrid linear ion trap-Orbitrap mass spectrometer (LTQ-Orbitrap Velos, Thermo Scientific, San Jose, CA) equipped with a nanoelectrospray ion source. Spraying was from an uncoated 15-µm-inner diameter spraying needle (New Objective, Woburn, MA). Peptides were analysed in positive ion mode and in information-dependent acquisition mode to automatically switch between MS and MS/MS acquisition. MS spectra were acquired in profile mode using the Orbitrap analyzer in the m/z range between 350 and 1500. For each MS spectrum, the 6 most intense multiple charged ions over a threshold of 2000 counts were selected to perform HCD experiments. Product ions were analysed on the Orbitrap in centroid mode. A dynamic exclusion window of 0.5 Da was applied that prevented the same m/z from being selected for 45 s after its acquisition.

Peaklists were generated using PAVA in-house software (Guan et al., 2011), based on the RawExtract script from Xcalibur v2.4 (Thermo Fisher Scientific, San Jose, CA). The peak lists were searched in three separate searches against the rat subset of the UniProt database as of March 21, 2012, using in-house ProteinProspector version 5.10.10 (a public version is available on line). A randomized version of all entries was concatenated to the database for estimation of false discovery rates in the searches. Peptide tolerance in searches was 20 ppm for precursor and 20 ppm for product ions, respectively. Peptides containing two miscleavages were allowed. Carbamidomethylation of cysteine was allowed as constant modification; acetylation of the N terminus of the protein, pyroglutamate formation from

N terminal glutamine and oxidation of methionine, were allowed as variable modifications in initial searches. A second search was performed allowing also for 2H(4) labelling in lysine and 13C(6) labelling in arginine as constant modifications; and a third search allowing for 15N(2) 13C(6) labelling in lysine and 15N(4) 13C(6) labelling in arginine as constant modifications. In all cases, the number of modification was limited to two per peptide. The 3 searches merged into a single result file. A minimal ProteinProspector protein score of 20, a peptide score of 15, a maximum expectation value of 0.05 and a minimal discriminant score threshold of 0.0 were used for initial identification criteria. Protein hits were considered significant when at least two peptide sequences matched a protein entry and the Prospector score was above the significance level. For identifications based on one single peptide sequence with high scores, the MS/MS spectrum was reinterpreted manually by matching all the observed fragment ions to a theoretical fragmentation obtained using MS Product (Protein Prospector) (Clauser et al., 1999).

Quantification—SILAC quantification measurements were extracted from the raw data by Search Compare in Protein Prospector (http://prospector.ucsf.edu). Search Compare averaged together MS scans from -10 s to +30 s from the time at which the MS/MS spectrum was acquired in order to produce measurements averaged over the elution of the peptide. SILAC ratios (medium heavy/heavy) were calculated, and base 2 logarithms of these values were used. If quantitative data were available from isotopic envelopes identified as different charge states of the same peptide, the median of the log2 of the calculated SILAC ratios was used for that peptide. For proteins, the median of all the log2 ratios for peptides unique to that protein was calculated. Protein log2 ratios were plotted in a histogram with bin size 0.2, and log2 ratios were then corrected for differences in protein level between the heavy and medium heavy sample using the deviation from 0 of the mode of this distribution.

Co-immunoprecipitation in HEK293T cells

HEK293T cells were transfected with Myosin Va GTD-GFP or control GFP construct, HA tagged TAO1 wild type and HA tagged TAO1 phosphomutant T440A separately. Cell lysates were made in lysis buffer (20mM Hepes pH 7.4, 150mM NaCl, 1% Triton-X100, 0.5% Deoxycholate, 0.1% SDS, 2mM EDTA, 2mM DTT with protease inhibitor cocktail from Roche). Lysate from Myosin Va-GFP or control GFP transfected cells was incubated with Protein G beads prebound with monoclonal GFP antibody, and then lysates from either HA-TAO1-wildtype or HA-TAO1-A phosphomutant transfected cells were added and incubated for 3 hours at 4 degrees with rotation. Beads were then washed thrice with lysis buffer, twice with lysis buffer without detergent and then sample buffer was added. Samples were run after boiling on a 4-12% gradient Bis-Tris gel, transeferred on PVDF membranes and then blotted with antibodies against HA to detect presence of TAO1 and GFP to detect equal load of GFP or Myosin Va-GFP.

Immunoprecipitation from neuronal lysates

Briefly, DIV6 or DIV9 rat embryonic cultured hippocampal neurons were lysed in HKT buffer (20mM Hepes pH7.4, 150mM KCl, 0.5% TritonX100, 1mM DTT, 1mM EDTA and protease inhibitor cocktail) and incubated with Protein A beads for 30 min at 4 degrees. Pre-cleared supernatant was collected and incubated with Myosin Va antibody bound Protein A beads (Sigma) overnight at 4 ºC. Beads were then washed thrice with HKT buffer followed by three times with buffer without detergent. Beads were collected and boiled after addition of sample buffer before running on a 4-12% Bis-Tris gel. After transfer on to PVDF membrane, blots were probed with TAO1 mouse antibody (BD biosciences), Myosin Va rabbit antibody (Sigma) and pTAO1/2 rabbit antibody.

Statistics

Multiple data sets were compared using ANOVA and pairwise comparisons of datasets were done using student's t test using Excel. On figures * = $p < 0.05$, ** = $p < 0.01$ and *** = $p < 0.001$, in Student's t-test. Comparisons of percentage data in Myosin Va localization was done using chi-square test. All error bars are standard errors of the mean.

Supplemental References

Clauser KR, Baker, PR, Burlingame, AL. (1999). Role of accurate mass measurement (+/- 10 ppm) in protein identification strategies employing MS or MS/MS and database searching. Anal. Chem. 71, 2871–2873.

Guan S, Price JC, Prusiner SB, Ghaemmaghami S, Burlingame AL. (2011). A data processing pipeline for mammalian proteome dynamics studies using stable isotope metabolic labeling. Mol Cell Proteomics. (12):M111.010728.
