# Supplementary material for: MST3 Kinase Phosphorylates TAO1/2 to Enable Myosin Va Function in Promoting Spine Synapse Development
Source: Neuron. 2014 Dec 3;84(5):968–82. doi: 10.1016/j.neuron.2014.10.025 (PMC4407996; doi:10.1016/j.neuron.2014.10.025)
Supplement: Document S2. Article plus Supplemental Information [file mmc2.pdf]

Cell Press

Neuron
Article

MST3 Kinase Phosphorylates TAO1/2 to Enable Myosin Va Function in Promoting Spine Synapse Development

Sila K. Ultanir,[1,5,6,*] Smita Yadav,[1,4,6] Nicholas T. Hertz,[2,4] Juan A. Oses-Prieto,[3] Suzanne Claxton,[5] Alma L. Burlingame,[3] Kevan M. Shokat,[2,4] Lily Y. Jan,[1,4] and Yuh-Nung Jan[1,4,*]
[1]Departments of Physiology, Biochemistry, and Biophysics, University of California, San Francisco, San Francisco, CA 94158, USA
[2]Department of Cellular and Molecular Pharmacology, University of California, San Francisco, San Francisco, CA 94158, USA
[3]Department of Pharmaceutical Chemistry, University of California, San Francisco, San Francisco, CA 94158, USA
[4]Howard Hughes Medical Institute, University of California, San Francisco, San Francisco, CA 94158, USA
[5]Medical Research Council, National Institute for Medical Research, The Ridgeway, Mill Hill, London NW7 1AA, UK
[6]Co-first author
*Correspondence: sila.ultanir@nimr.mrc.ac.uk (S.K.U.), yuhnung.jan@ucsf.edu (Y.-N.J.)
http://dx.doi.org/10.1016/j.neuron.2014.10.025

SUMMARY

Mammalian Sterile 20 (Ste20)-like kinase 3 (MST3) is a ubiquitously expressed kinase capable of enhancing axon outgrowth. Whether and how MST3 kinase signaling might regulate development of dendritic filopodia and spine synapses is unknown. Through shRNA-mediated depletion of MST3 and kinase-dead MST3 expression in developing hippocampal cultures, we found that MST3 is necessary for proper filopodia, dendritic spine, and excitatory synapse development. Knockdown of MST3 in layer 2/3 pyramidal neurons via in utero electroporation also reduced spine density in vivo. Using chemical genetics, we discovered thirteen candidate MST3 substrates and identified the phosphorylation sites. Among the identified MST3 substrates, TAO kinases regulate dendritic filopodia and spine development, similar to MST3. Furthermore, using stable isotope labeling by amino acids in culture (SILAC), we show that phosphorylated TAO1/2 associates with Myosin Va and is necessary for its dendritic localization, thus revealing a mechanism for excitatory synapse development in the mammalian CNS.

INTRODUCTION

Dendrite arborization and synapse formation are critical for wiring the neural circuitry (Jan and Jan, 2010; Parrish et al., 2006). Dendrites of pyramidal neurons, the predominant excitatory neurons in the mammalian brain, contain dendritic spines, postsynaptic structures harboring more than 90% of excitatory synapses in the brain (Harris and Kater, 1994; Nimchinsky et al., 2002). Dendritic spine formation is preceded by actin-rich filopodia that typically contain immature synapses and are thought to be involved in dendrite arborization and synaptogenesis (Fiala et al., 1998; Yuste and Bonhoeffer, 2004). Unraveling the molecular mechanisms underlying spine formation is an important research area. Alterations in many proteins implicated in neural development have been linked to neurological disorders, such as autism (Huguet et al., 2013). A better understanding of the molecular mechanisms involved in brain development and synapse formation could enable future therapeutic interventions.

Protein kinases regulate a wide range of cellular processes by phosphorylating and altering the function of their target molecules. There are more than 500 kinases in the human genome. How various kinases regulate neuronal development remains poorly understood. Previous studies by us and others have revealed that the kinase cascade of the Hippo, Wts, and Trc (mammalian nuclear Dbf2-related [NDR] 1/2) kinases play important and evolutionarily conserved roles in dendrite morphogenesis (Emoto et al., 2004, 2006; Gallegos and Bargmann, 2004; Ultanir et al., 2012; Zallen et al., 2000).

Mammalian Sterile 20 (Ste20)-like kinase 3 (MST3) belongs to the highly conserved family of Ste20-like kinases that includes Hippo as the most well studied member. MST3, also known as Serine/threonine kinase 24, is in the subfamily of the germinal centre kinase III kinases that contain an $\sim$275 amino acids long N-terminal kinase domain and a C-terminal regulatory domain. Originally identified as a kinase with requirement of manganese as a preferred cofactor (Schinkmann and Blenis, 1997), MST3 is predominantly localized to the cytoplasm (Preisinger et al., 2004; Schinkmann and Blenis, 1997). MST3a is the shorter 431 amino acid isoform, which differs from the 443 amino acid MST3b in its 16 N-terminal amino acids. MST3 activation can result from autophosphorylation of a threonine in the N-terminal kinase domain (Schinkmann and Blenis, 1997), dephosphorylation of a threonine in the C-terminal regulatory domain to enable binding of the scaffolding protein MO25 (Fuller et al., 2012), or caspase mediated cleavage between these two domains, leading to nuclear localization of the kinase domain (Huang et al., 2002).

MST3 is expressed ubiquitously in various tissues including the brain (Irwin et al., 2006; Schinkmann and Blenis, 1997).

MST3 signaling is involved in hypoxia-induced apoptosis in trophoblasts, where MST3 can activate Caspase 3 (Wu et al., 2008, 2011). MST3b was isolated as a purine-sensitive kinase, which facilitates axon outgrowth in response to inosine (Irwin et al., 2006). MST3b also facilitates axon regeneration in cultures and in vivo (Lorber et al., 2009), and MST3 is required for radial neuronal migration in the developing cortex (Tang et al., 2014). It is unknown whether MST3 plays a role in dendrites, dendritic filopodia, and spine morphogenesis. MST3′s mechanism of action is also an open question.

The homology of MST3 to the Hippo kinase in *Drosophila* prompted us to test whether MST3 signaling has a role in mammalian dendrite development. By inhibiting MST3 activity via expression of a kinase-dead form of MST3 or small hairpin (sh)RNA mediated knockdown of MST3 in dissociated hippocampal neuronal cultures, we show that MST3 is required for the formation and maintenance of dendritic architecture. We found that MST3 limits dendritic filopodia and facilitates the formation of spines harboring mature synapses. In utero electroporation of MST3 shRNA also confirmed that MST3 is required for dendritic spine morphogenesis in vivo. In order to investigate the molecular mechanisms by which MST3 regulates dendritic architecture, we used chemical genetics and mass spectrometry and identified 13 potential substrates of MST3. Next, by expressing phospho mutant and phosphomimetic forms of a subset of these novel substrates, we found that expression of phospho mutant TAO1 or TAO2, or shRNA mediated knockdown of TAO1/2, is sufficient to cause increased dendritic filopodia and loss of spines, reminiscent of MST3 loss of function phenotype. We further show that downstream of TAO1/2, a Myosin Va complex specifically binds to the critical phosphorylation site found on both TAO1 and TAO2. Thus, MST3 kinase functions in the formation of dendritic spines by regulating several downstream effectors. In particular, MST3′s phosphorylation of TAO1/2, an autism spectrum disorder susceptibility gene, is critical for its regulation of Myosin Va dependent trafficking in dendrites.

RESULTS

MST3 Limits Filopodia on Developing Dendrites

Localization of endogenous MST3 in punctate structures in the neuronal soma and dendrites was evident from immunofluorescence staining of dissociated hippocampal neurons double labeled with the dendrite marker microtubule-associated protein 2 (MAP2) (Figure 1A), as well as immunofluorescence staining of GFP transfected neurons in high density cultures with monoclonal antibody against MST3 (Figure 1B). The immunofluorescence staining was dramatically reduced by shRNA knockdown of MST3, indicating the staining is specific to MST3 kinase (Figures S1A and S1B available online). Similar cytoplasmic distribution of Hemagglutinin (HA)-tagged MST3 proteins expressed in hippocampal neurons could be detected by anti-HA immunocytochemistry (Figure S1G).

To find out if MST3 is required for postnatal dendritic development, we transfected neurons at 3 days in vitro (DIV3) with GFP plasmid alone as control, or a plasmid with GFP and MST3 shRNA expressed via separate promoters, or mutant MST3 and GFP expressing plasmids. GFP expressing control neurons at DIV7 displayed dendritic filopodia (Figure 1C). Inhibiting MST3 function by expressing either MST3 shRNA or MST3 kinase dead (K53R) mutant (MST3-KD) together with GFP resulted in a dramatic increase in the total dendritic filopodia length per 50 μm dendrite and filopodia density (Figures 1C and 1D), as well as average filopodia length (Figure S1C). As expected for effects due to loss of MST3 function, these phenotypes could be rescued by coexpressing MST3 shRNA with shRNA-resistant MST3 (MST3*) (Figures 1C, 1D, and S1C), and they could be produced by two different MST3 shRNAs (Figure S1H) that effectively knocked down endogenous MST3 (Figures S1F and S4F), but not by expression of MST3* or a control shRNA (Figures S1D and S1E). It is conceivable that MST3-KD might be acting as a dominant-negative form for MST3 function via binding and titrating coactivators. These data indicate that MST3 is required in developing dendrites to limit dendritic filopodia formation during early postnatal development.

MST3 Is Required for Spine Formation and Maintenance

To test if MST3 is also required later in development for the formation or maintenance of dendritic spines, we transfected neurons with shRNA constructs at DIV16 when spine formation has largely taken place, and observed spines at DIV20. MST3 shRNA expression resulted in a robust reduction of dendritic spines and an increase in dendritic filopodia, which could be rescued by expressing MST3* (Figures 2A and 2B). Moreover, coexpression of MST3 shRNA together with the postsynaptic marker PSD-95-GFP and Td-tomato led to a reduction of the density of PSD-95-GFP containing dendritic protrusions (Figures 2C and 2D), while total PSD-95-GFP intensity in dendrites and spines remained constant (data not shown). The density of dendritic protrusions that colocalized with endogenous synapsin or PSD-95 was reduced in MST3 shRNA expressing neurons as well (Figures S2A and S2B). These data indicate that MST3 is required for maintaining the structure of dendritic spines and excitatory synapses in cultures.

To test whether MST3 alters spine morphogenesis in vivo, we expressed MST3 shRNA in developing layer 2/3 cortical neurons in mice via in utero electroporation at embryonic day (E) 14.5 in mice (Figure 2E). We found no change in spine head diameter (Figure 2G), but a significant reduction in spine density in MST3 shRNA expressing neurons compared to control neurons expressing GFP alone (Figures 2E and 2F). Taken together, these findings indicate MST3 kinase signaling is important for the formation and maintenance of spines and excitatory synapses.

Identification of MST3 Phosphorylation Substrates Using Chemical Genetics

To identify downstream effectors of MST3, we used a chemical genetic method for kinase substrate identification (Blethrow et al., 2004; Hertz et al., 2010) used previously to identify the substrates of NDR 1/2 (Ultanir et al., 2012). We generated two ATP-analog sensitive MST3 mutants, MST3 M99G and MST3 M99A, by mutating the “gatekeeper” residue at the

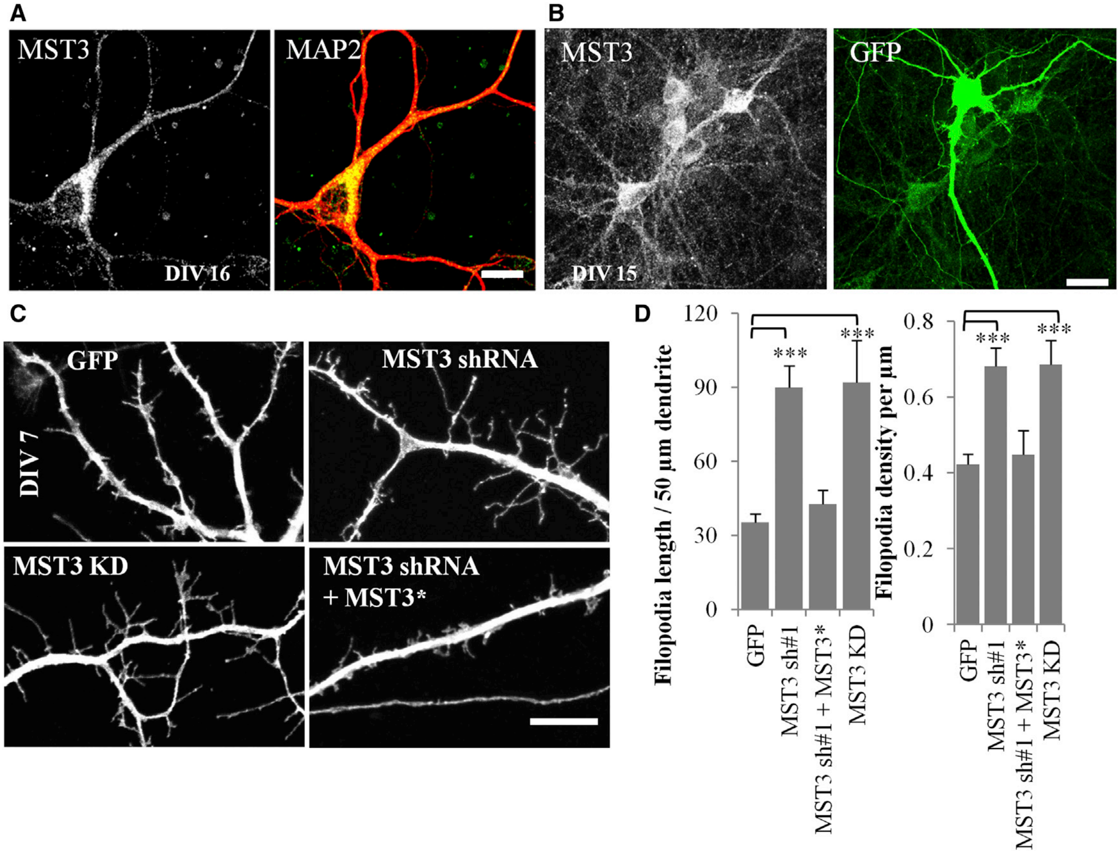

Figure 1. MST3 Is Required for Limiting Dendritic Filopodia in Hippocampal Cultures

(A) Image shows MAP2 and MST3 immunostaining of a DIV16 hippocampal neuron; MST3 is found in the cell body and dendrites. Scale bar represents 10 μm.

(B) Hippocampal cultures are transfected with GFP and immunostained with a monoclonal MST3 antibody generated in mouse. Scale bar represents 25 μm.

(C) Representative images of GFP expressing hippocampal neuron dendrites at DIV7. Confocal stacks were obtained and projected at maximum intensity. Scale bar represents 7.5 μm.

(D) MST3 shRNA and MST3 KD expression leads to increased filopodia length per 50 μm of dendrite and filopodia density, and shRNA effects can be rescued with shRNA resistant MST3 (MST3*) (N = 14, 15, 11, and 6, respectively in columns in graphs). In all figures * = $p < 0.05$, ** = $p < 0.01$, and *** = $p < 0.001$, in Student's t test. All error bars reflect SEM. See also Figure S1.

ATP binding pocket to allow for utilization of bulkier ATP analogs (Blethrow et al., 2004). In order to determine if the MST3 analog-sensitive mutants can utilize a bulky ATP analog with high efficiency, we used the autophosphorylation activity of MST3 as a readout in kinase assays using ATP-γ-S, Benzyl-ATP-γ-S, Furfuryl-ATP-γ-S, or Phenyl-Ethyl-ATP-γ-S (Figure 3A). Thiophosphorylated substrate, in this case MST3 itself, was alkylated by p-nitrobenzylmesylate (PNBM) for detection via an anti-thiophosphate ester antibody (Allen et al., 2007). Because the MST3 M99G analog sensitive kinase was active with a preference toward Benzyl-ATP-γ-S, we used this combination to phosphorylate and then detect MST3 substrates present in the brain.

To undertake covalent capture for kinase substrate identification (Blethrow et al., 2008; Hengeveld et al., 2012; Ultanir et al., 2012) (Figure 3B), we purified HA tagged MST3 M99G and MST3-KD that were expressed in COS-7 cells via immunoprecipitation using HA antibody bound-beads (Figure S3A) and labeled brain lysates from postnatal day (P) 8–9 mice with Benzyl-ATP-γ-S, with or without MST3 M99G or MST3-KD, for 1–2 hr at 30°C. For purification of thiophosphorylated peptide fragments of potential MST3 targets using the three-step covalent capture protocol, (1) proteins in the brain lysate were digested into tryptic fragments, (2) Thiol containing peptides (including those containing cysteine amino acids) were captured onto iodoacetyl agarose beads, and (3) thiophosphate ester linked peptides were released from the beads by an oxone induced hydrolysis reaction as phospho-peptides, while cysteine linked peptides remained on beads. Eluted peptides and phosphorylation sites were identified in liquid chromatography/tandem mass spectrometry analysis. Phospho-peptides detected more than once in MST3 M99G experimental samples and not in any of the negative controls are included in a list of candidates (Figure 3B). Examples of mass spectra for identified MST3 substrates TAO1 (Figure 3C), EPS8, and TAO2 (Figures S3B and S3C) are shown.

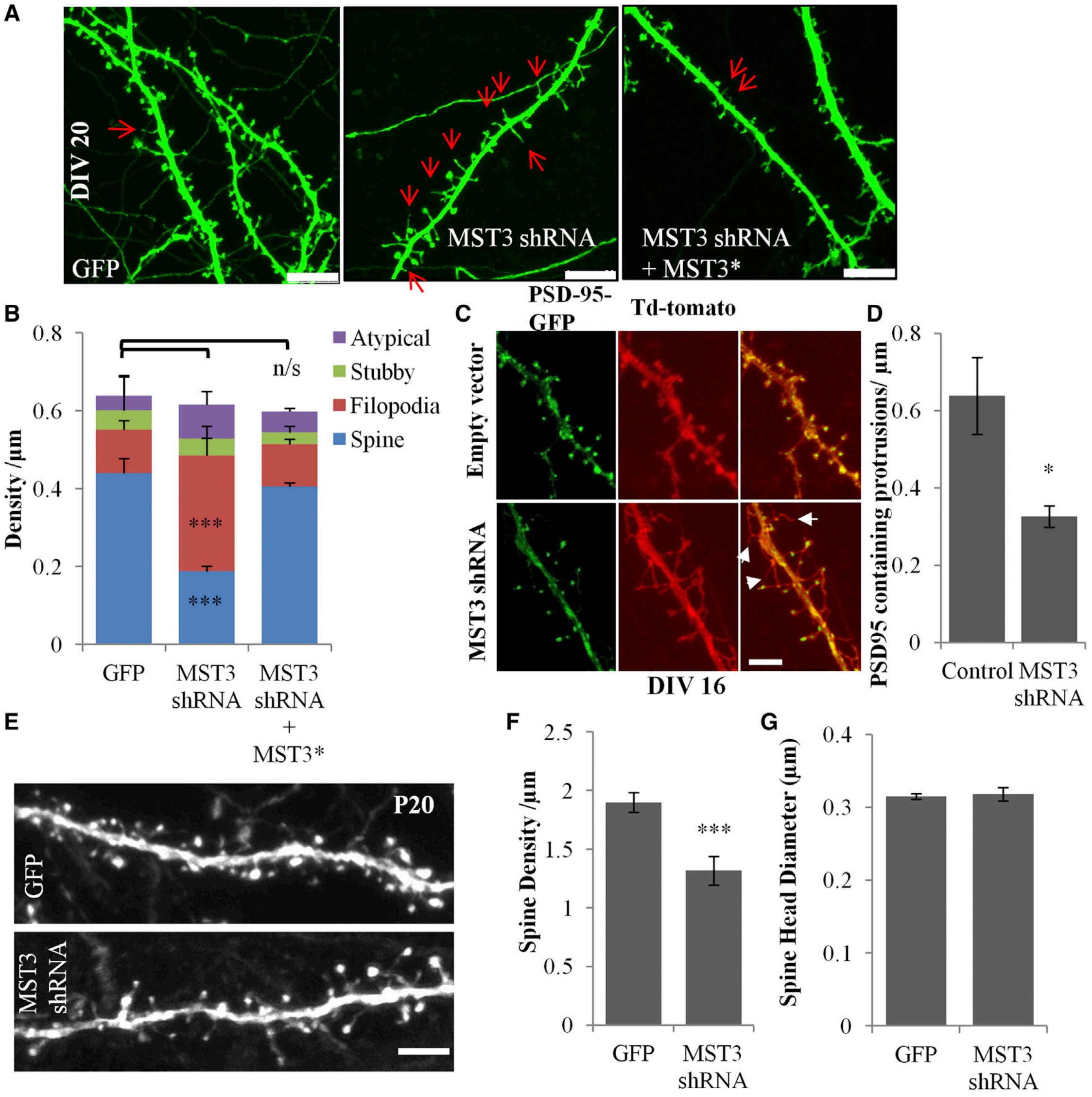

Figure 2. MST3 Is Required for Spine Development in Hippocampal Cultures and In Vivo

(A) Hippocampal neurons were transfected at DIV16 and imaged at DIV20. Scale bar represents 7.5 µm.

(B) Filopodia density is increased and spine density is reduced in MST3 shRNA expressing neurons. The effect was rescued with shRNA resistant MST3* expression (n of cells = 11, 12, and 6, respectively).

(C and D) Hippocampal neurons were transfected with PSD-95-GFP and Td-tomato alone or Td-tomato together with MST3 shRNA at DIV13 and fixed and imaged at DIV16. Density of protrusions containing PSD-95-GFP puncta is reduced in MST3 shRNA expressing neurons (n of cells = 5 for each condition). Arrows point to filopodia that do not contain PSD-95. Scale bar represents 5 µm. E. MST3 shRNA or GFP alone is expressed in layer 2/3 neurons via in utero electroporations at E14.5. Brain sections are analyzed at P20. Representative basal dendrite images of layer 2/3 neurons expressing GFP. Scale bar represents 3 µm.

(F and G) Spine density is reduced and spine head diameter is not changed in MST3 shRNA expressing neurons' basal dendrites (n of cells = 2 and 7, respectively). All error bars reflect SEM. See also Figure S2.

Several MST3 Phosphorylation Substrates Are Cytoskeleton Regulators

Of 13 potential MST3 substrates identified along with their phosphorylation sites (Table 1), tubulin polymerization-promoting protein (TPPP) and Ermin are specific to oligodendrocytes (Brockschnieder et al., 2006; Song et al., 2007), and the other 11 proteins are potential neuronal targets, including EPS8, an actin capping protein; TAO1 and TAO2, microtubule and actin interactor binding proteins; FMNL2, an actin binding protein, Arhgap8, a Rho-GTPase activator; and GIPC and Tsg101 that function in vesicle trafficking. Another candidate MST3 substrate is Caspase 3 involved in the elimination of synapses in neurons (D'Amelio et al., 2012).

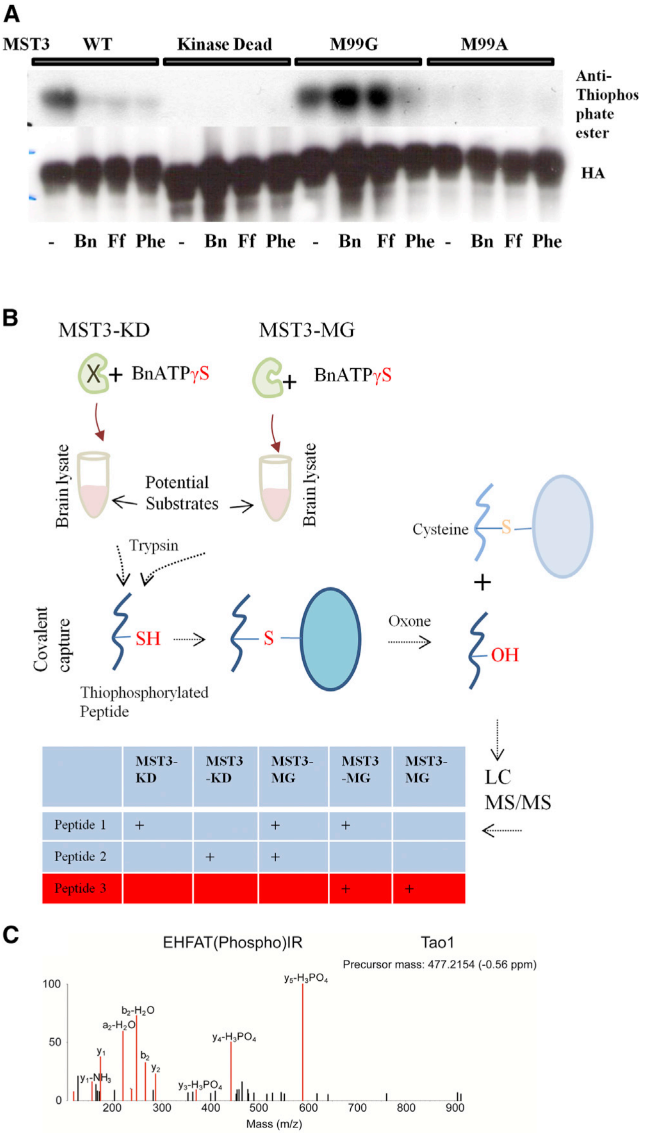

Figure 3. MST3 Substrate Identification Using Chemical Genetics

(A) MST3-MG can use Benzyl-ATP-γ−S to autophosphorylate. HA tagged MST3 wt, KD, and analog sensitive mutants (MG and MA) purified from HEK293 cells and subjected to kinase assay using ATP-γ-S analogs. Autophosphorylation of HA-MST3 is used as a readout of MST3 activity in kinase assays conducted with various ATP-γ-S analogs: Benzyl-ATP-γ-S (Bn), Furfuryl-ATP-γ-S (Ff), and Phenyl-ethyl-ATP-γ-S (Phe). Total MST3 levels are shown by anti-HA blot. MST3 M99G can use Bn efficiently. Kinase assay is detected using PNBM alkylation and anti-thiophosphate ester blot.

(B) Simplified scheme of kinase substrate labeling and identification (Hertz et al., 2010).

(C) MS spectra shows the detected phosphopeptide that belongs to the substrate TAO1. See also Figure S3.

This study further uncovered a phosphorylation consensus sequence for MST3: threonine (as opposed to serine) is the phosphorylated amino acid followed by Isoleucine, Leucine, or Valine. In about half of the substrates that we identified, an Arginine was found at +2, resulting in a consensus site of T*− I/L/V−R for phosphorylation by MST3.

Table 1. Putative MST3 Substrates Are Listed

	Candidate	# /4 Exps	Phosphorylation Site		Function
1	EPS8	3	T317	PGE[a]GVLT*L[a]R[a]	actin capping
2	TAO1	2	T440	NRE[a]HFAT*I[a]R[a]	microtubule stability
3	TAO2	2	T475	NRE[a]HFAT*I[a]R[a]	dendrite and spine development
4	FMNL2	2	T202	SALRYNT*L[a]P	actin bundle formation
5	Arhgap18	3	T156	VETVSQT*L[a]R[a]	actin regulation via Rho GTPase
6	Pak6	3	T99	SVISSNT*I[a]R[a]	rhoGTPase effector
7	GIPC	2	T242	LGTGRGT*L[a]R[a]	NMDA receptor trafficking
8	EF1A1	3	T432	VRD[a]MRQT*V[a]A	translation elongation
9	Caspase-3	2	T20	NNFEVKT*I[a]H	synaptic plasticity, dendrite pruning
10	Tsg101	3	T221	GPSRDGT*I[a]S	membrane transport
11	Cystine glutamate transporter	3	T9	SAVSSPT*V[a]	transporter
12	Ermin	2	T257	SYSRYNT*I[a]S	actin regulation
13	TPPP	2	T161	RKPVVAT*I[a]S	microtubule regulation

The number of times a protein is identified out of four MS experiments is shown in the third column. Phosphorylated amino acid number and the phosphorylation sites are shown in the fourth and fifth columns. Brief descriptions of reported functions of putative substrates are listed in the last column.

[a]Indicates the phosphorylation consensus motif that emerged from our screen.

Thousand and One Amino Acid1/2 Phosphorylation by MST3 Is Important for Limiting Filopodia and Maintaining Spines

To find out whether some of the putative MST3 substrates modulate filopodia and spine morphogenesis, we tested phospho mutants and phospho-mimetic mutants of TAO1, GIPC, ARHGAP18, FMNL2, EPS8, and PAK6 by expressing HA tagged mutant substrates in hippocampal neurons at DIV3, confirming their expression by HA immunostaining (Figure S4A) and assessing filopodia phenotypes at DIV7–8. Of the six substrates tested, the EPS8 phospho-mimetic form (T317D) caused reduction of filopodia density, while the TAO1 phospho mutant (T440A or TAO1A) increased filopodia density per dendrite length and total filopodia length per 50 μm dendrite, similar to the loss of function of MST3 phenotype (Figures 4A, 4B, and S4B). The TAO1 T440D mutant (TAO1D) or wild-type TAO1 expression did not alter filopodia structure or density (Figures 4A, S4B, and S4C). While this experiment does not rule out roles of other identified downstream effectors, it shows that TAO1

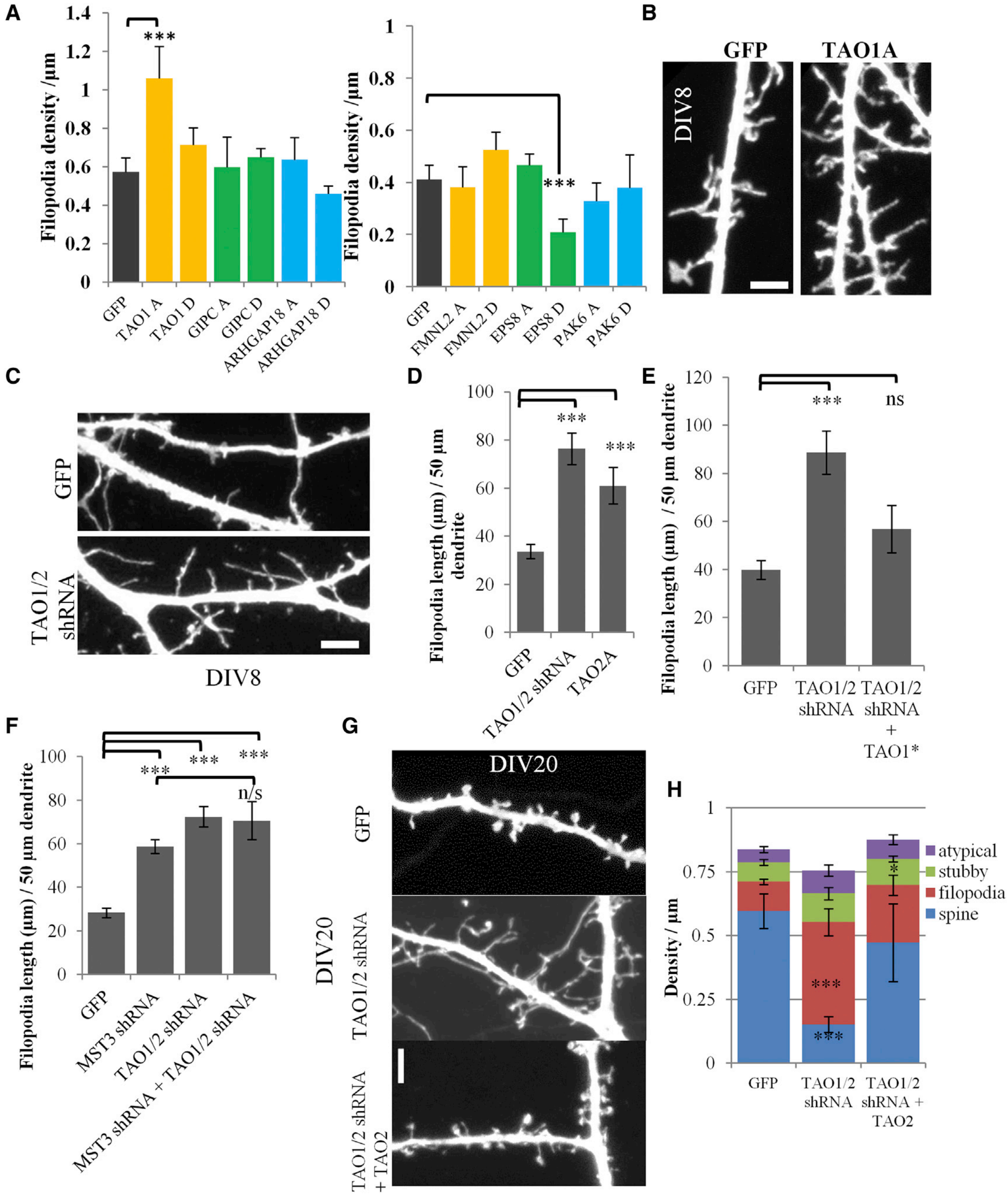

Figure 4. Effects of Phosphomutant and Phosphomimetic Substrate Expression on Dendritic Filopodia in Hippocampal Neurons

(A) There were six putative MST3 substrates TAO1, GIPC, ArhGAP18, FMNL2, EPS8, and PAK6 that were mutated at their MST3 phosphorylation site to produce phosphomutant (T > A, e.g., TAO1A) or phosphomimetic (T > D, e.g., TAO1D) mutants. Filopodia density is measured in neurons transfected at DIV3 and imaged at DIV7 (For left graph n = 6, 6, 6, 6, 5, 4, and 5 cells, for right graph n = 6, 8, 6, 8, 9, 5, and 5 cells, respectively).

(B) Representative examples of dendrites of neurons expressing GFP alone and GFP and TAO1A mutant. Scale bar represents 2.5 μm.

(C) Representative images of dendrite expressing GFP alone and GFP and TAO1/2 shRNA. Scale bar represents 2.5 μm.

(D) TAO1/2 shRNA and TAO2A significantly increases filopodia length per 50 μm of dendrite (n = 14, 14, and 6 cells, respectively).

(E) Increase in filopodia by TAO1/2 shRNA can be rescued by TAO1* resistant to TAO1/2 shRNA (n = 7, 9, and 9 cells, respectively).

(F) Total filopodia length per 50 μm dendrite is increased in MST3 shRNA and TAO1/2 shRNA expressing neurons. MST3 and TAO1/2 shRNA knockdown together does not further increase filopodia (n = 5, 7, 8, and 8 cells, respectively).

(G) Images of dendritic spines of neurons transfected with GFP, TAO1/2 shRNA, and TAO1/2 shRNA cotransfected with human TAO2 construct, which is insensitive to this shRNA, are shown. Scale bar represents 3 μm.

(H) Dendritic filopodia density is increased and spine density is reduced significantly upon TAO1/2 knockdown. The effect was mitigated by expression of human TAO2 together with TAO1/2 shRNA (n = 10, 10, and 9 cells, respectively). All error bars reflect SEM. See also Figure S4.

and TAO2 kinase phosphorylation by MST3 is critical for limiting dendritic filopodia.

Next, we tested knockdown of TAO1 or EPS8 by generating three shRNAs in each case. Expressing shRNAs targeting TAO1 alone (not TAO2) or EPS8 between DIV3 and DIV7–8 did not alter filopodia of hippocampal neurons (Figures S4D and S4E, and data not shown), notwithstanding the ability of EPS8 to limit filopodia (Menna et al., 2013; Stamatakou et al., 2013) and the effect of the EPS8 phospho-mimetic form in reducing filopodia density (Figure 4A). It is conceivable that the EPS8 function could be compensated for by other isoforms such as EPS8L2 and EPS8L3 that also contain the MST3 phosphorylation site.

A TAO2 targeting shRNA with a one base pair mismatch with TAO1 knocked down TAO2 together with TAO1 so is referred to as TAO1/2 shRNA (Figure S4F). Transfection at DIV3 with TAO1/2 shRNA or TAO2 phospho mutant (T475A) caused a robust increase at DIV7–8 in dendritic filopodia, similar to the MST3 loss of function phenotype (Figures 4C, 4D, and S4G), and this phenotype can be rescued with shRNA-resistant mouse TAO1* (Figure 4E). There were two additional shRNAs targeting TAO2 only that did not yield this phenotype (data not shown). Expression of both MST3 shRNA and TAO1/2 shRNA increased the total filopodial length per 50 μm dendrite to a similar extent as MST3 knockdown (Figure 4F), indicating that MST3 and TAO1/2 are acting on the same pathway.

To test for TAO1/2 function in the spine maintenance, we transfected cultured neurons with TAO1/2 shRNA at DIV16 and imaged these neurons at DIV20. TAO1/2 shRNA caused a reduction in dendritic spines and an increase in filopodia, which was rescued by shRNA-resistant human TAO2 (Figures 4G and 4H). Expression of PSD-95-GFP with Td-tomato and TAO1/2 shRNA between DIV13 and DIV16 revealed a reduction in the density of protrusions containing PSD-95-GFP (Figures S4H and S4I), implicating TAO1/2 in spine synapse maintenance as well as filopodia development.

MST3 Is Necessary and Sufficient for Thousand and One Amino Acid1/2 T440/T475 Phosphorylation

We raised a phospho-specific antibody (pTAO1/2) against the phosphorylation site T440/T475 on TAO1/TAO2, which is almost identical in TAO1 and TAO2, cotransfected MST3-HA with wild-type TAO2 or T475A mutant TAO2 (TAO2A), and found MST3-dependent phosphorylation of the wild-type, but not mutant TAO2 (Figure 5A). To test if MST3 is necessary for TAO2 phosphorylation, we transfected human embryonic kidney 293 (HEK293) cells with shRNAs targeting human MST3 along with wild-type TAO2 or TAO2 T475A mutant. MST3 knockdown reduced the levels of baseline phosphoTAO1/2, but not total TAO2 protein (Figure 5B). Similar results were obtained for TAO1 (data not shown), indicating MST3 is necessary and sufficient for TAO1/2 phosphorylation.

Next, we transfected hippocampal neurons at DIV13 with control shRNA or MST3 shRNA and stained these neurons at DIV16 with phosphoTAO1/2 antibody, after verifying its specificity by showing reduced immunostaining upon TAO1/2 shRNA expression (Figure S5A). MST3 knockdown reduced phosphoTAO1/2 levels in the soma (Figures 5C and 5D). Immunostaining further indicated that MST3 phosphorylation of TAO1/2 could take place in the cell body and/or dendrites (Figure 5E).

Thousand and one amino acid1/2 (TAO1/2) kinases are Ste20-like kinases implicated in microtubule and actin regulation (see Discussion). TAO1 and TAO2 are expressed from postnatal day (P)5 to P15 and then downregulated at P20, although TAO1 expression was still detectable (Figure 5F). We next investigated their role in spine development in vivo by performing in utero electroporations at embryonic day (E)14.5 either with GFP plasmid alone as control or together with MST3 shRNA or TAO1/2 shRNA, and imaging dendritic spines of perfusion fixed brains at P20. Similar to MST3 shRNA, TAO1/2 shRNA caused reduction in dendritic spine density in vivo (Figures 5G and 5H).

A Myosin-Va Containing Complex Binds Thousand and One Amino Acid1/2 Phosphorylation Site in a Phosphorylation Dependent Manner

To test if the phosphorylated TAO1/2 site is involved in phosphorylation–dependent protein interaction, we used a peptide pull-down method to identify binding proteins in neuronal lysates labeled by stable isotope labeling by amino acids in culture (SILAC) (Spellman et al., 2008; Stephanowitz et al., 2012; Zhang et al., 2011).

We incubated iodoacetyl agarose beads conjugated with peptides corresponding to the TAO2-T475 phosphorylation site in either nonphosphorylated or phosphorylated forms (Figure 6A) with neuronal lysates labeled with medium heavy (Lys4 Arg6) and heavy (Lys8 Arg10) amino acids, and subjected the proteins bound to the peptide-coated beads to trypsin digest and mass spectrometry. The relative abundance of each protein was calculated based on the median of the medium heavy/heavy ratios for all peptides identified for that protein. A majority of proteins associated with both peptide- and phospho-peptide conjugated beads (diamonds in Figure 6A), whereas a number of proteins were enriched in the phospho-peptide conjugated beads. We repeated this experiment by switching the medium heavy and heavy labels on peptide/phospho-peptide pull-downs and plotted results from the first and second experiments on the x and y axis. The numbers represent the base 2 logarithms of the ratio of medium heavy/heavy forms of each detected protein (Figure 6B), so that the top left quadrant contains proteins that are enriched in phospho-peptide pull-down in both experiments (Table S1). We found that Myosin Va, Myosin light chain 6, and DrebrinE bound to the phospho-peptide with higher efficiency than nonphosphorylated peptide (Figure 6B).

To verify the phospho-specific binding between the TAO1/2 T440/T475 site and Myosin Va, we incubated lysates from HEK293 cells expressing GFP alone or GFP tagged Myosin Va-globular tail domain (GTD) with lysates from cells expressing either TAO1-HA or TAO1-T440A-HA. Immunoprecipitation using an anti-GFP antibody followed with western blot of the bound proteins with HA antibody revealed that TAO1-HA, but not TAO1-T440A-HA could bind Myosin Va (Figure 6C). Similar coimmunoprecipitation experiments using Drebrin-GFP did not confirm binding between TAO1-HA and Drebrin-GFP (data not shown). To test if endogenous TAO1 or TAO2 can bind endogenous Myosin Va, we immunoprecipitated Myosin Va from

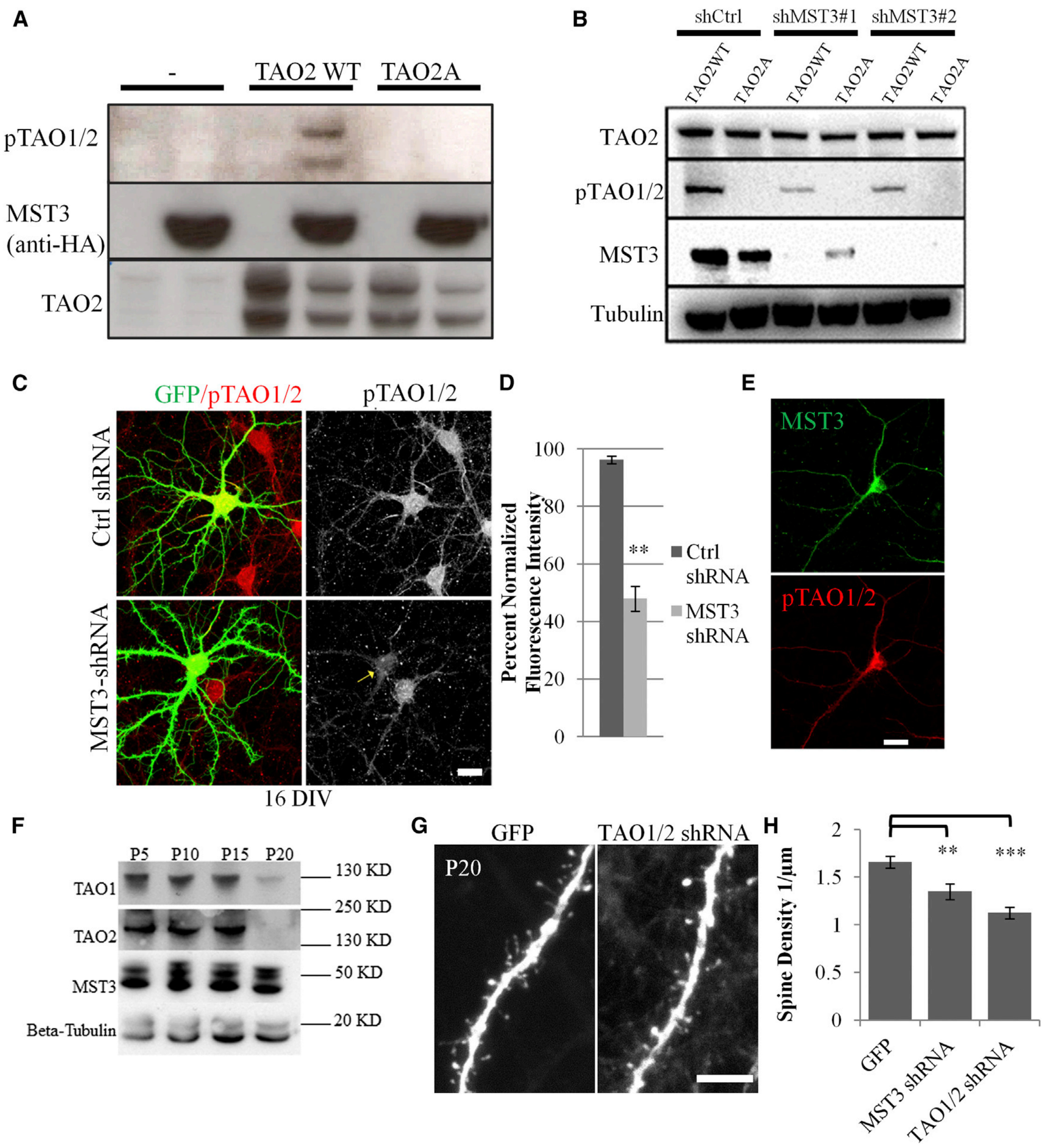

Figure 5. MST3 Is Necessary and Sufficient to Phosphorylate Thousand and One Amino Acid1/2 at T440/475

(A) pTAO1/2 signal is increased when MST3-HA is coexpressed with wild-type TAO2, but not with TAO2-A (T475A). Expressed TAO2 migrates at two separate bands at ~140 kDa and ~200 kDa in this experiment, although the expected size is 140 kDa.

(B) TAO2 WT or TAO2A is transfected in HEK293T cells together with MST3 shRNAs targeting human MST3 or a control shRNA. TAO2 phosphorylation at T475 is reduced when MST3 is knocked down in lanes three and five when compared to control in lane one, suggesting that MST3 is necessary for T475 phosphorylation. As expected, pTAO1/2 antibody does not detect any signal when TAO2A is expressed in lanes two, four, and six.

(C) MST3 shRNA causes reduction of phosphorylated TAO1/2 440/475. Hippocampal neurons expressing control shRNA or MST3shRNA with GFP are stained with phosphoTAO1/2 antibody (red). Scale bar represents 20 μm.

(D) Quantification of phosphorylated TAO1/2 in cell body is significantly reduced in MST3 shRNA (n = 3 experiments each).

(E) MST3 and phosphoTAO1/2 440/475 costaining is shown. Scale bar represents 20 μm.

(F) Expression of TAO1, TAO2, and MST3 in brain lysates during development.

(G) TAO1/2 shRNA expressing layer 2/3 neuron basal dendrites in vivo (via in utero electroporation) at P20 is shown. Scale bar represents 5 μm.

(H) Quantification of spine density in neurons expressing GFP, MST3 shRNA, and TAO1/2 shRNA (n = 4, 3, and 3 animals and 19, 14, and 13 neurons, respectively). All error bars reflect SEM. See also Figure S5.

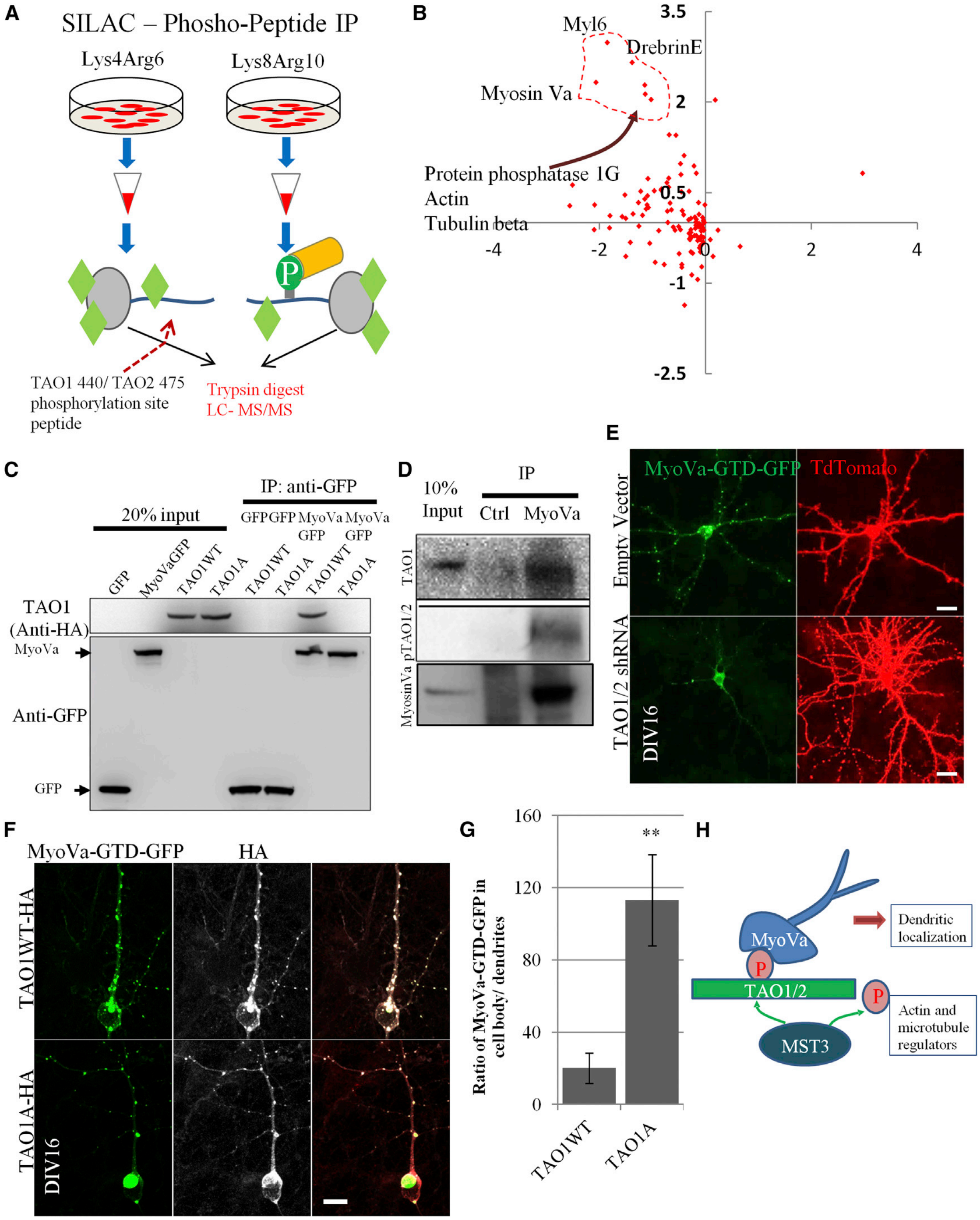

Figure 6. Myosin Va Interacts with Thousand and One Amino Acid1/2 upon Its Phosphorylation by MST3

(A) Schematic representation of SILAC experiment with peptide pull-down. Hippocampal neurons were cultured in SILAC media containing either medium heavy (Lys4 Arg6) or heavy (Lys8 Arg10) amino acids from plating to DIV9 when they were harvested. Protein lysates were incubated with beads attached with 17 amino acids long peptides containing TAO1/2′s MST3 phosphorylation site, either in phosphorylated and nonphosphorylated forms. Proteins bound to the peptides were digested and identified in MS.

(B) There were two separate experiments where SILAC labels that were switched are plotted with respect to each other. Higher values in positive y axes and negative x axes represent enriched binding of proteins to phospho-peptide. The proteins enriched at values >1 in both experiments are circled by a dotted line. Myosin Va was enriched >2 in both experiments (see Table S1 for all values).

(C) GFP immunoprecipitation of Myosin Va-GTD-GFP, labeled as Myosin Va-GFP, after incubation with lysates expressing TAO1-HA wild-type and TAO1-HA T440A mutants, reveal that only TAO1WT and not TAO1A can bind (coimmunoprecipitate) Myosin Va-GTD-GFP.

(legend continued on next page)

dissociated hippocampal neurons at DIV6 and found that Myosin Va can bind TAO1 and phosphoTAO1/2 specifically (Figure 6D), while we were not able to detect a signal with TAO2 antibody (data not shown). Thus, MST3 phosphorylates TAO1 and TAO2, enabling their association with Myosin Va.

Myosin Va Recruitment to Dendrites Is Enhanced by Thousand and One Amino Acid1/2

Myosin V has been implicated in spine morphology and α-Amino-3-hydroxy-5-methyl-4-isoxazolepropionic acid (AMPA) type glutamate receptor trafficking (Correia et al., 2008; Lisé et al., 2006; Wang et al., 2008). To test for TAO1/2 involvement in Myosin Va function, we transfected cortical neurons at DIV13 with GFP tagged Myosin Va-GTD, a second plasmid (pLentiLox 3.7) coexpressing membrane tagged Td-tomato to visualize neuronal membrane, and shRNA targeting TAO1/2, and imaged at DIV16 after fixation. Myosin Va-GTD-GFP accumulated in the dendrites in punctate pattern (Figure 6E, top row, n = 8 neurons). Upon TAO1/2 shRNA expression, the dendritic Myosin Va-GTD-GFP distribution was disrupted in nine out of ten neurons (Figure 6E, bottom row) ($p < 0.001$ chi-square). Myosin Va-GTD-GFP can still be localized to axons in TAO1/2 shRNA neurons (Figure S6F), indicating that dendritic localization is specifically altered in the absence of TAO1/2.

To test if TAO1/2 knockdown affects specifically Myosin Va, we transfected cortical neurons with HA tagged Myosin Va-GTD or HA tagged Myosin Vb-GTD together with vector expressing GFP alone or GFP with TAO1/2 shRNA (Figure S6), and immunostained these neurons with an anti-HA antibody (red). Myosin Va-HA was found in dendrites in eight out of ten neurons coexpressing GFP, whereas TAO1/2 knockdown in all 11 neurons reduced Myosin Va in dendrites (Figures S6A and S6C, $p < 0.001$ chi-square). Unlike Myosin Va, Myosin Vb-GTD puncta accumulated in the perinuclear region of control neurons (Figure S6B, arrow, n = 12 neurons). Interestingly, TAO1/2 knockdown caused Myosin Vb-HA puncta to appear in dendrites in 12 out of 13 neurons (Figures S6B and S6D, $p < 0.001$ chi-square). Thus, TAO1/2 facilitates dendritic localization of Myosin Va, and knockdown of TAO1/2 results in an increase of Myosin Vb in dendrites, possibly via a compensatory upregulation. Moreover, Myosin Va-GTD-GFP puncta colocalized with endogenous Rab11 (Figure S6E), indicating that Myosin Va could be recruited to recycling endosomes, where its function could be required for dendritic spine formation.

To find out if the T440 residue of TAO1 is important for the dendritic localization of Myosin Va-GTD-GFP, we expressed TAO1wild-type (WT) or TAO1 T440A mutant together with Myosin Va-GTD-GFP in dissociated cortical neurons at DIV13 and imaged at DIV16. TAO1WT and TAO1A colocalized with Myosin Va-GTD-GFP (Figure 6F), in contrast to the diffuse cytoplasmic staining in GFP expressing control neurons (Figure S4A). By taking the ratio of the normalized Myosin Va-GTD-GFP signal in the cell body and that in a primary dendrite, we found that Myosin Va-GTD-GFP is enriched in the cell body in TAO1A expressing neurons when compared to TAO1WT expressing neurons (Figure 6G, n = 7 and 8 neurons in TAO1WT and TAO1A, respectively). Thus, while there might be other proteins that enable Myosin Va and TAO1 to be found in the same complex, T440 phosphorylation is critical in enabling dendritic localization of a larger percentage of Myosin Va. In summary, not only does Myosin Va associate with TAO1/2 kinase, but its localization is also altered by TAO1/2 function and phosphorylation state, which in turn is dependent on MST3 kinase, as depicted in Figure 6H.

DISCUSSION

In this study, we examine MST3 kinase signaling by identifying multiple potential substrates, mostly cytoskeletal regulators. In particular, the TAO1/2 kinase upon phosphorylation by MST3 on site T440/T475 associates with Myosin Va in a phosphorylation dependent manner, and this phosphorylation promotes Myosin Va recruitment in dendrites. Thus by using chemical genetic techniques, we have uncovered a kinase signaling cascade that functions in neuronal dendritic spine and synapse formation.

Role of MST3 in Dendrite, Dendritic Filopodia, and Spine Development In Vitro and In Vivo

By expressing MST3 shRNA or KD MST3 in cultured hippocampal neurons, we show MST3 is required for limiting dendritic filopodia and maintaining dendritic spine and excitatory synapse structure. Whereas the MST3 shRNAs used in our study target regions common to MST3a and MST3b, expression of the

(D) Endogeneous Myosin Va is immunoprecipitated using an antibody from dissociated hippocampal neurons protein lysates (at DIV6 or DIV9). Immunoblots show that endogeneous TAO1 and phosphoTAO1/2 are immunoprecipitated by endogeneous Myosin Va.

(E) MyosinVa-GTD-GFP distribution in dendrites is altered upon TAO1/2 shRNA expression in cortical neurons. Myosin Va-GTD-GFP is imaged by confocal microscopy without amplification via immunostaining. Z projections are shown. Td-tomato is expressed pLentiLox3.7 (empty vector) in which GFP is replaced by Td-tomato. TAO1/2 shRNA is also expressed on the same plasmid at a different promoter. Images are captured with same settings. N = 8 out of 8 neurons expressing empty vector showed the showed punctate Myosin Va-GTD-GFP in dendritic arbor. Neurons expressing TAO1/2 shRNA lacked dendritic distribution as depicted in lower panel n = 9 out of 10. Scale bars represent 25 μm.

(F) Myosin Va-GTD-GFP is coexpressed with either wild-type or T440A mutant TAO1-HA in cortical neurons. TAO1 (HA staining) is colocalized with Myosin Va-GTD-GFP (arrows). Scale bar represents 10 μm.

(G) Myosin Va-GTD-GFP accumulation in cell body when compared to dendritic Myosin Va was lower in TAO1-WT expressing neurons than in TAO1-A expressing neurons. Total cell body Myosin Va-GTD-GFP intensity is measured in z projected images and normalized per area after background subtraction. Dendritic puncta were outlined individually, total intensity was measured and normalized to total puncta area and dendritic length (~30 μm dendrite was included). Ratio between cell body/dendritic Myosin Va intensity is shown for TAO1-wt and TAO1-A (n = 7 and 8 neurons, respectively) ($p < 0.01$).

(H) Signaling pathway identified. MST3 phosphorylates several substrates particularly actin and microtubule binding/regulating proteins. MST3 phosphorylates TAO1/2, upon phosphorylation Myosin Va containing complex can bind TAO1/2. TAO1/2 function is critical for Myosin Va localization to dendrites. All error bars reflect SEM. See also Figure S6.

shorter MST3a isoform is sufficient for rescuing the shRNA effects. MST3b has distinct functions in axonal growth, which could be due to possible specific subcellular localization or interactions. Using pan-MST3 antibody raised against a common epitope of MST3a and MST3b, we observed a mostly dendritic and perinuclear staining for MST3 (Figure 1), which overlaps with phosphoTAO1/2 staining (Figure 5E) and supports their function for dendritic filopodia and spine development. MST3 is also implicated in radial neuronal migration (Tang et al., 2014). Our in vivo analyses using in utero electroporations at E14.5 showed a majority of electroporated neurons in upper layers at P20. Hence, we focused on spine development on layer 2/3 neuron basal dendrites.

MST3′s Phosphorylation Substrates

Notwithstanding its broad expression pattern and its functions in apoptosis and axonal growth, little is known about the phosphorylation targets of MST3 except for NDR1 kinase (Stegert et al., 2005). By using the covalent capture method as an unbiased screen that can use complex protein mixtures as the source of substrates and can also identify the phosphorylation site (Hertz et al., 2010; Ultanir et al., 2012), we identified 13 phosphorylation targets of MST3, as well as a consensus motif for MST3 phosphorylation.

Several of the candidate MST3 substrates identified in this study implicate MST3 in regulating the cytoskeleton (Table 1). Among these candidates, we chose TAO1/2 for further study (see next section), and the rest are briefly discussed here. EPS8 is an actin capping protein (Menna et al., 2009) originally identified as a phosphorylation target of the epidermal growth factor receptor. Interestingly, EPS8 positively regulates spine formation and maturation and negatively regulates filopodia (Menna et al., 2013; Stamatakou et al., 2013), in agreement with our finding that the EPS8 phospho-mimetic form reduces filopodia formation. The lack of effect of EPS8 shRNA in hippocampal neuronal cultures could be due to the potential presence of its homologs EPS8L, EPS8L2, and EPS8L3. Several potential MST3 substrates could contribute to the regulation of dendritic actin cytoskeleton: Arhgap18 is a Rho-GTPase activating protein (GAP) (Maeda et al., 2011), PAK6 is an effector of the Rho family GTPases, and Formin-like protein 2 (FMNL2) is an actin binding protein that can bind and bundle actin filaments (Schönichen and Geyer, 2010). In addition, we identified two oligodendrocyte-specific cytoskeleton regulators, namely TPPP/p25alpha (Song et al., 2007) and Ermin (an actin binding protein) (Brockschnieder et al., 2006), as putative MST3 substrates, further implicating MST3 in cytoskeletal regulation in oligodendrocytes.

There were two of the putative substrates, GIPC and Tsg101, that suggest a role for MST3 in the regulation of membrane trafficking. GAIP interacting protein, C terminus (GIPC) is a PDZ domain containing protein, which regulates trafficking of membrane proteins including *N*-methyl-D-aspartate (NMDA) receptors in neurons (Yi et al., 2007). Tumor susceptibility gene 101 (Tsg101) is a component of the endosomal sorting complex, required for ubiquitin-dependent sorting of proteins into endosomes.

Identification of Caspase 3 as a putative MST3 substrate is intriguing, as Caspase 3 is activated by MST3 in human trophoblasts (Wu et al., 2008, 2011). Whereas the MST3 phosphorylation site is not conserved in human Caspase 3, a potential phosphorylation site (T16) is found in a similar location in human Caspase 6-beta. An interesting hypothesis is that this activation might arise from direct phosphorylation of Caspase 3 or Caspase 6 by MST3 in mice and humans, respectively.

Cystine glutamate transporter/Slc7a11, a transporter associated with glutamate toxicity in neurons, a putative substrate of MST3, is active in both neurons and glia (Bridges et al., 2012; Dixon et al., 2012; Jackman et al., 2012). Finally, the identification of eukaryotic elongation factor 1a as a putative MST3 target has implications in translational regulation.

Spine morphogenesis is regulated by NDR1/2 (Ultanir et al., 2012). Notably, although both NDR1/2 and MST3 kinases alter spine morphogenesis, they do so via distinct sets of putative substrates identified by our chemical genetic screen. NDR1/2′s substrates implicate membrane trafficking regulation whereas MST3′s substrates implicate cytoskeleton regulation.

Roles of the Phosphorylation Targets of Thousand and One Amino Acid 1 and Thousand and One Amino Acid 2 Kinases in Dendritic Filopodia and Spine Development

Expression of phospho mutant and phospho-mimetic mutant substrates could act as dominant-negative and constitutively active forms of the effectors, thereby resulting in phenotypes similar to the loss or gain of function phenotypes of MST3. Among the tested substrates, we found that TAO1 and TAO2 phospho mutants resulted in phenotypes strikingly similar to that of MST3 loss of function. We do not rule out the involvement of any of the other putative substrates, as not all phospho mutants may act as dominant negatives. Our screen, however, was useful in identifying TAO1/2 kinases as likely downstream effectors for more in depth investigation. Using shRNA expression, we demonstrated that TAO1/2, and in particular the TAO2 activity, is required for dendritic filopodia development and maintenance of spines. We further confirmed that MST3 is necessary and sufficient to phosphorylate TAO1/2.

TAO1 and its homolog TAO2 are serine/threonine kinases in the Ste20 kinase family. TAO1 is also called microtubule affinity regulating kinase kinase, and it regulates microtubule affinity regulating kinase (MARK/Par1) via phosphorylation (Timm et al., 2003). MARK, in turn, phosphorylates MAPs to cause microtubule breakdown (Drewes et al., 1997). Implicated in behaviors induced by ethanol, nicotine, and cocaine, *Drosophila* TAO genetically interacts with the microtubule regulator Par1 and is required for the development of the mushroom body in the *Drosophila* brain (King et al., 2011). TAO1 colocalizes with microtubules in S2 cells and limits microtubule growth (Liu et al., 2010). TAO1 may also form a link between microtubules and actin cytoskeleton by interacting with actin and microtubule binding proteins (Johne et al., 2008; Mitsopoulos et al., 2003). It remains to be determined how TAO1 functions in cytoskeleton regulation and whether its functions depend on MST3 phosphorylation.

TAO2 is highly similar to TAO1 in its kinase domain. TAO1 and TAO2 are expressed in the brain (Hutchison et al., 1998) and are involved in p38 MAP kinase regulation (Chen et al., 1999; Hutchison et al., 1998). Interestingly, TAO2 has also been implicated as a candidate gene for the autism spectrum disorder (Weiss et al., 2008), a neurodevelopmental disorder. TAO2 is localized to actin

CellPress

cytoskeleton in dendritic growth cones in the first postnatal week and is involved in basal dendrite development in cortical neurons via its interaction with the semaphorin 3A receptor Neuropilin and subsequent c-Jun N-terminal kinase activation (de Anda et al., 2012). Arcadlin, an activity-regulated cell adhesion molecule, can bind N-cadherin and a shorter splice isoform of TAO2, TAO2b, to activate p38 MAP kinase, resulting in N-cadherin internalization (Yasuda et al., 2007). Actin and microtubule often interact in cytoskeleton. Of particular interest is the regulation of Myosin Va's association in dendritic cytoskeleton as described below.

Thousand and One Amino Acid 1/2 Binding and Regulation of Myosin V Motors

Phosphorylation is a posttranslational modification that may alter protein function by affecting its interaction with binding partners. The observation that the TAO1A and TAO2A phospho mutant expression increases filopodia density underscores the significance of MST3 phosphorylation. We hypothesize that these mutant kinases might be binding and titrating components of TAO1/2 signaling, thus acting as a dominant negative.

Using a SILAC based immunopreciptation method, we identified a protein complex including Myosin Va, Myosin light chain 6, and DrebrinE that bind peptides bearing the TAO1/2 phosphorylation site in a phosphorylation dependent manner. Unlike TAO1WT, TAO1A is unable to bind Myosin Va. We further confirmed the phosphospecific binding of Myosin Va to TAO1/2 via coimmunoprecipitation. Moreover, endogenous Myosin Va can bind endogenous TAO1 and phosphorylated TAO1/2 in neurons.

Myosin Va is an abundant protein in the brain and is present in synaptic fractions (Walikonis et al., 2000). An unconventional actin motor protein, Myosin Va in vertebrates can tether cargo to actin cytoskeleton at the cell periphery (Wu et al., 1998); reduce microtubule based transport when recruited on the same cargo with the microtubule motor kinesin (Kapitein et al., 2013); and perform short-range transport of cargo on actin filaments (Hammer and Sellers, 2012; Kapitein et al., 2013). In hippocampal neurons, both Myosin Va (Correia et al., 2008) and Myosin Vb (Lisé et al., 2006; Wang et al., 2008) are implicated in trafficking of AMPA type glutamate receptors into dendritic spines, although previous studies showed no defects in basal or plasticity induced AMPA receptor recruitment to synapses in Myosin Va mutant mice Dilute-Lethal (Petralia et al., 2001; Schnell and Nicoll, 2001). Myosin Va is also implicated in transporting endoplasmic reticulum in Purkinje neuron dendritic spines (Wagner et al., 2011). Myosin Va knockdown in neuronal cultures drastically reduces spine density (Lisé et al., 2009). A recent examination of the Myosin Va neurological mutant mice flailer, which express a dominant-negative Myosin Va fusion protein lacking the actin binding domain, revealed a reduction of mature dendritic spines and an increase in dendritic filopodia (Yoshii et al., 2013). In these mutant mice, the mature excitatory synaptic markers PSD-95, stargazin, the endocytic zone marker Dynamin 3, and AMPA receptors are drastically mislocalized to dendritic shaft (Yoshii et al., 2013). These data support the notion that Myosin Va is critical for mature spine synapse formation. Interestingly, despite the loss of mature spine synapses in flailer mice, the AMPA receptor currents are increased (Yoshii et al., 2013). Our observation of increased Myosin Vb trafficking to dendrites in the absence of TAO1/2 and dendritic Myosin Va is indicative of a compensatory Myosin Vb function in the absence of Myosin Va.

We find that Myosin Va-GTD is localized in a punctate pattern along the dendrites and colocalizes with the recycling endosome marker Rab11, and we show that this recruitment is dependent on TAO1/2 function. Membrane transport from recycling endosomes is required for maintenance of dendritic spine structure as well as for delivery of AMPA receptors during synaptic plasticity (Park et al., 2006). TAO1 colocalizes with Myosin Va-GTD independent of its T440 phosphorylation, implicating a mechanism for recruitment of TAO1 into Myosin Va complexes that is distinct from their interaction via the T440 region. Interestingly, TAO1A shifts Myosin Va distribution from dendrites to neuronal cell body, suggesting that the T440 site on TAO1 is important in regulating TAO1 function and dendritic localization of Myosin Va in controlling spine development.

The opposite effects of TAO1/2 shRNA knockdown on the expression of Myosin Va and Myosin Vb could reflect a homeostatic response to the reduction of Myosin Va. How TAO1/2 regulates Myosin Va remains an open question. A possibility is that TAO1/2 regulates Myosin V motor function by binding and phosphorylating components of the Myosin Va complex, and enhancing its dendritic localization.

Chemical Genetics for Kinase Substrate Identification

A chemical genetic screen for kinase substrate identification is an unbiased method to identify substrates in relatively intact protein lysates without the need for peptide arrays or gel electrophoresis. The combination of simple chemistry to highly enrich for peptides of substrates phosphorylated by a kinase of interest, and the ability to identify the substrates as well as the phosphorylation sites by using powerful liquid chromatography/tandem mass spectrometry renders this technique uniquely advantageous for studying downstream effectors of kinases.

Phosphorylation often alters protein function by changing binding properties of substrates. We have now added a further technical advance to the kinase substrate identification screen through which we can determine the phosphorylation state dependent protein interactors that bind the substrates on the phosphorylation sites. Using a quantitative, unbiased peptide pull-down screen approach, which can be achieved by SILAC labeling of proteins in neuronal cultures, we were able to identify protein interactors of TAO1/2 that critically depend on their MST3 phosphorylation site. We believe our methodology of chemical genetic kinase substrate identification, followed by determining the functionality of the phosphorylation is a useful paradigm in delineating kinase signaling.

EXPERIMENTAL PROCEDURES

Neuronal Cultures, DNA Constructs, Small Hairpin RNA, and Lentiviruses

Hippocampal neurons were cultured from E19 Long-Evans rats (Charles River Lab) in accordance with local guidelines at the University of California San Francisco (UCSF) and National Institute for Medical Research as described before (Ultanir et al., 2012). Mouse MST3 (BC004650) cDNA purchased from ATCC was cloned in pRK5 mammalian expression vector with N-terminal

HA tag. Human full-length TAO2 (1,235 amino acids) in pCMV Sport 6.0 expression vector was a gift from Dr. Froylan Calderon De Anda and Dr. Li-Huei Tsai from the Massachusetts Institute of Technology (de Anda et al., 2012). Human FMNL2, human ArhGAP18, mouse GIPC, mouse EPS8, human TAO1, and human PAK6 were purchased from Thermo Scientific and cloned into pRK5 vector with N-terminal HA tag. Mutations were generated by site directed mutagenesis. Myosin Va-GTD-GFP, Myosin Va-GTD-HA, and Myosin Vb-GTD-HA vectors were gifts from Dr. Don Arnold from the University of South California (Lewis et al., 2009).

All shRNA sequences were 19 base pairs long and were selected via http://katahdin.cshl.org/html/scripts/main.pl. Hairpins targeting these sequences were cloned in pLentiLox 3.7, which expresses EGFP via a separate promoter in addition to expressing shRNA via a U6 promoter. Lentiviruses were generated at UCSF core facilities. All clones were verified by sequencing.

In Utero Electroporation

In utero electroporation surgery was performed in accordance with local guidelines as described (Ultanir et al., 2012). Embryos were injected with 1 μg/μl pCAG-GFP (control), or 0.4 μg/μl pCAG-GFP + 1 μg/μl MST3 shRNA #1, or 0.4 μg/μl pCAG-GFP + 1 μg/μl TAO1/2 shRNA. Mice were perfused at P18–P20 using 4% paraformaldehyde and 4% sucrose.

Antibodies and Confocal Microscopy

For a detailed list of antibodies and dilutions, please see Supplemental Experimental Procedures. Dendrites were imaged using an inverted Leica SP5 confocal microscope using a 63× (NA 1.4) objective at 6× zoom. Z sections were obtained across the dendrite depth at 0.5 μm z intervals. Spine density and categorization was done manually using Leica image analysis. Spine head diameter measurements were done using a custom plugin in ImageJ (Ultanir et al., 2007).

Mammalian Sterile 20-like Kinase 3 Kinase Assays

HA tagged MST3 was expressed in COS7 or HEK293T cells for 48 hr before purified via HA epitope tag using Anti-HA Affinity matrix (Roche, clone 3F10). Kinase assays were conducted in 20 mM Tris-HCl pH 7.5, 10 mM MgCl2, 1 mM dithiothreitol (DTT), 100 μM ATP, 1× Phosphatase inhibitor cocktail, and 0.5 mM of an ATP analog for 30 min at 30°C. Reaction was followed by alkylation for 1 hr at room temperature by addition of 2 μl 100 mM PNBM per 30 μl of kinase reaction.

Mammalian Sterile 20-like Kinase 3 Substrate Labeling and Identification

Kinase substrate labeling was done in P3 and P13 mouse brain lysates obtained as described (Ultanir et al., 2012). Purified MST3-MG and MST3-KD were used for labeling. Covalent capture method for kinase substrate identification and mass spectrometry was done as previously described (Ultanir et al., 2012).

Multiplex Stable Isotope Labeling by Amino Acids in Culture Labeling of Neuronal Cultures and Phospho-Peptide Pull-Down

Cortical neuronal cultures were labeled using Multiplex SILAC (Zhang et al., 2011), and peptide pull-downs using SILAC labeled lysates was conducted as described (Stephanowitz et al., 2012). TAO2 T475 peptide (C RNRDHFAT*IRTASLVSR) was synthesized with or without a phosphorylation at the indicated threonine and conjugated to iodoacetyl agarose. Precipitated proteins were subjected to in-bead typsin digest followed by reversed-phase liquid chromatography-electrospray tandem mass spectrometry (LC-MS/MS) analysis.

Immunoprecipitations

HEK293T cells transfected with Myosin Va-GTD-GFP, control GFP construct, HA-TAO1, and HA-TAO1A, separately. Lysate from Myosin Va-GFP or control GFP transfected cells was incubated with Protein G beads prebound with monoclonal GFP antibody, and then lysates from either HA-TAO1 or HA-TAO1A transfected cells were added and incubated with the beads containing Myosin V-GFP or GFP alone. For immunoprecipitation from neurons precleared lysates from DIV6 or DIV9 rat hippocampal neurons were incubated with Myosin Va antibody bound Protein A beads (Sigma) overnight at 4°C.

SUPPLEMENTAL INFORMATION

Supplemental Information includes Supplemental Experimental Procedures, six figures, and one table and can be found with this article online at http://dx.doi.org/10.1016/j.neuron.2014.10.025.

AUTHOR CONTRIBUTIONS

S.K.U. designed and performed spine and filopodia phenotype analysis in cultures and in vivo, MST3 substrate labeling, SILAC phosphopeptide pull-down, and wrote the manuscript with help from other authors. S.Y. designed and performed the immunoprecipitations, phosphoTAO1/2 staining, and contributed to immunostainings and biochemistry experiments on shRNA knockdown in neurons and HEK293 cells. N.T.H. designed and performed the covalent capture and MS for kinase substrate identification. J.A.O.-P. codesigned and performed MS for SILAC samples. S.C. contributed to neuronal cultures and cloned several constructs. A.L.B., K.M.S., L.Y.J., and Y.N.J. oversaw the project development.

ACKNOWLEDGMENTS

We thank Lucas Baltussen, Rafael Krumkamp, Patricia Garcez, and Koji Oishi for technical help. We thank Jan and Shokat Laboratory members for discussion and critical reading of the manuscript. MS analysis was provided by the Bio-Organic Biomedical Mass Spectrometry Resource at UCSF (A.L. Burlingame, Director), supported by funding from the Biomedical Technology Research Centers program of the NIH National Institute of General Medical Sciences, NIH NIGMS 8P41GM103481, and Howard Hughes Medical Institute. This work was supported by the National Alliance for Research on Schizophrenia and Depression (NARSAD) Young Investigator Award to S.K.U., NARSAD Distinguished Investigator Award to Y.N.J., NIH grants R37NS040929 and 5R01MH084234 to Y.N.J., NIH grant RO1EB001987 to K.M.S., and Genentech predoctoral fellowship to N.T.H. K.M.S., L.Y.J., and Y.N.J. are investigators of Howard Hughes Medical Institute.

Accepted: September 30, 2014
Published: November 13, 2014

REFERENCES

Allen, J.J., Li, M., Brinkworth, C.S., Paulson, J.L., Wang, D., Hübner, A., Chou, W.H., Davis, R.J., Burlingame, A.L., Messing, R.O., et al. (2007). A semisynthetic epitope for kinase substrates. Nat. Methods *4*, 511–516.

Blethrow, J., Zhang, C., Shokat, K.M., and Weiss, E.L. (2004). Design and use of analog-sensitive protein kinases. In Current Protocols in Molecular Biology, F.M. Ausubel, ed., http://dx.doi.org/10.1002/0471142727.mb1811s66, Chapter 18, Unit 18 11.

Blethrow, J.D., Glavy, J.S., Morgan, D.O., and Shokat, K.M. (2008). Covalent capture of kinase-specific phosphopeptides reveals Cdk1-cyclin B substrates. Proc. Natl. Acad. Sci. USA *105*, 1442–1447.

Bridges, R., Lutgen, V., Lobner, D., and Baker, D.A. (2012). Thinking outside the cleft to understand synaptic activity: contribution of the cystine-glutamate antiporter (System xc-) to normal and pathological glutamatergic signaling. Pharmacol. Rev. *64*, 780–802.

Brockschnieder, D., Sabanay, H., Riethmacher, D., and Peles, E. (2006). Ermin, a myelinating oligodendrocyte-specific protein that regulates cell morphology. J. Neurosci. *26*, 757–762.

Chen, Z., Hutchison, M., and Cobb, M.H. (1999). Isolation of the protein kinase TAO2 and identification of its mitogen-activated protein kinase/extracellular signal-regulated kinase kinase binding domain. J. Biol. Chem. *274*, 28803–28807.

Correia, S.S., Bassani, S., Brown, T.C., Lisé, M.F., Backos, D.S., El-Husseini, A., Passafaro, M., and Esteban, J.A. (2008). Motor protein-dependent transport of AMPA receptors into spines during long-term potentiation. Nat. Neurosci. *11*, 457–466.

D'Amelio, M., Sheng, M., and Cecconi, F. (2012). Caspase-3 in the central nervous system: beyond apoptosis. Trends Neurosci. *35*, 700–709.

de Anda, F.C., Rosario, A.L., Durak, O., Tran, T., Gräff, J., Meletis, K., Rei, D., Soda, T., Madabhushi, R., Ginty, D.D., et al. (2012). Autism spectrum disorder susceptibility gene TAOK2 affects basal dendrite formation in the neocortex. Nat. Neurosci. *15*, 1022–1031.

Dixon, S.J., Lemberg, K.M., Lamprecht, M.R., Skouta, R., Zaitsev, E.M., Gleason, C.E., Patel, D.N., Bauer, A.J., Cantley, A.M., Yang, W.S., et al. (2012). Ferroptosis: an iron-dependent form of nonapoptotic cell death. Cell *149*, 1060–1072.

Drewes, G., Ebneth, A., Preuss, U., Mandelkow, E.M., and Mandelkow, E. (1997). MARK, a novel family of protein kinases that phosphorylate microtubule-associated proteins and trigger microtubule disruption. Cell *89*, 297–308.

Emoto, K., He, Y., Ye, B., Grueber, W.B., Adler, P.N., Jan, L.Y., and Jan, Y.N. (2004). Control of dendritic branching and tiling by the Tricornered-kinase/Furry signaling pathway in Drosophila sensory neurons. Cell *119*, 245–256.

Emoto, K., Parrish, J.Z., Jan, L.Y., and Jan, Y.N. (2006). The tumour suppressor Hippo acts with the NDR kinases in dendritic tiling and maintenance. Nature *443*, 210–213.

Fiala, J.C., Feinberg, M., Popov, V., and Harris, K.M. (1998). Synaptogenesis via dendritic filopodia in developing hippocampal area CA1. J. Neurosci. *18*, 8900–8911.

Fuller, S.J., McGuffin, L.J., Marshall, A.K., Giraldo, A., Pikkarainen, S., Clerk, A., and Sugden, P.H. (2012). A novel non-canonical mechanism of regulation of MST3 (mammalian Sterile20-related kinase 3). Biochem. J. *442*, 595–610.

Gallegos, M.E., and Bargmann, C.I. (2004). Mechanosensory neurite termination and tiling depend on SAX-2 and the SAX-1 kinase. Neuron *44*, 239–249.

Hammer, J.A., 3rd, and Sellers, J.R. (2012). Walking to work: roles for class V myosins as cargo transporters. Nat. Rev. Mol. Cell Biol. *13*, 13–26.

Harris, K.M., and Kater, S.B. (1994). Dendritic spines: cellular specializations imparting both stability and flexibility to synaptic function. Annu. Rev. Neurosci. *17*, 341–371.

Hengeveld, R.C., Hertz, N.T., Vromans, M.J., Zhang, C., Burlingame, A.L., Shokat, K.M., and Lens, S.M. (2012). Development of a chemical genetic approach for human aurora B kinase identifies novel substrates of the chromosomal passenger complex. Mol. Cell. Proteomics *11*, 47–59.

Hertz, N.T., Wang, B.T., Allen, J.J., Zhang, C., Dar, A.C., Burlingame, A.L., and Shokat, K.M. (2010). Chemical genetic approach for kinase-substrate mapping by covalent capture of thiophosphopeptides and analysis by mass spectrometry. Curr. Protoc. Chem. Biol. *2*, 15–36.

Huang, C.Y., Wu, Y.M., Hsu, C.Y., Lee, W.S., Lai, M.D., Lu, T.J., Huang, C.L., Leu, T.H., Shih, H.M., Fang, H.I., et al. (2002). Caspase activation of mammalian sterile 20-like kinase 3 (Mst3). Nuclear translocation and induction of apoptosis. J. Biol. Chem. *277*, 34367–34374.

Huguet, G., Ey, E., and Bourgeron, T. (2013). The genetic landscapes of autism spectrum disorders. Annu. Rev. Genomics Hum. Genet. *14*, 191–213.

Hutchison, M., Berman, K.S., and Cobb, M.H. (1998). Isolation of TAO1, a protein kinase that activates MEKs in stress-activated protein kinase cascades. J. Biol. Chem. *273*, 28625–28632.

Irwin, N., Li, Y.M., O'Toole, J.E., and Benowitz, L.I. (2006). Mst3b, a purine-sensitive Ste20-like protein kinase, regulates axon outgrowth. Proc. Natl. Acad. Sci. USA *103*, 18320–18325.

Jackman, N.A., Melchior, S.E., Hewett, J.A., and Hewett, S.J. (2012). Non-cell autonomous influence of the astrocyte system xc- on hypoglycaemic neuronal cell death. ASN Neuro. *4*, 4.

Jan, Y.N., and Jan, L.Y. (2010). Branching out: mechanisms of dendritic arborization. Nat. Rev. Neuro. *11*, 316–328.

Johne, C., Matenia, D., Li, X.Y., Timm, T., Balusamy, K., and Mandelkow, E.M. (2008). Spred1 and TESK1—two new interaction partners of the kinase MARKK/TAO1 that link the microtubule and actin cytoskeleton. Mol. Biol. Cell *19*, 1391–1403.

Kapitein, L.C., van Bergeijk, P., Lipka, J., Keijzer, N., Wulf, P.S., Katrukha, E.A., Akhmanova, A., and Hoogenraad, C.C. (2013). Myosin-V opposes microtubule-based cargo transport and drives directional motility on cortical actin. Curr. Biol. *23*, 828–834.

King, I., Tsai, L.T., Pflanz, R., Voigt, A., Lee, S., Jäckle, H., Lu, B., and Heberlein, U. (2011). Drosophila tao controls mushroom body development and ethanol-stimulated behavior through par-1. J. Neurosci. *31*, 1139–1148.

Lewis, T.L., Jr., Mao, T., Svoboda, K., and Arnold, D.B. (2009). Myosin-dependent targeting of transmembrane proteins to neuronal dendrites. Nat. Neurosci. *12*, 568–576.

Lisé, M.F., Wong, T.P., Trinh, A., Hines, R.M., Liu, L., Kang, R., Hines, D.J., Lu, J., Goldenring, J.R., Wang, Y.T., and El-Husseini, A. (2006). Involvement of myosin Vb in glutamate receptor trafficking. J. Biol. Chem. *281*, 3669–3678.

Lisé, M.F., Srivastava, D.P., Arstikaitis, P., Lett, R.L., Sheta, R., Viswanathan, V., Penzes, P., O'Connor, T.P., and El-Husseini, A. (2009). Myosin-Va-interacting protein, RILPL2, controls cell shape and neuronal morphogenesis via Rac signaling. J. Cell Sci. *122*, 3810–3821.

Liu, T., Rohn, J.L., Picone, R., Kunda, P., and Baum, B. (2010). Tao-1 is a negative regulator of microtubule plus-end growth. J. Cell Sci. *123*, 2708–2716.

Lorber, B., Howe, M.L., Benowitz, L.I., and Irwin, N. (2009). Mst3b, an Ste20-like kinase, regulates axon regeneration in mature CNS and PNS pathways. Nat. Neurosci. *12*, 1407–1414.

Maeda, M., Hasegawa, H., Hyodo, T., Ito, S., Asano, E., Yuang, H., Funasaka, K., Shimokata, K., Hasegawa, Y., Hamaguchi, M., and Senga, T. (2011). ARHGAP18, a GTPase-activating protein for RhoA, controls cell shape, spreading, and motility. Mol. Biol. Cell *22*, 3840–3852.

Menna, E., Disanza, A., Cagnoli, C., Schenk, U., Gelsomino, G., Frittoli, E., Hertzog, M., Offenhauser, N., Sawallisch, C., Kreienkamp, H.J., et al. (2009). Eps8 regulates axonal filopodia in hippocampal neurons in response to brain-derived neurotrophic factor (BDNF). PLoS Biol. *7*, e1000138.

Menna, E., Zambetti, S., Morini, R., Donzelli, A., Disanza, A., Calvigioni, D., Braida, D., Nicolini, C., Orlando, M., Fossati, G., et al. (2013). Eps8 controls dendritic spine density and synaptic plasticity through its actin-capping activity. EMBO J. *32*, 1730–1744.

Mitsopoulos, C., Zihni, C., Garg, R., Ridley, A.J., and Morris, J.D. (2003). The prostate-derived sterile 20-like kinase (PSK) regulates microtubule organization and stability. J. Biol. Chem. *278*, 18085–18091.

Nimchinsky, E.A., Sabatini, B.L., and Svoboda, K. (2002). Structure and function of dendritic spines. Annu. Rev. Physiol. *64*, 313–353.

Park, M., Salgado, J.M., Ostroff, L., Helton, T.D., Robinson, C.G., Harris, K.M., and Ehlers, M.D. (2006). Plasticity-induced growth of dendritic spines by exocytic trafficking from recycling endosomes. Neuron *52*, 817–830.

Parrish, J.Z., Kim, M.D., Jan, L.Y., and Jan, Y.N. (2006). Genome-wide analyses identify transcription factors required for proper morphogenesis of Drosophila sensory neuron dendrites. Genes Dev. *20*, 820–835.

Petralia, R.S., Wang, Y.X., Sans, N., Worley, P.F., Hammer, J.A., 3rd, and Wenthold, R.J. (2001). Glutamate receptor targeting in the postsynaptic spine involves mechanisms that are independent of myosin Va. Eur. J. Neurosci. *13*, 1722–1732.

Preisinger, C., Short, B., De Corte, V., Bruyneel, E., Haas, A., Kopajtich, R., Gettemans, J., and Barr, F.A. (2004). YSK1 is activated by the Golgi matrix protein GM130 and plays a role in cell migration through its substrate 14-3-3zeta. J. Cell Biol. *164*, 1009–1020.

Schinkmann, K., and Blenis, J. (1997). Cloning and characterization of a human STE20-like protein kinase with unusual cofactor requirements. J. Biol. Chem. *272*, 28695–28703.

Schnell, E., and Nicoll, R.A. (2001). Hippocampal synaptic transmission and plasticity are preserved in myosin Va mutant mice. J. Neurophysiol. *85*, 1498–1501.

Schönichen, A., and Geyer, M. (2010). Fifteen formins for an actin filament: a molecular view on the regulation of human formins. Biochim. Biophys. Acta *1803*, 152–163.

Song, Y.J., Lundvig, D.M., Huang, Y., Gai, W.P., Blumbergs, P.C., Højrup, P., Otzen, D., Halliday, G.M., and Jensen, P.H. (2007). p25alpha relocalizes in oligodendroglia from myelin to cytoplasmic inclusions in multiple system atrophy. Am. J. Pathol. *171*, 1291–1303.

Spellman, D.S., Deinhardt, K., Darie, C.C., Chao, M.V., and Neubert, T.A. (2008). Stable isotopic labeling by amino acids in cultured primary neurons: application to brain-derived neurotrophic factor-dependent phosphotyrosine-associated signaling. Mol. Cell. Proteomics *7*, 1067–1076.

Stamatakou, E., Marzo, A., Gibb, A., and Salinas, P.C. (2013). Activity-dependent spine morphogenesis: a role for the actin-capping protein Eps8. J. Neurosci. *33*, 2661–2670.

Stegert, M.R., Hergovich, A., Tamaskovic, R., Bichsel, S.J., and Hemmings, B.A. (2005). Regulation of NDR protein kinase by hydrophobic motif phosphorylation mediated by the mammalian Ste20-like kinase MST3. Mol. Cell. Biol. *25*, 11019–11029.

Stephanowitz, H., Lange, S., Lang, D., Freund, C., and Krause, E. (2012). Improved two-dimensional reversed phase-reversed phase LC-MS/MS approach for identification of peptide-protein interactions. J. Proteome Res. *11*, 1175–1183.

Tang, J., Ip, J.P., Ye, T., Ng, Y.P., Yung, W.H., Wu, Z., Fang, W., Fu, A.K., and Ip, N.Y. (2014). Cdk5-dependent Mst3 phosphorylation and activity regulate neuronal migration through RhoA inhibition. J. Neurosci. *34*, 7425–7436.

Timm, T., Li, X.Y., Biernat, J., Jiao, J., Mandelkow, E., Vandekerckhove, J., and Mandelkow, E.M. (2003). MARKK, a Ste20-like kinase, activates the polarity-inducing kinase MARK/PAR-1. EMBO J. *22*, 5090–5101.

Ultanir, S.K., Kim, J.E., Hall, B.J., Deerinck, T., Ellisman, M., and Ghosh, A. (2007). Regulation of spine morphology and spine density by NMDA receptor signaling in vivo. Proc. Natl. Acad. Sci. USA *104*, 19553–19558.

Ultanir, S.K., Hertz, N.T., Li, G., Ge, W.P., Burlingame, A.L., Pleasure, S.J., Shokat, K.M., Jan, L.Y., and Jan, Y.N. (2012). Chemical genetic identification of NDR1/2 kinase substrates AAK1 and Rabin8 Uncovers their roles in dendrite arborization and spine development. Neuron *73*, 1127–1142.

Wagner, W., Brenowitz, S.D., and Hammer, J.A., 3rd. (2011). Myosin-Va transports the endoplasmic reticulum into the dendritic spines of Purkinje neurons. Nat. Cell Biol. *13*, 40–48.

Walikonis, R.S., Jensen, O.N., Mann, M., Provance, D.W., Jr., Mercer, J.A., and Kennedy, M.B. (2000). Identification of proteins in the postsynaptic density fraction by mass spectrometry. J. Neurosci. *20*, 4069–4080.

Wang, Z., Edwards, J.G., Riley, N., Provance, D.W., Jr., Karcher, R., Li, X.D., Davison, I.G., Ikebe, M., Mercer, J.A., Kauer, J.A., and Ehlers, M.D. (2008). Myosin Vb mobilizes recycling endosomes and AMPA receptors for postsynaptic plasticity. Cell *135*, 535–548.

Weiss, L.A., Shen, Y., Korn, J.M., Arking, D.E., Miller, D.T., Fossdal, R., Saemundsen, E., Stefansson, H., Ferreira, M.A., Green, T., et al.; Autism Consortium (2008). Association between microdeletion and microduplication at 16p11.2 and autism. N. Engl. J. Med. *358*, 667–675.

Wu, X., Bowers, B., Rao, K., Wei, Q., and Hammer, J.A., 3rd. (1998). Visualization of melanosome dynamics within wild-type and dilute melanocytes suggests a paradigm for myosin V function In vivo. J. Cell Biol. *143*, 1899–1918.

Wu, H.Y., Lin, C.Y., Lin, T.Y., Chen, T.C., and Yuan, C.J. (2008). Mammalian Ste20-like protein kinase 3 mediates trophoblast apoptosis in spontaneous delivery. Apoptosis *13*, 283–294.

Wu, H.Y., Lin, C.Y., Chen, T.C., Pan, S.T., and Yuan, C.J. (2011). Mammalian Ste20-like protein kinase 3 plays a role in hypoxia-induced apoptosis of trophoblast cell line 3A-sub-E. Int. J. Biochem. Cell Biol. *43*, 742–750.

Yasuda, S., Tanaka, H., Sugiura, H., Okamura, K., Sakaguchi, T., Tran, U., Takemiya, T., Mizoguchi, A., Yagita, Y., Sakurai, T., et al. (2007). Activity-induced protocadherin arcadlin regulates dendritic spine number by triggering N-cadherin endocytosis via TAO2beta and p38 MAP kinases. Neuron *56*, 456–471.

Yi, Z., Petralia, R.S., Fu, Z., Swanwick, C.C., Wang, Y.X., Prybylowski, K., Sans, N., Vicini, S., and Wenthold, R.J. (2007). The role of the PDZ protein GIPC in regulating NMDA receptor trafficking. J. Neurosci. *27*, 11663–11675.

Yoshii, A., Zhao, J.P., Pandian, S., van Zundert, B., and Constantine-Paton, M. (2013). A Myosin Va mutant mouse with disruptions in glutamate synaptic development and mature plasticity in visual cortex. J. Neurosci. *33*, 8472–8482.

Yuste, R., and Bonhoeffer, T. (2004). Genesis of dendritic spines: insights from ultrastructural and imaging studies. Nat. Rev. Neurosci *5*, 24–34.

Zallen, J.A., Peckol, E.L., Tobin, D.M., and Bargmann, C.I. (2000). Neuronal cell shape and neurite initiation are regulated by the Ndr kinase SAX-1, a member of the Orb6/COT-1/warts serine/threonine kinase family. Mol. Biol. Cell *11*, 3177–3190.

Zhang, G., Deinhardt, K., Chao, M.V., and Neubert, T.A. (2011). Study of neurotrophin-3 signaling in primary cultured neurons using multiplex stable isotope labeling with amino acids in cell culture. J. Proteome Res. *10*, 2546–2554.

Neuron, Volume *84*

Supplemental Information

MST3 Kinase Phosphorylates TAO1/2 to Enable Myosin Va Function in Promoting Spine Synapse Development

Sila K. Ultanir, Smita Yadav, Nicholas T. Hertz, Juan A. Oses-Prieto, Suzanne Claxton, Alma L. Burlingame, Kevan M. Shokat, Lily Y. Jan, and Yuh-Nung Jan

Inventory of Supplemental information

1. Supplemental Figure 1
2. Supplemental Figure 2
3. Supplemental Figure 3
4. Supplemental Figure 4
5. Supplemental Figure 5
6. Supplemental Figure 6
7. Supplemental Figure Legends
8. Supplemental Table 1 for Figure 6
9. Supplemental Experimental Procedures
10. Supplemental References

Figure S1

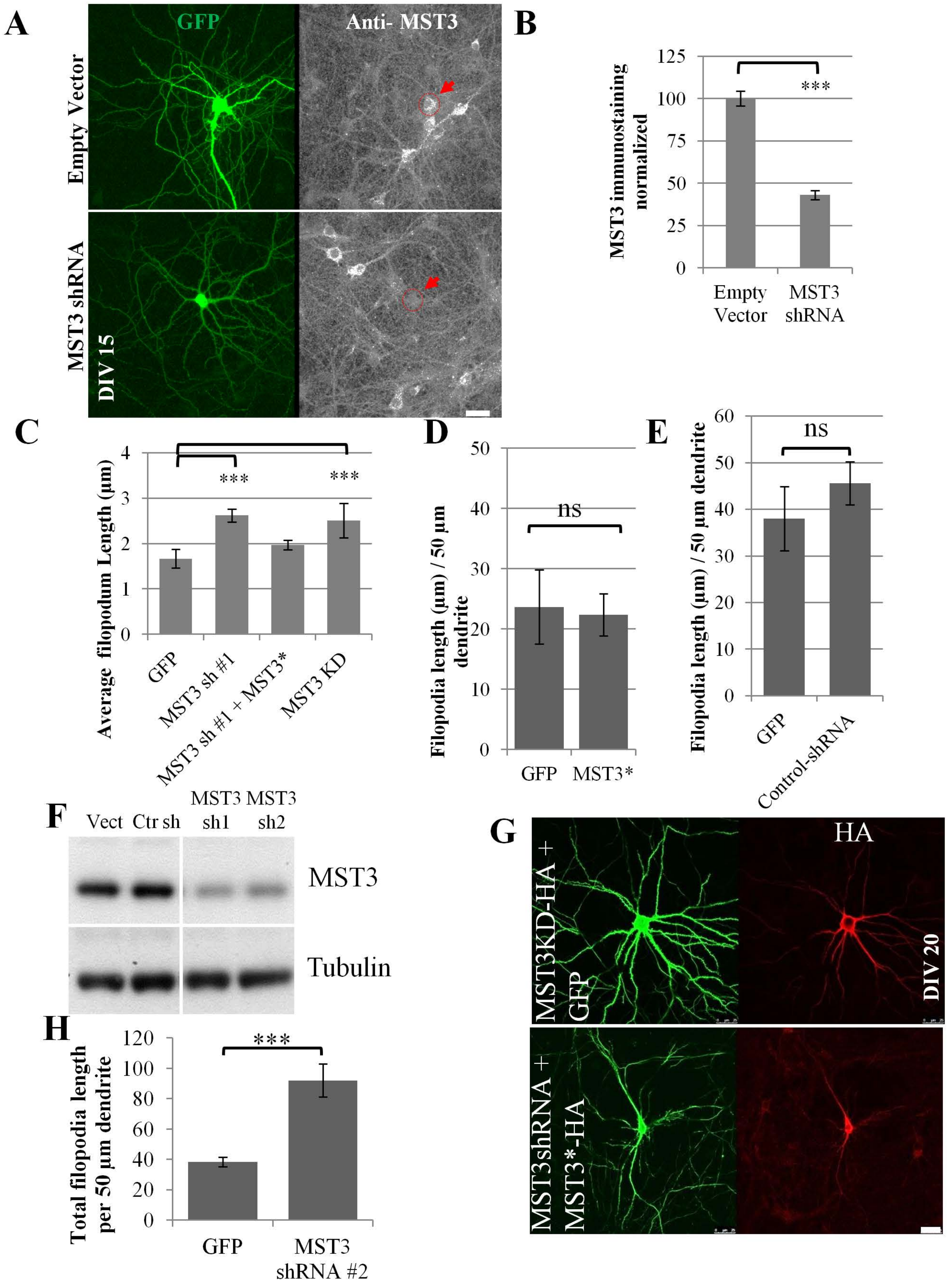
A
GFP
Anti- MST3
Empty Vector
MST3 shRNA
DIV 15
B
MST3 immunostaining normalized
125
100
75
50
25
0
***
Empty Vector
MST3 shRNA
C
Average filopodum Length (µm)
4
3
2
1
0
***
***
GFP
MST3 sh #1
MST3 sh #1 + MST3*
MST3 KD
D
Filopodia length (µm) / 50 µm dendrite
50
40
30
20
10
0
ns
GFP
MST3*
E
Filopodia length (µm) / 50 µm dendrite
60
50
40
30
20
10
0
ns
GFP
Control-shRNA
F
Vect
Ctr sh
MST3 sh1
MST3 sh2
MST3
Tubulin
G
HA
MST3KD-HA + GFP
DIV 20
MST3shRNA + MST3*-HA
H
Total filopodia length per 50 µm dendrite
120
100
80
60
40
20
0
***
GFP
MST3 shRNA #2

Figure S2

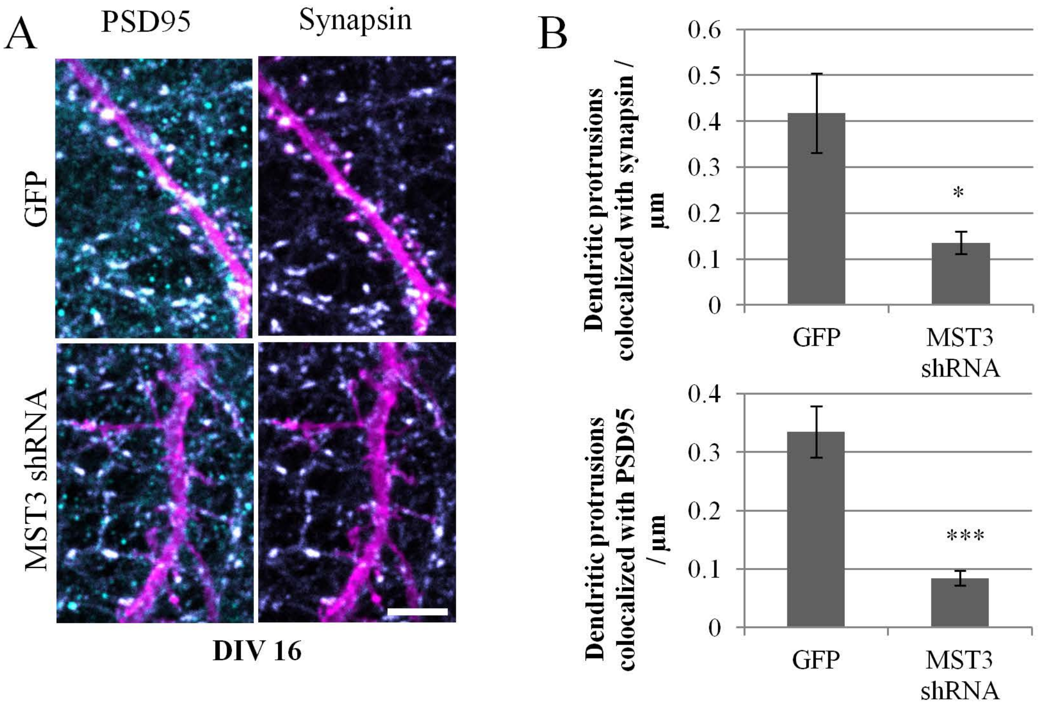
A
PSD95
Synapsin
GFP
MST3 shRNA
DIV 16
B
0.6
0.5
0.4
0.3
0.2
0.1
0
Dendritic protrusions colocalized with synapsin / µm
*
GFP
MST3 shRNA
0.4
0.3
0.2
0.1
0
Dendritic protrusions colocalized with PSD95 / µm
***
GFP
MST3 shRNA

Figure S3

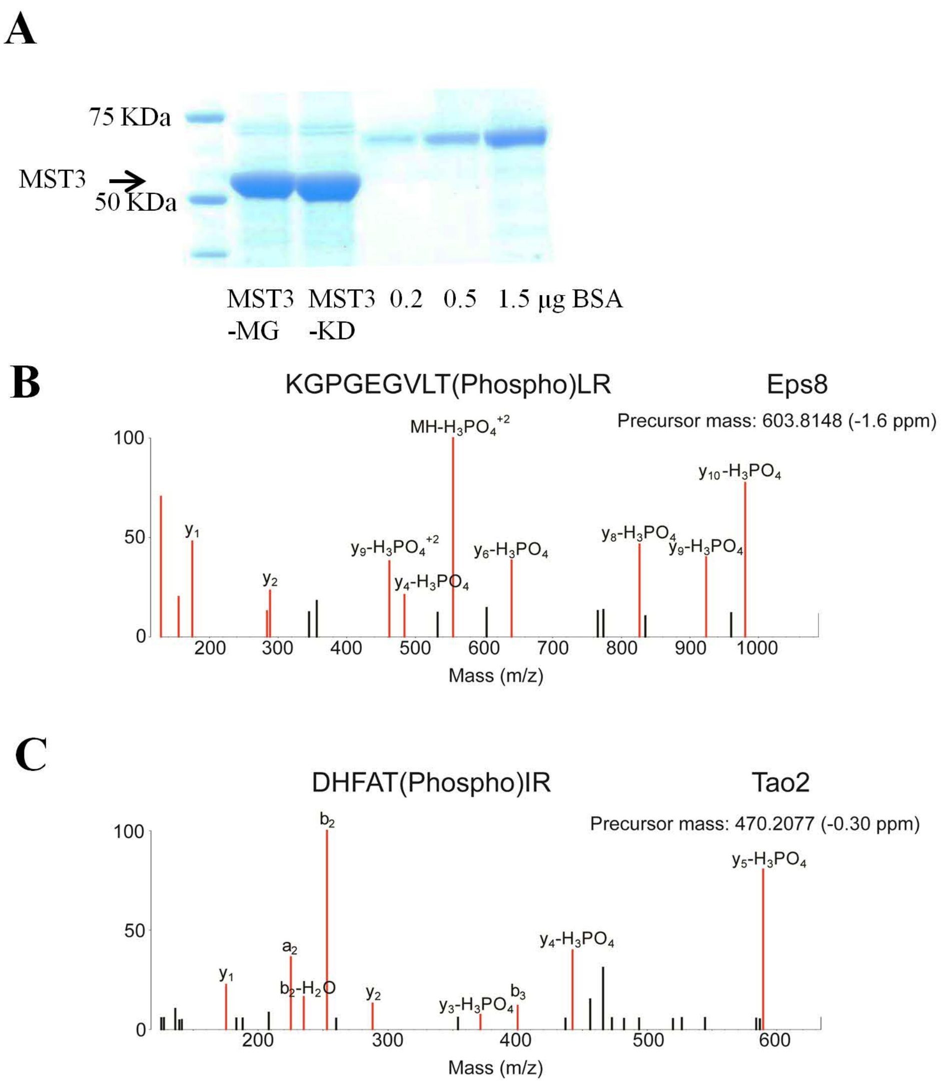
A
75 KDa
MST3
50 KDa
MST3 -MG
MST3 -KD
0.2
0.5
1.5 μg BSA
B
KGPGEGVLT(Phospho)LR
Eps8
Precursor mass: 603.8148 (-1.6 ppm)
Mass (m/z)
C
DHFAT(Phospho)IR
Tao2
Precursor mass: 470.2077 (-0.30 ppm)
Mass (m/z)

Figure S4

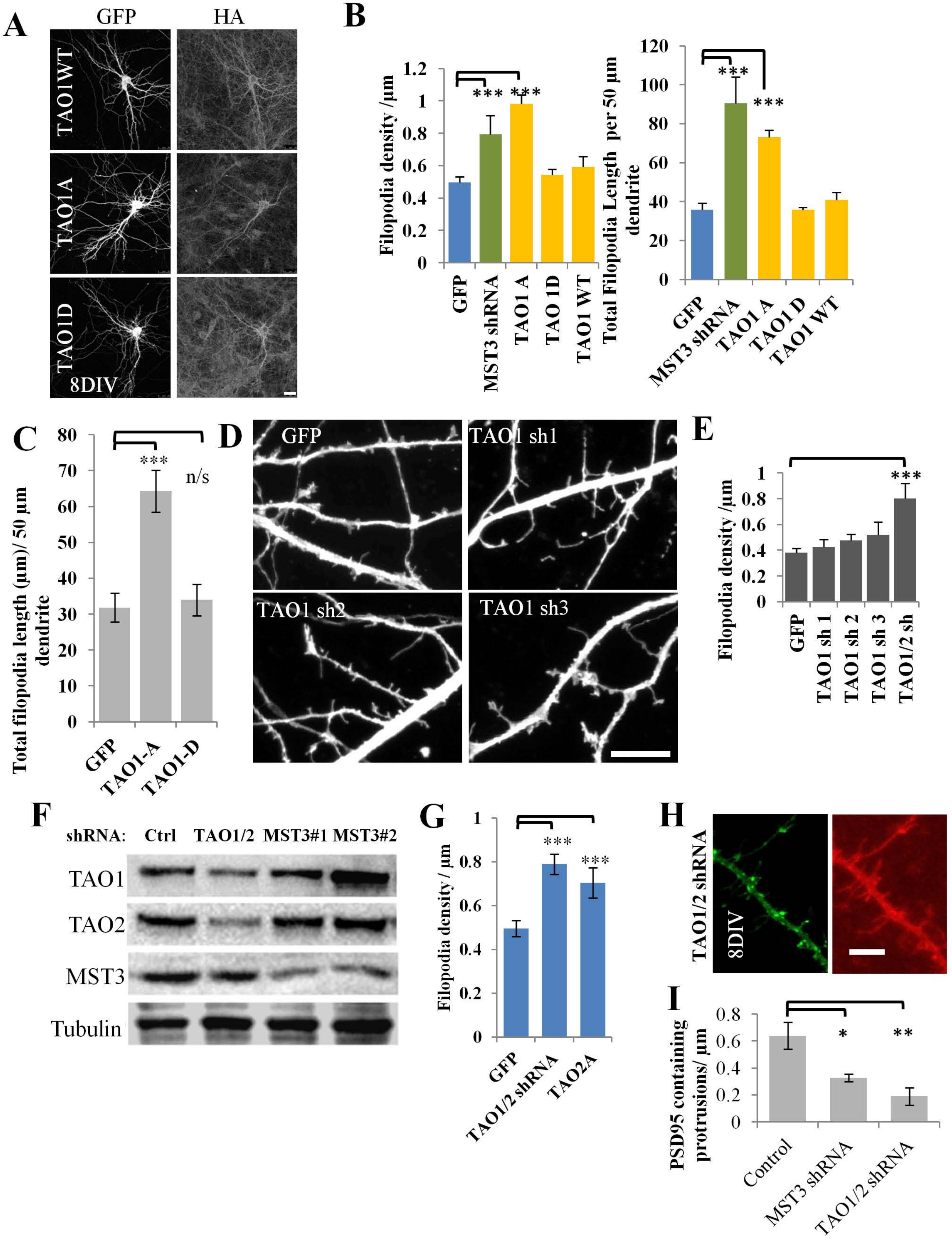

A
GFP
HA
TAO1WT
TAO1A
TAO1D
8DIV
B
Filopodia density /µm
1.2
1
0.8
0.6
0.4
0.2
0
***
***
GFP
MST3 shRNA
TAO1 A
TAO 1D
TAO1 WT
Total Filopodia Length per 50 µm dendrite
120
100
80
60
40
20
0
***
***
GFP
MST3 shRNA
TAO1 A
TAO1 D
TAO1 WT
C
Total filopodia length (µm)/ 50 µm dendrite
80
70
60
50
40
30
20
10
0
***
n/s
GFP
TAO1-A
TAO1-D
D
GFP
TAO1 sh1
TAO1 sh2
TAO1 sh3
E
Filopodia density /µm
1
0.8
0.6
0.4
0.2
0
***
GFP
TAO1 sh 1
TAO1 sh 2
TAO1 sh 3
TAO1/2 sh
F
shRNA:
Ctrl
TAO1/2
MST3#1
MST3#2
TAO1
TAO2
MST3
Tubulin
G
Filopodia density / µm
1
0.8
0.6
0.4
0.2
0
***
***
GFP
TAO1/2 shRNA
TAO2A
H
TAO1/2 shRNA
8DIV
I
PSD95 containing protrusions/ µm
0.8
0.6
0.4
0.2
0
*
**
Control
MST3 shRNA
TAO1/2 shRNA

Figure S5

A

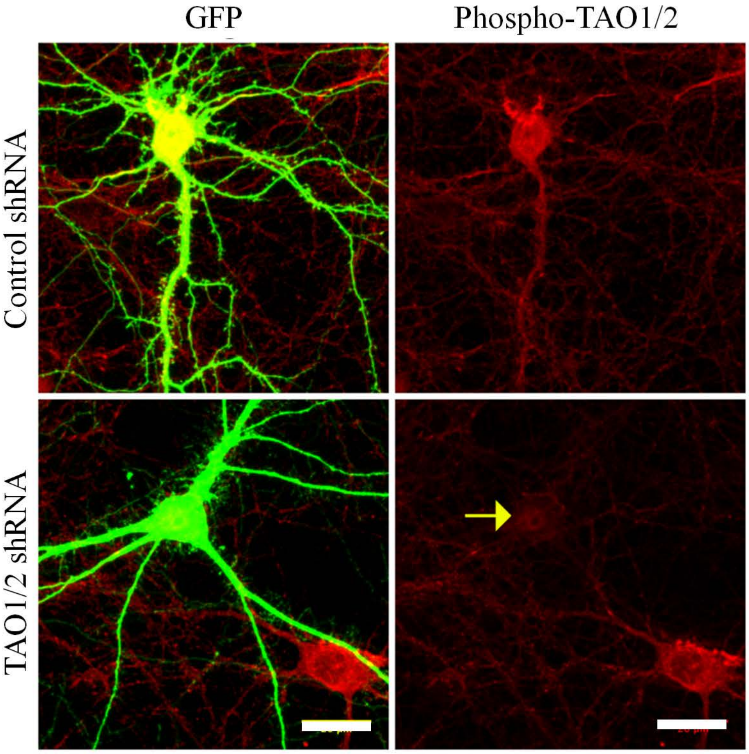

Figure S6

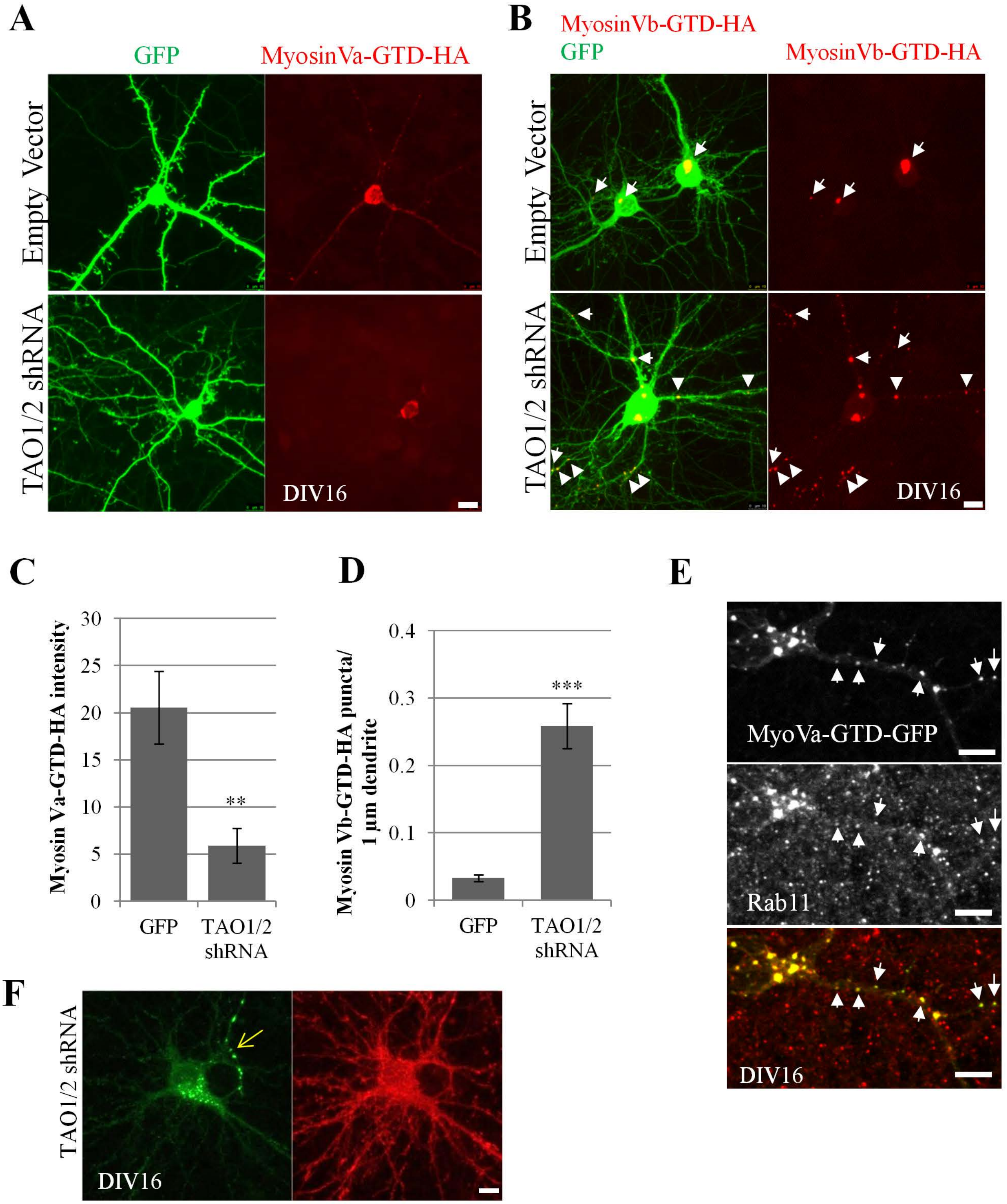

A
GFP
MyosinVa-GTD-HA
Empty Vector
TAO1/2 shRNA
DIV16
B
MyosinVb-GTD-HA
GFP
MyosinVb-GTD-HA
Empty Vector
TAO1/2 shRNA
DIV16
C
Myosin Va-GTD-HA intensity
30
25
20
15
10
5
0
**
GFP
TAO1/2 shRNA
D
Myosin Vb-GTD-HA puncta/ 1 µm dendrite
0.4
0.3
0.2
0.1
0
***
GFP
TAO1/2 shRNA
E
MyoVa-GTD-GFP
Rab11
DIV16
F
TAO1/2 shRNA
DIV16

SUPPLEMENTAL FIGURE LEGENDS

Figure S1: A& B. MST3 staining in cell bodies of neurons in hippocampal cultures are reduced. Empty vector or MST3 shRNA expressing vector transfected neurons are stained with a mouse monoclonal MST3 antibody. The staining in the cell body is significantly reduced (arrows and dotted line around the soma) ($p<0.001$) (n = 5 neurons each). Scale bar = 25 µm. C. Average filopodium length is also increased upon loss of function of MST3. D. Total filopodia length per 50 µm is not altered in MST3* (wild type MST3 that is shRNA resistant) expressing neurons (n = 6 for GFP and 5 for MST3*). E. Control shRNA does not alter length filopodia (n = 6 for GFP and 7 for control shRNA). F. Lentivirus was generated for pLentiLox 3.7 vector alone or vector expressing control shRNA (Ctrl sh), MST3 sh1 and MST3 sh2. Neurons were infected at DIV6 and protein lysates were obtained at 16 DIV. Endogeneous MST3 expression levels are reduced confirming knock-down (using rabbit polyclonal MST3 antibody). Tubulin antibody is used as a control for total protein levels. MST3 migrates at 60 kDa. Also see Figure S4F. G. Expression of HA tagged MST3-KD and HA-MST3* is detected by anti-HA antibodies for all data acquisition. Scale bar = 25 µm. H. MST3 shRNA #2 significantly increases dendrite total filopodia length per dendrite length, comparable to the first MST3 shRNA used (n = 7 neurons each). All error bars reflect standard errors.

Figure S2: A. GFP and MST3 shRNA expressing hippocampal neurons (magenta) immunostained with PSD-95 and Synapsin antibodies (teal) are shown. Colocalizations are shown in white. B. In MST3 shRNA expressing neurons protrusions co-localizing with synapsin is reduced $p < 0.05$. Similarly, in MST3 shRNA expressing neurons protrusions co-localizing with PSD-95 is reduced $p < 0.001$ (n = 5 cells each). All error bars reflect standard errors.

Figure S3: A. MST3-MG and MST3-KD expression and purification in HEK293 cells shown in coommassie staining. Half of the protein purified from a single 10 cm dish is loaded on the gel. About 8 µg of MST3-KD or MST3-MG is purified from one 10 cm plate. We used 3 or 4 plates for each construct in each experiment, therefore 25 – 30 µg of kinase is used for substrate labelling. B. Mass spectra of EPS8 and C. TAO2 phosphorylation site containing peptides are shown.

Figure S4: A. Hippocampal neurons transfected at DIV4 and imaged at DIV8 after GFP and HA immunostaining. TAO1wt, TAO1A and TAO1D are expressed in cytoplasm in cell body and dendrites. Scale bar = 25 µm. B. Filopodia density and total filopodia length per 50 µm of dendrite are shown for an experiment where neurons were transfected at DIV3 and imaged at DIV7. MST3 shRNA and TAO1A increases these measurements significantly (n = 21, 9, 14, 12 and 12 for GFP, MST3 shRNA, TAO1A, TAO1D and TAO1WT, respectively). C. TAO1A expression increases dendritic filopodia length when compared to GFP alone and TAO1D in hippocampal neurons transfected at DIV4 and imaged at DIV8 (n = 7, 7 and 10 for GFP, TAO1A, TAO1D, respectively). D. Representative images of dendrites expressing three separate TAO1 shRNAs (which do not target

TAO2) are shown. Scale bar is 7.5 µm. E. None of the three TAO1 shRNAs has any significant effect on filopodia density when expressed from 3DIV to 7DIV (n = 5, 6, 6, 5 and 6 neurons for GFP, TAO1sh1, TAO1sh2, TAO1sh3 and TAO1/2shRNA, respectively). TAO1/2 significantly increases filopodia density as shown earlier. F. Lentivirus expressing shRNA as labelled are used to infect cultured neurons at DIV6 and neuronal lysates were collected at DIV11. MST3 shRNAs are highly effective as shown earlier, TAO1/2 shRNA can effectively knock down TAO2 but also reduces TAO1 levels. Proteins migrate at, TAO1 120-130 kDa, TAO2 140-150 kDa, MST3 60 kDa. G. Filopodia density is also increased in TAO1/2 shRNA and TAO2A expressing neurons (n = 14, 14 and 6 neurons for GFP, TAO1/2 shRNA and TAO2A). H. PSD95-GFP and Td-tomato was expressed with TAO1/2 shRNA in hippocampal neurons. Scale bar = 5 µm. I. PSD95-GFP containing dendritic protrusion density is reduced in TAO1/2 shRNA expressing neurons (analysis also shown in Figure 2C & D). All error bars reflect standard errors.

Figure S5: PhosphoTAO1/2 immunostaining of hippocampal neurons transfected with GFP and control shRNA or GFP and TAO1/2 shRNA expressing plasmids is shown. Neurons were transfected at DIV11 and imaged at DIV16. TAO1/2 shRNA expression reduces phosphoTAO1/2 staining (arrow).

Figure S6: A. MyosinVa-GTD tagged with HA is co-expressed with a vector expressing GFP alone or GFP with TAO1/2 shRNA in dissociated cortical neurons. GFP is shown in green, HA antibody staining is shown in red. Images are maximum z projections of image stacks obtained by confocal imaging. Myosin Va-GTD-HA is localized to dendrites in control neurons but is restricted to the cell body in TAO1/2 shRNA expressing neurons. Cortical neurons were transfected at DIV 13-14 and fixed in 3 to 4 days. B. Myosin Vb- GTD-HA (red) is expressed in neurons with GFP or GFP together with TAO1/2 shRNA. The Myosin Vb- GTD puncta shown in red (right) is seen as yellow due to colocalization with GFP inside the neurons (left). Scale bars are 10 µm. C. Quantification of Myosin Va-HA intensity per pixel along one primary dendrite in neurons expressing GFP alone or TAO1/2 shRNA and GFP. In TAO1/2 shRNA expressing neurons Myosin Va-GTD-HA is reduced ($p < 0.01$). D. TAO1/2 shRNA causes increase in Myosin Vb-GTD-HA puncta in dendrites. Number of Myosin Vb-GTD-HA puncta is increased in TAO1/2 shRNA expressing neurons ($p < 0.001$). E. MyosinVa-GTD-GFP expressed in dissociated cortical neurons and stained by anti-Rab11. MyosinVa-GTD-GFP (green) is colocalized with Rab11 (red). Scale bar = 10 µm. F. In a subset of TAO1/2 shRNA expressing neurons, it can be clearly observed that axon is the only process that contains Myosin Va-GTD-GFP (yellow arrow). Scale bar = 10 µm. All error bars reflect standard errors.

Table S1:

The enrichment of proteins in Experiment 1 is shown as – values and in Experiment 2 as positive values. The columns are ordered with ascending median log2 value for Experiment 2. The number of unique peptides identified from each protein is shown in #peptides columns. Proteins that are enriched in phosphopeptide pulldown are shown in bold at the bottom of the list.

TABLE S1

Gene name	Uniprot Accession #	Protein Name	Experiment 1 median log2 (H/L)	# peptides	Experiment 2 median log2 (H/L)	# peptides
Hspa8	P63018	Heat shock cognate 71 kDa protein	-0.399124982	2	-1.365110729	2
Rps20	P60868	40S ribosomal protein S20	-0.147020233	2	-0.937942616	1
Rpl12	P23358	60S ribosomal protein L12	-0.261511777	7	-0.864809085	4
Srp9	D4A511	Signal recognition particle 9 kDa protein	-0.443591724	2	-0.733479106	1
Hist1h1e	P15865	Histone H1.4	-0.06698165	3	-0.731815826	2
Hspa5	P06761	78 kDa glucose-regulated protein	-0.443625045	24	-0.69294758	28
Hspa9	F1M953	Stress-70 protein, mitochondrial	-0.782047029	2	-0.688937034	29
H2afj	A9UMV8	Histone H2A.J	-0.953828454	1	-0.657363542	2
Grn	G3V8V1	Granulin, isoform CRA_c	0.137863359	6	-0.560106019	6
Spna2	C9EH87	Alpha II spectrin	-1.504200858	3	-0.556748581	1
Hspa8	P63018	Heat shock cognate 71 kDa protein	-0.499018389	34	-0.515123643	8
Rps13	P62278	40S ribosomal protein S13	-0.240927449	6	-0.50418133	2
Rps18	P62271	40S ribosomal protein S18	-0.272004482	8	-0.482541652	2
Rpl21	Q6PDW2	Protein LOC100361103	-0.098665616	4	-0.479671233	4
Rps18	P62271	40S ribosomal protein S18	-0.115569915	18	-0.417530945	10
Srp14	B2RYW7	Protein Srp14	0.651605447	2	-0.395293714	1
Rpl19	P84100	60S ribosomal protein L19	-0.094733424	3	-0.380379305	1
Rpl13a	P35427	60S ribosomal protein L13a	-0.055335579	9	-0.344522964	5
Hsp90ab1	P34058	Heat shock protein HSP 90-beta	-1.048915281	2	-0.342736594	4
Cltc	P11442	Clathrin heavy chain 1	-1.418683334	28	-0.32279553	3
Rpl18	P12001	60S ribosomal protein L18	-0.04171673	8	-0.313292994	3
Rpl7a	P62425	60S ribosomal protein L7a	-0.152221951	8	-0.295471287	7
Rpl24	P83732	60S ribosomal protein L24	-0.288948651	5	-0.282371389	4
Rpl8	P62919	60S ribosomal protein L8	-0.104484463	8	-0.28186538	4
Rpl23a	P62752	60S ribosomal protein L23a	0.088265913	5	-0.278614251	1
Rps19	P17074	40S ribosomal protein S19	-0.298973579	7	-0.266913982	4
Rpl6	F1LQS3	60S ribosomal protein L6	-0.097194996	12	-0.257134943	4
LOC100363800	Q6PDV8	RCG31311	-0.032982984	3	-0.241216334	2
Rpl10	Q6PDV7	60S ribosomal protein L10	-0.145797284	6	-0.23514841	3
Rps6	P62755	40S ribosomal protein S6	-0.262738516	5	-0.233490003	3
Rps8	B2RYR8	40S ribosomal protein S8	-0.274414909	8	-0.224696283	4
Rpl3	P21531	60S ribosomal protein L3	-0.076637333	15	-0.218307598	2
Rps14	P13471	40S ribosomal protein S14	-0.210862718	4	-0.21216553	1
Rpl30	P62890	60S ribosomal protein L30	-0.1714008	7	-0.20820505	3
Rps16	B0K038	Rps16 protein (Fragment)	-0.307722791	7	-0.174471461	3
Rplp2	P02401	60S acidic ribosomal protein P2	-0.274890907	7	-0.156321318	4
Rpl17	P24049	60S ribosomal protein L17	-0.07974843	8	-0.135015907	5
Thoc4	D3ZXH7	Protein Thoc4	-0.07806751	3	-0.13136143	1
Tpi1	P48500	Triosephosphate isomerase	-0.87872201	3	-0.131101117	1
Rps27l	P24051	40S ribosomal protein S27-like	-0.574733351	2	-0.120547393	1
Rpl15	P61314	60S ribosomal protein L15	-0.031099906	9	-0.119268311	5
Rps24	P62850	40S ribosomal protein S24	-0.040546735	5	-0.11919208	2
Rpl14	Q63507	60S ribosomal protein L14	0.234782096	4	-0.118774354	3
Map1b	F1LRL9	Microtubule-associated protein 1B	-0.468395268	60	-0.114418933	30
Ywhag	P61983	14-3-3 protein gamma	-2.09769028	4	-0.113774966	4
Rps3	P62909	40S ribosomal protein S3	-0.273943724	13	-0.113394676	6
Rps25	P62853	40S ribosomal protein S25	-0.192274417	4	-0.095271837	2
Rpl10a	P62907	60S ribosomal protein L10a	-0.087682664	8	-0.083715703	6
Tubal3	F1LUM5	Protein Tubal3	-1.809313376	1	-0.0833177	1
Rpl18a	P62718	60S ribosomal protein L18a	-0.053583056	6	-0.07772285	4
Rpl9	P17077	60S ribosomal protein L9	-0.065834006	10	-0.07319407	3
Hspa4	F1LRV4	Heat shock 70 kDa protein 4	-0.612575296	13	-0.073036819	7
Rpl23	P62832	60S ribosomal protein L23	-0.134731598	4	-0.064737894	3
Eif2s2	Q6P685	Eukaryotic translation initiation factor 2, subunit 2	-0.503936359	1	-0.059827369	1
Dpysl2	P47942	Dihydropyrimidinase-related protein 2	-1.253256947	10	-0.05144462	1
Fau	P62864	40S ribosomal protein S30	-0.06504867	1	-0.042689416	1
Srp19	B2RZ66	Protein Srp19	-0.17917254	3	-0.04114428	2
Rps7	P62083	40S ribosomal protein S7	-0.143824671	5	-0.032098054	4
Rpl27a	P18445	60S ribosomal protein L27a	-0.150563345	5	-0.012631752	2
Rps11	P62282	40S ribosomal protein S11	-0.119886604	7	-0.007417429	1
Rplp0	P19945	60S acidic ribosomal protein P0	-0.017583379	9	0.003599478	2
Spock1	F1M6V6	Protein Spock1 (Fragment)	-0.711211518	3	0.006528259	3
Rpl36al	B2RYQ8	Large subunit ribosomal protein L36a, isoform CR	-0.185730203	1	0.015658366	1
Gpc2	P51653	Glypican-2	-0.037629539	15	0.031070441	7
Rps15	P62845	40S ribosomal protein S15	-0.033111539	4	0.046244442	2
Rps3a	P49242	40S ribosomal protein S3a	-0.318526482	12	0.052180488	1
LOC100361060	D3ZZ95	60S ribosomal protein L36	-0.176532923	5	0.060384571	3
Atp5o	Q06647	ATP synthase subunit O, mitochondrial	-1.500896589	1	0.073727114	1
Ncl	P13383	Nucleolin	-0.272889451	16	0.075806424	8
Rpl32	P62912	60S ribosomal protein L32	-0.054293606	6	0.090573252	2
Rplp1	P19944	60S acidic ribosomal protein P1	-0.198654787	5	0.095049451	3
Cfl1	P45592	Cofilin-1	-1.572351763	2	0.102591096	4
Ncam1	F1LNY3	Neural cell adhesion molecule 1 (Fragment)	-1.800193522	6	0.11494048	1
Mapt	A0JN25	Microtubule-associated protein	-1.184820572	4	0.127122607	2
	Q00715	Histone H2B type 1	-0.41356892	4	0.132987233	2
Rps4x	P62703	40S ribosomal protein S4, X isoform	-0.119472201	15	0.14115858	4
Gapdh	P04797	Glyceraldehyde-3-phosphate dehydrogenase	-1.367866921	6	0.145520803	4
Calm1	P62161	Calmodulin	-0.718590137	5	0.196528507	5
Hnrnpu	Q63555	SP120	-0.435388651	7	0.196644436	3
Set	B0BMV1	Set protein	-0.645332691	4	0.212513847	3
Slc25a4	Q05962	ADP/ATP translocase 1	-1.117753287	8	0.232408924	6
Tuba1a	P68370	Tubulin alpha-1A chain	-1.078601992	1	0.256041071	1
Ywhae	P62260	14-3-3 protein epsilon	-2.559589067	3	0.286264436	4
Tubb6	Q4QQV0	Protein Tubb6	-2.091860172	2	0.294778133	1

Dcx	G3V997	Neuronal migration protein doublecortin	-1.101307055	6		0.296488546	4
Spock2	D3ZGJ7	Protein Spock2	-0.012452992	7		0.301046308	3
Pdia6	Q63081	Protein disulfide-isomerase A6	-0.665437468	2		0.33025525	3
Rps17	P04644	40S ribosomal protein S17	-0.130694285	5		0.334985206	2
Map1lc3a	Q6XVN8	Microtubule-associated proteins 1A/1B light chair	-1.265554368	1		0.338755877	1
Tubb2a	P85108	Tubulin beta-2A chain	-1.149510052	1		0.392077352	1
Tubb4a	B4F7C2	Protein Tubb4a	-1.091121976	4		0.45044024	3
Atp5b	G3V6D3	ATP synthase subunit beta	-1.524593296	14		0.460329855	3
Gpc1	P35053	Glypican-1	-0.457648402	2		0.514882773	1
Eef1a1	P62630	Elongation factor 1-alpha 1	-1.226260648	7		0.522591038	6
Rps5	B0BN81	Ribosomal protein S5, isoform CRA_b	-0.310358241	7		0.525872328	1
Tubb3	Q4QRB4	Tubulin beta-3 chain	-0.966886877	9		0.529305758	9
Tubb5	P69897	Tubulin beta-5 chain	-0.836486106	4		0.54397738	4
Txndc12	B0BN97	Txndc12 protein (Fragment)	-0.82770812	5		0.56331322	4
Map2	P15146	Microtubule-associated protein 2	-0.697944675	2		0.587798797	1
Ywhaz	P63102	14-3-3 protein zeta/delta	-2.515144732	5		0.628137753	5
Hist1h4b	P62804	Histone H4	-0.727142319	2		0.668213658	2
Atp5j2	D3ZAF6	ATP synthase subunit f, mitochondrial	-1.083544429	1		0.732193194	2
Ptges3	B2GV92	Ptges3 protein	-0.692101651	1		0.739069989	3
Ptma	P06302	Prothymosin alpha	-0.459784655	3		0.79872543	3
Naca	B2RYX0	Naca protein	2.965399988	1		0.823722255	1
Rpl39	P62893	60S ribosomal protein L39	-0.17300442	2		0.825780943	1
Ptms	B3DM95	Parathymosin	-0.214443584	2		0.943979344	1
Syt1	P21707	Synaptotagmin-1	-0.474652716	5		0.982138522	2
Npm1	P13084	Nucleophosmin	-0.405452998	1		1.115839424	2
Nap1l4	Q5U2Z3	Nucleosome assembly protein 1-like 4	-0.560560592	1		1.45371248	1
Tubb4b	Q6P9T8	Tubulin beta-4B chain	-0.686514448	1		1.458275542	1
Gpc4	Q642B0	Glypican 4	0.184623513	10		2.039738658	2
Tubb2b	Q3KRE8	Tubulin beta-2B chain	-1.02436493	1		2.042634012	1
Ppm1g	Q8K3W9	Protein phosphatase 1G (Formerly 2C), magnesiur	-1.13921528	1		2.133729301	1
Actb	P60711	Actin, cytoplasmic 1	-1.145454531	1		2.281321553	1
**Myo5a**	**Q9QYF3**	**Unconventional myosin-Va**	**-2.069855733**	**2**		**2.328145267**	**50**
**Dbn1**	**C6L8E0**	**Drebrin E**	**-1.380815233**	**1**		**2.660874407**	**11**
**Myl6**	**B2GV99**	**Myl6 protein**	**-1.856566114**	**2**		**2.988525252**	**9**

SUPPLEMENTAL EXPERIMENTAL PROCEDURES

Neuronal cultures

Hippocampal neurons were cultured from E19 Long-Evans rats (Charles River Lab) and plated at a density of 150,000 neurons per 18 mm glass coverslips (Fisher) coated with 0.06 mg/ml poly-D-lysine (Sigma) and 0.0025 mg/ml laminin (Sigma). Neurons were plated with plating media containing 10% Fetal bovine serum (Hyclone), 0.45% dextrose, 0.11 mg/ml sodium pyruvate, 2 mM glutamine in Modified Eagle Medium. Cultures were transferred to maintenance media after 4 hours containing 1X B27 (Invitrogen), 100 units/ml penicillin and 100 mcg/ml streptomycin, 0.5 mM glutamine, 12.5 µM glutamate in Neurobasal Media (Invitrogen). Half of the media was replaced with fresh media every 4 days. Neurons were transfected with ~ 0.5 µg plasmid DNA using Lipofectamine-2000 (Invitrogen) following manufacturer's guidelines.

DNA constructs, shRNA and lentiviruses

Mouse MST3 (BC004650) cDNA purchased from ATCC was cloned in pRK5 mammalian expression vector with N-terminal HA tag. MST3 kinase dead K53R and MST3 analog sensitive mutations M99A and M99G were generated by site directed mutagenesis. A MST3 shRNA#1 resistant construct (MST3*) was created by introducing silent mutations changing 6 base pairs resulting in 5'GGGCCTAGACTATCTTCAC3' by site directed mutagenesis. TAO1 resistant to TAO1/2 shRNA was generated by site directed mutagenesis introducing silent mutations changing 6 base pairs resulting in 5'GGGCAGTACGACGGAAAG3'. An shRNA with no substantial match to mouse or rat mRNA sequences targeting 5'AGACCCAAGGATTAGAAGG 3' was used as a control (shCtrl). Myristoylation tagged membrane targeted Td-tomato was cloned in pcDNA3.0 expression vector. Human FMNL2 (BC167159), human ArhGAP18 (BC111940), mouse GIPC (BC003490), mouse EPS8 (BC016890), human TAO1 (shorter isoform 853 amino acids, BC144067), human PAK6 (BC035596) were purchased from Open Biosystems/ Thermo Scientific and cloned into Prk5-HA vector via PCR cloning with NotI and SalI sites in frame with N-terminal HA tag. Human full length TAO2 (1235 amino acids) in pCMV Sport 6.0 expression vector was a gift from Dr. Froylan Calderon De Anda and Dr. Li-Huei Tsai from MIT (de Anda et al., 2012). Phosphorylation site mutations to Alanine or Aspartate were generated with site-directed mutagenesis. Myosin Va globular tail domain-GFP, Myosin Va- GTD-HA and Myosin Vb -GTD-HA vectors were generous gifts from Dr. Don Arnold from University of South California (Lewis et al., 2009). Drebrin pEGFP-C1 was a gift from Dr Wiebke Ludwig-Peitsch. PSD-95-GFP construct was a gift from Roger Nicoll's lab at UCSF.

All shRNA sequences were 19 base pairs long and were selected via http://katahdin.cshl.org/html/scripts/main.pl. shRNA target sequences on MST3 were sh #1 5'AGGACTTGATTATCTACAC3' and sh #2 5' GAAAGGACTTGATTATCTA3'. shRNA targeting sequences for rat TAO1 are: 5'CTAAGAGTTTGAAGTCTAA3', 5'CTTAGAACATGCAATGTTA 3'and 5' TGGAGAAACTTATTAAGAA 3'; rat EPS8 are 5'CCAGAGTGTTCAGTCAAA3', 5'TACTTGATGCCAAGGGTAA 3'and 5'CCTGGCTCTCAAGTCAACC 3' and for human MST3 used in HEK293T cells are 5'CAGTCCATATGTAACCAAA 3'and 5' CAGTGTTTATCTACAATTA 3'. Rat TAO2 and to a lesser extend TAO1 was knocked down by TAO1/2 shRNA with sequence: 5' GGGACAATATGATGGCAAA 3', other two rat TAO2 shRNAs are 5' GAGAGGACTTGAATAAGAA3 and 5'CACCCACAGTCATCATGGA 3'. Hairpins targeting these sequences were cloned in pLentiLox 3.7 which expresses EGFP via a separate promoter in addition to expressing shRNA via a U6 promoter. Empty pLentiLox 3.7 vector was used as a cell fill to visualize neurons and referred to as GFP. Lentivirus expressing empty pLentiLox 3.7 and expressing control shRNA, MST3 sh#1, MST3 sh#2, TAO1/2 shRNA #1 were generated at UCSF core facilities. All clones were verified by sequencing.

In Utero Electroporation

Timed pregnant C57Bl6 mice were used for *in utero* electroporation for the first set of experiments with MST3 shRNA. Surgery was performed at E14.5-15.5, when mice were anesthetized with sodium pentobarbital at 50 mg/kg body weight. A midline incision was made and the uterus was exposed. DNA solution including the plasmid and 0.04% trypan blue was injected into the lateral ventricle with a glass micropipette. After injection, embryos were subjected to electrical pulses (32Vfor E14.5, 36V for E15.5) with 50 ms duration that were delivered five times at 950 ms interval using a square-pulse electroporator BTX830. Embryos were injected with either 1 μg/μl pCAG-GFP (control) or 0.4 μg/μl pCAG-GFP + 1 μg/μl MST3 shRNA #1 in pLenti-Lox 3.7. For a second set of experiments timed pregnant Parkes mice were used. In these experiments animals were anaesthetized with isofluorane during the surgery. Embryos were injected with either 1 μg/μl pCAG-GFP (control) or 0.4 μg/μl pCAG-GFP + 1 μg/μl MST3 shRNA #1 or 0.4 μg/μl pCAG-GFP + 1 μg/μl TAO1/2 shRNA in pLenti-Lox 3.7. After the procedure the abdominal wall and skin were sutured to allow the embryos to develop full term. Litters were inspected for GFP expression in the cortex by fluorescent goggles in first week (Nightsea). Mice were perfused at P18-P20 using 4% paraformaldehyde and 4% sucrose. Brains were post-fixed in the same fixative solution at 4°C. 100 μm thick coronal sections were obtained using cryostat sectioning. Sections were dried on glass slides, blocked by 10% Normal Donkey Serum and 0.5% Triton-X and immunostained by mouse GFP antibody (1:1000, Roche) in blocking buffer, followed by goat anti-mouse conjugated with Alexa-488 (1:500, Invitrogen).

Immunocytochemisty

Following antibodies were used for immunostainings in cultured neurons: chicken anti-GFP (1:2000) Aves Labs, mouse anti-MST3 (1:1000) BD Transduction Labs, rabbit anti-MAP2 (1:1000) Chemicon, rat anti-HA 1:500 (Roche), mouse anti-PSD95 (1:200) Thermo-Fisher (6G6-1C9), mouse anti-Rab11 (1:100) BD Transduction Labs, rabbit anti-Synapsin I (1:1000) Millipore, rabbit anti-phospho TAO1/2 1:500. Rabbit polyclonal phospho TAO1/2 antibody was raised against peptide CNRDHFAT*IRTASL of moue TAO2 with phospho T 475 by Yenzym (San Francisco, CA).

Western Blots

We have used rat anti-HA 1:2000 (Roche), mouse anti-MST3 (1:1000) BD Transduction Labs and rabbit anti-MST3 (1:1000) Epitomics, mouse anti-TAO1 (1:1000) BD Biosciences, mouse anti-beta tubulin (1:1000) Covance, goat-anti TAO2 (1:1000) Santa Cruz and rabbit anti-thiophosphate ester (1:5000- 10,000) Epitomics. HRP conjugated secondary antibodies (Jackson Immunoresearch) were used.

Confocal Microscopy

Dendrites were imaged using an inverted Leica SP5 confocal microscope using a 63X (NA 1.4) objective at 6X zoom. Z sections were obtained across the dendrite depth at 0.5 μm z- intervals. Spine density and categorization was done manually using Leica image analysis. Spine head diameter measurements were done using a custom plugin in ImageJ (Ultanir et al., 2007).

Quantification of PhosphoTAO1/2 Staining in Cultures

Cultured rat hippocampal neurons were transfected with either control scrambled shRNA or shRNA against MST3 at DIV13 using Lipofectamine2000, and then fixed using 4%PFA+4% sucrose at DIV16 for 10 min at room temperature. Neurons were then incubated with blocking solution (10% normal donkey serum+ 0.2M glycine+0.2% triton x-100 in PBS) for an hour followed by incubation with primary antibody against p-TAO1/2 (rabbit polyclonal) and GFP (monoclonal from Roche) at 4 degrees overnight. After 3 washes with PBS, neurons were incubated for 2 hours at room temperature with Alexa 488 and 568 secondary antibodies. Coverslips were mounted using fluoromont after six washes with PBS. Imaging was performed on a scanning disk confocal SP5 Leica system using 40X, 1.25N objective at 1.5 times zoom. Image stacks were acquired at z spacing of 0.4 microns. Quantification of pTAO staining was performed using ImageJ Measure function. Average projection of Image stacks were acquired using ZProject. The fluorescence in the neuronal soma which was chosen as ROI was measured using Measure function in ImageJ. The fluorescence intensity of untransfected neighboring neuronal soma which was absent in GFP staining was used as internal control in each image. The normalized fluorescence intensity of neuronal soma from ten neurons per

experiment was calculated and the average of three different experiments was then plotted. Two tailed-unequal variance Student t-test was used to calculate p-values.

MST3 Kinase assays

HA-tagged MST3 was expressed in COS-7 or HEK293T cells for 48 hours which are maintained in a medium containing 10% FBS, 1 X penicillin/ streptomycin in Dulbecco's Modified Eagle Medium. MST3 kinase was purified via HA epitope tag using Anti-HA Affinity matrix (Roche, clone 3F10). Prior to lysis cells expressing MST3 (except kinase dead MST3) were incubated with 0.5 μM O.A. for 1 hour. Lysis buffer contained 1% Nonidet P-40, 10% glycerol, 1 mM Na3VO4, 20 mM β-glycerol phosphate, 50 mM NaF, 1 X complete protease inhibitor cocktail (Roche), 1 X phosphatase inhibitor cocktail I or III (Sigma) in 20 mM Tris-HCL pH 8.0 and 150 mM NaCl. Lysis was achieved by incubation on ice for 30 min. The cells were pipetted up and down several times before centrifugation at 20,000 g for 15 min at 4° C. Supernatant containing the solubilized proteins was precleared using IgG- Sepharose (GE Healthcare) for 30 min at 4° C. HA-MST3 was purified with HA-affinity matrix for 2 hours at 4° C. Beads were washed with lysis buffer twice, incubated with 1 M NaCl for 10 min followed by a 10 min wash with lysis buffer at 4° C. Beads were washed twice with a kinase reaction buffer not including ATP. Kinase assay was conducted in 20 mM Tris-HCl pH 7.5, 10 mM MgCl2, 1 mM dithiothreitol (DTT), 100 μM ATP, 1x Phosphatase inhibitor cocktail and 0.5 mM of one of the following four ATP analogs: ATP-γ-S, 6-Bn-ATP-γ-S (Bn), 6-PhEt-ATP-γ-S (Phe) or 6-Furfuryl-ATP-γ-S (Ff) (BioLog Life Science Institute) for 30 min at 30 ° C. Reaction was followed by alkylation for 1 hour at room temperature by addition of 2 μl 100 mM p-nitro mesylate (PNBM) per 30 μl of kinase reaction. Proteins were solubilized by sample buffer and ran at 4-12 % bis –tris gels (Invitrogen). MST3 autophosphorylation was detected by anti-thiophosphate ester antibody (1:10,000 – 1:5,000, Epitomics) followed by HRP conjugated secondary antibody.

MST3 Substrate Labelling

Covalent capture method for kinase substrate identification was done as previously described (Ultanir et al., 2012). Briefly, MST3 kinase was purified from COS-7 or HEK293T cells on HA-beads as described above. Cells expressing MST3-MG were treated with 0.5 μM okadaic acid for 1 hour. Untransfected cells and cells expressing MST3-KD were used as negative controls. Cells were lysed with 20 mM Tris HCL, 150 mM NaCl, Protease inhibitor cocktail, phosphatase inhibitor I (Sigma), 10% glycerol and 1% NP40. Lysate was incubated on ice for 30 min, followed by centrifugation at 20,000g for 15 min. Supernatant was pre-cleared with IgG sepharose beads. 50 μL 1:1 slurry anti-HA resin was incubated with protein lysate for 1,5 – 3 hours. P3 and P13 mouse brain lysates were obtained in 0.25% NP-40, 10 mM MgCl2, 100 Mm NaCl, 20 mM Tris pH=7.5, 0.5 mM

DTT and 1X protease inhibitor cocktail. 100 µl brain lysate of 20 µg/µl protein concentration was used to be labeled by MST3-MG or MST3-KD containing beads, each. A lysate only sample was used in which no HA beads were added as a second negative control for each experiment. Total 4 experiments were conducted twice with P13 and twice with P3 brain lysates. Labelling reaction contained 1 µM cyclic AMP- dependent protein kinase inhibitor, 10 µM protein kinase C inhibitor (Bisindolylmaleimide I – Calbiochem), 3 mM GTP, 100 µM ATP, 1 µM O.A and 0.5 mM Benzyl-ATP-γ-S.

Multiplex SILAC labelling of neuronal cultures

Cortical neuronal cultures were labelled using Multiplex SILAC as described (Zhang et al., 2011). Briefly, 5- 8 million cortical neurons from E18.5 rat embryos were plated per 10 cm culture dishes coated as described above. Same culture media was used as the hippocampal neurons with the exception of Neurobasal media free of L-Arginine and L-Lysine (Invitrogen) was used. Media was supplied with either Lysine 8 (U-13C6; U-15N2) and Arginine 10 (U-13C6; U-15N4) or Lysine 4 (4, 4, 5, 5-D4) and Arginine 6 (U-13C6). All isotopes were obtained from Cambridge Isotope Laboratories, Inc. 200 mg/L L-Proline was also included in the culture medium. Half of the culture media was replaced with new media every 3 -4 days. 1 µM Ara-C was included in the medium when the medium was replaced in to inhibit glial growth. Protein was extracted from three 10 cm dishes at DIV9 in 20 mM Tris pH = 8.0, 150 mM NaCl, 1% NP-40, 1 mM Okadaic acid and 1X protease inhibitor. Total 2.4 mg protein was obtained in a volume of 300 µl for Lys4Arg6 and Lys8Arg10 each.

Phospho-Peptide pull-down

Peptide pull-down using SILAC labeled lysates was described before (Stephanowitz et al., 2012). TAO2 T475 peptide (C RNRDHFAT*IRTASLVSR) was synthesized with or without a phosphorylation at the indicated Threonine (Elim Biopharmaceuticals). Peptide was conjugated to iodoacetyl agarose beads (Sulfolink Resin, Thermo Scientific) as described in manufacturer's protocol. 30 µl resin conjugated to 22 nmol of peptide was used to for pull-down in 1.6 mg SILAC labeled protein lysate. Beads were incubated with lysate for 1.5 hours at room temperature. Beads were washed four times with lysis buffer and two additional times with lysis buffer without NP40 or protease inhibitors. Precipitated proteins were subjected to in-bead typsin digest.

Reversed-phase liquid chromatography-electrospray tandem mass spectrometry (LC-MS/MS) analysis

In-beads digestion. After incubation with the SILAC cell extracts, phospho and non phospho peptide conjugated beads were washed with the conjugation buffer twice, and then mixed. After a short spin

the supernatant was removed, and beads (approximately 60 µl) were resuspended in 100 µl 8M urea in the presence of 80 mM ammonium bicarbonate. Samples were reduced by incubating for 15 min at 60ºC in the presence of 2.5 mM DTT. After this, samples were alkylated by incubation with 3 mM iodoacetamide for 1h in the dark at room temperature. Remaining iodoacetamide was quenched by adding DTT to a final concentration of 3 mM, and incubating at 37 C for 15 min. Samples were then diluted to a final concentration of 2 M urea, and digested overnight at 37ºC using 4 µg sequencing grade modified trypsin (Promega, Madison, WI). Concentration of ammonium bicarbonate during digestion was 20 mM, and pH was confirmed to be around 8. After digestion, samples were added formic acid to a final concentration of 4%, and peptides were extracted using C18 ZipTips (Waters) according to the manufacturer's protocol. Eluates of the ZipTips were vacuum-evaporated, and peptides resuspended in 7 µl 0.1% formic acid in water.

Reverse-phase LC-MS/MS Analysis. The digests were separated by nano-flow liquid chromatography using a 75-µm x 150-mm reverse phase 1.7 µm BEH 130 C18 column (Waters) at a flow rate of 600 nL/min in a NanoAcquityTM Ultra performance UPLC system (Waters). Mobile phase A was 0.1% formic acid in water and mobile phase B was 0.1% formic acid in acetonitrile. Following equilibration of the column in 2% solvent B, an aliquot of each digest (5 µl) was injected, then the organic content of the mobile phase was increased linearly to 40% over 90 min, and then to 50% in 1 min. The liquid chromatography elute was coupled to a hybrid linear ion trap-Orbitrap mass spectrometer (LTQ-Orbitrap Velos, Thermo Scientific, San Jose, CA) equipped with a nanoelectrospray ion source. Spraying was from an uncoated 15-µm-inner diameter spraying needle (New Objective, Woburn, MA). Peptides were analysed in positive ion mode and in information-dependent acquisition mode to automatically switch between MS and MS/MS acquisition. MS spectra were acquired in profile mode using the Orbitrap analyzer in the m/z range between 350 and 1500. For each MS spectrum, the 6 most intense multiple charged ions over a threshold of 2000 counts were selected to perform HCD experiments. Product ions were analysed on the Orbitrap in centroid mode. A dynamic exclusion window of 0.5 Da was applied that prevented the same m/z from being selected for 45 s after its acquisition.

Peaklists were generated using PAVA in-house software (Guan et al., 2011), based on the RawExtract script from Xcalibur v2.4 (Thermo Fisher Scientific, San Jose, CA). The peak lists were searched in three separate searches against the rat subset of the UniProt database as of March 21, 2012, using in-house ProteinProspector version 5.10.10 (a public version is available on line). A randomized version of all entries was concatenated to the database for estimation of false discovery rates in the searches. Peptide tolerance in searches was 20 ppm for precursor and 20 ppm for product ions, respectively. Peptides containing two miscleavages were allowed. Carbamidomethylation of cysteine was allowed as constant modification; acetylation of the N terminus of the protein, pyroglutamate formation from

N terminal glutamine and oxidation of methionine, were allowed as variable modifications in initial searches. A second search was performed allowing also for 2H(4) labelling in lysine and 13C(6) labelling in arginine as constant modifications; and a third search allowing for 15N(2) 13C(6) labelling in lysine and 15N(4) 13C(6) labelling in arginine as constant modifications. In all cases, the number of modification was limited to two per peptide. The 3 searches merged into a single result file. A minimal ProteinProspector protein score of 20, a peptide score of 15, a maximum expectation value of 0.05 and a minimal discriminant score threshold of 0.0 were used for initial identification criteria. Protein hits were considered significant when at least two peptide sequences matched a protein entry and the Prospector score was above the significance level. For identifications based on one single peptide sequence with high scores, the MS/MS spectrum was reinterpreted manually by matching all the observed fragment ions to a theoretical fragmentation obtained using MS Product (Protein Prospector) (Clauser et al., 1999).

Quantification—SILAC quantification measurements were extracted from the raw data by Search Compare in Protein Prospector (http://prospector.ucsf.edu). Search Compare averaged together MS scans from -10 s to +30 s from the time at which the MS/MS spectrum was acquired in order to produce measurements averaged over the elution of the peptide. SILAC ratios (medium heavy/heavy) were calculated, and base 2 logarithms of these values were used. If quantitative data were available from isotopic envelopes identified as different charge states of the same peptide, the median of the log2 of the calculated SILAC ratios was used for that peptide. For proteins, the median of all the log2 ratios for peptides unique to that protein was calculated. Protein log2 ratios were plotted in a histogram with bin size 0.2, and log2 ratios were then corrected for differences in protein level between the heavy and medium heavy sample using the deviation from 0 of the mode of this distribution.

Co-immunoprecipitation in HEK293T cells

HEK293T cells were transfected with Myosin Va GTD-GFP or control GFP construct, HA tagged TAO1 wild type and HA tagged TAO1 phosphomutant T440A separately. Cell lysates were made in lysis buffer (20mM Hepes pH 7.4, 150mM NaCl, 1% Triton-X100, 0.5% Deoxycholate, 0.1% SDS, 2mM EDTA, 2mM DTT with protease inhibitor cocktail from Roche). Lysate from Myosin Va-GFP or control GFP transfected cells was incubated with Protein G beads prebound with monoclonal GFP antibody, and then lysates from either HA-TAO1-wildtype or HA-TAO1-A phosphomutant transfected cells were added and incubated for 3 hours at 4 degrees with rotation. Beads were then washed thrice with lysis buffer, twice with lysis buffer without detergent and then sample buffer was added. Samples were run after boiling on a 4-12% gradient Bis-Tris gel, transeferred on PVDF membranes and then blotted with antibodies against HA to detect presence of TAO1 and GFP to detect equal load of GFP or Myosin Va-GFP.

Immunoprecipitation from neuronal lysates

Briefly, DIV6 or DIV9 rat embryonic cultured hippocampal neurons were lysed in HKT buffer (20mM Hepes pH7.4, 150mM KCl, 0.5% TritonX100, 1mM DTT, 1mM EDTA and protease inhibitor cocktail) and incubated with Protein A beads for 30 min at 4 degrees. Pre-cleared supernatant was collected and incubated with Myosin Va antibody bound Protein A beads (Sigma) overnight at 4 ºC. Beads were then washed thrice with HKT buffer followed by three times with buffer without detergent. Beads were collected and boiled after addition of sample buffer before running on a 4-12% Bis-Tris gel. After transfer on to PVDF membrane, blots were probed with TAO1 mouse antibody (BD biosciences), Myosin Va rabbit antibody (Sigma) and pTAO1/2 rabbit antibody.

Statistics

Multiple data sets were compared using ANOVA and pairwise comparisons of datasets were done using student's t test using Excel. On figures * = $p < 0.05$, ** = $p < 0.01$ and *** = $p < 0.001$, in Student's t-test. Comparisons of percentage data in Myosin Va localization was done using chi-square test. All error bars are standard errors of the mean.

Supplemental References

Clauser KR, Baker, PR, Burlingame, AL. (1999). Role of accurate mass measurement (+/- 10 ppm) in protein identification strategies employing MS or MS/MS and database searching. Anal. Chem. 71, 2871–2873.

Guan S, Price JC, Prusiner SB, Ghaemmaghami S, Burlingame AL. (2011). A data processing pipeline for mammalian proteome dynamics studies using stable isotope metabolic labeling. Mol Cell Proteomics. (12):M111.010728.
